# Metal-free dehydropolymerisation of phosphine-boranes using cyclic (alkyl)(amino)carbenes as hydrogen acceptors

**DOI:** 10.1038/s41467-019-08967-8

**Published:** 2019-03-26

**Authors:** Nicola L. Oldroyd, Saurabh S. Chitnis, Vincent T. Annibale, Marius I. Arz, Hazel A. Sparkes, Ian Manners

**Affiliations:** 1 School of Chemistry, University of Bristol, Cantock’s Close, Bristol, BS8 1TS UK; 2 Department of Chemistry, Dalhousie University, 6274 Coburg Road, P.O. 15000, Halifax, NS B3H 4R2 Canada; 3 Department of Chemistry, University of Victoria, Victoria, BC V8W 3V6 Canada

## Abstract

The divalent carbene carbon centre in cyclic (alkyl)(amino)carbenes (CAACs) is known to exhibit transition-metal-like insertion into E–H σ-bonds (E = H, N, Si, B, P, C, O) with formation of new, strong C–E and C–H bonds. Although subsequent transformations of the products represent an attractive strategy for metal-free synthesis, few examples have been reported. Herein we describe the dehydrogenation of phosphine-boranes, RR’PH·BH_3_, using a CAAC, which behaves as a stoichiometric hydrogen acceptor to release monomeric phosphinoboranes, [RR’PBH_2_], under mild conditions. The latter species are transient intermediates that either polymerise to the corresponding polyphosphinoboranes, [RR’PBH_2_]_*n*_ (R = Ph; R’ = H, Ph or Et), or are trapped in the form of CAAC-phosphinoborane adducts, CAAC·H_2_BPRR’ (R = R’ = *t*Bu; R = R’ = Mes). In contrast to previously established methods such as transition metal-catalysed dehydrocoupling, which only yield P-monosubstituted polymers, [RHPBH_2_]_*n*_, the CAAC-mediated route also provides access to P-disubstituted polymers, [RR’PBH_2_]_*n*_ (R = Ph; R’ = Ph or Et).

## Abstract (web summary)

Polymers featuring *p*-block elements other than carbon are of interest for a range of applications, but access to highly-substituted examples remains challenging. Here the authors demonstrate that cyclic (alkyl)(amino)carbenes can mediate the dehydropolymerisation of phosphine-boranes, leading to the construction of P-disubstituted polyphosphinoboranes.

## Introduction

Polymers that feature *p*-block elements other than carbon in the main chain are interesting materials due to their potential uses as elastomers, etch resists in lithography, polyelectrolytes, ceramic precursors and in optoelectronics^1–4^. Earlier syntheses of inorganic polymers were achieved by the use of polycondensation and ring-opening methods^1,2,5^. Access to stable yet reactive, polymerisable, multiply bonded *p*-block monomers required for addition polymerisation remains a major challenge in the synthesis of inorganic polymers^6–8^. More recently, metal-catalysed coupling routes have been developed for accessing a broad range of inorganic macromolecules and materials^2,9–14^. In this context catalytic dehydrocoupling between main-group substrates has been shown to be a versatile method for the general formation of E–E′ bonds, which can be also used to access polymers via catalytic dehydropolymerisation^15–20^.

**Fig. 1:**
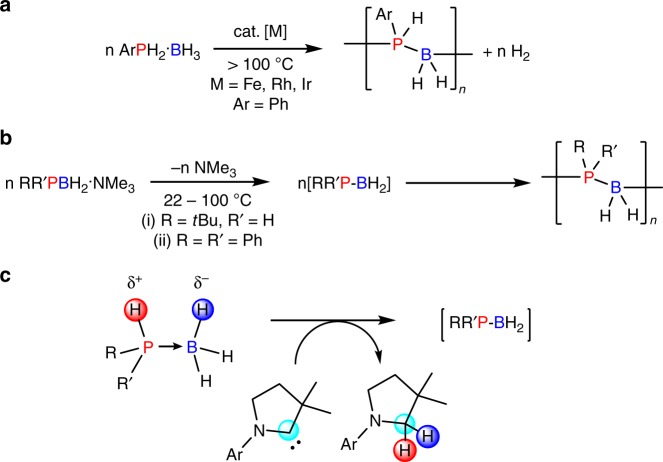
Synthesis of polyphosphinoboranes. **a**, **b** Current methods of synthesis and **c** proposed CAAC-mediated dehydrogenation

Polyphosphinoboranes attracted initial interest in the 1950s as a result of their potential as flame-retardant materials with high thermal stability^21–23^. However, attempts to dehydrocouple phosphine-borane adducts under thermal conditions yielded either low molecular weight or poorly soluble materials, which lacked convincing structural characterisation by modern standards^2,24,25^. Since 1999 a rhodium-catalysed dehydrocoupling approach to prepare soluble, high-molecular-weight (P-monosubstituted)polyphosphinoboranes has been available^26–28^. Examples of iron and iridium-catalysed dehydrocouplings have also been reported as routes to high-molecular-weight poly(arylphosphinoboranes) (Fig. 1a)^29,30^. Notably, these transition metal-catalysed protocols all require forcing conditions (≥100 °C, ≥20 h) and their scope is currently limited to the dehydrocoupling of primary arylphosphine-boranes, RPH_2_·BH_3_ (R = aryl). More recently, from our collaboration with Scheer and co-workers^31,32^, a metal-free synthesis of polyphosphinoboranes through the thermolysis of amine-stabilised phosphinoboranes, RR′PBH_2_·NMe_3_, was reported to proceed under milder conditions 22–40 °C. This route successfully produced high-molecular-weight poly-*tert*-butylphosphinoborane, [*t*BuHPBH_2_]_*n*_, presumably via the monomeric phosphinoborane [*t*BuHPBH_2_]. However, the precursors are challenging to prepare and attempts to access the P-disubstituted poly(diphenylphosphinoborane), [Ph_2_PBH_2_]_*n*_, by the thermolysis of Ph_2_PBH_2_·NMe_3_, yielded only very-low-molecular-weight oligomers [Ph_2_PBH_2_]_*x*_ (*x* ≤ 6) (Fig. 1b). The development of convenient and efficient dehydrocoupling of secondary phosphine-borane adducts to give the corresponding polymers therefore remains an open challenge. Herein, we demonstrate the successful use of the carbene centres of cyclic (alkyl)(amino)carbenes (CAACs) to mediate this process.

CAACs are analogues of *N*-heterocyclic carbenes (NHCs) with one of the electronegative amino substituents replaced by a strong σ-donating alkyl group, which simultaneously increases the nucleophilicity and electrophilicity at the divalent carbene carbon centre^33–36^. The resulting small HOMO–LUMO gap of CAACs has allowed E–H (E = H, N, Si, B, P, C, O) bond activation by formal oxidative addition to the carbene carbon centre for a variety of small molecules under mild conditions, with this process giving products featuring a H–C(sp^3^)–E moiety^37–42^. However, the strength of the resulting C(sp^3^)–H and C(sp^3^)–E σ−bonds disfavours further reactivity of the H–C(sp^3^)–E products, limiting the ability of CAACs to mimic transition metal centres in synthetic utility.

We envisioned that a CAAC-mediated dehydrogenation of primary and secondary phosphine-boranes, species that contain both protic P–H and hydridic B–H bonds, may be possible (Fig. 1c). Dehydrogenation of phosphine-boranes using this strategy leads to reactive phosphinoborane monomers, which, given appropriate substituents at the phosphorus and boron centres, gives soluble oligomeric and polymeric material.

## Results

### Reactivity of carbenes with phosphine-boranes

The synthesis of monomeric aminoborane-NHC adducts (NHC-BH_2_NHR) has been reported both through the use of an NHC for ambient temperature dehydrogenation of amine-boranes (RNH_2_·BH_3_; R = H, Me)^43^ and NHC-induced depolymerisation of poly(*N*-methylaminoborane)^44^. More recently, analogous species featuring the use of NHCs to stabilise phosphinoborane monomers have been isolated using NHC-induced thermal depolymerisation of polyphosphinoboranes^45^. Consequently, prior to investigating the reactivity of phosphine-boranes with CAACs, we explored the dehydrogenation potential of NHCs.

**Fig. 2:**
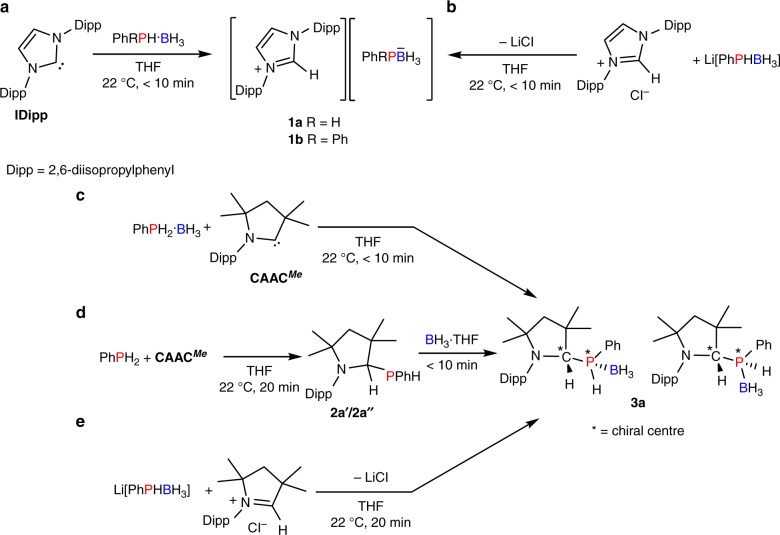
Reactivity of IDipp and CAAC^*Me*^ with phosphine-boranes. **a** Synthesis of **1a** and **1b** by deprotonation of the phosphine-borane using IDipp; **b** synthesis of **1a** and **1b** using salt metathesis route; **c** synthesis of **3a** through oxidative addition of PhPH_2_·BH_3_; **d** synthesis of **3a** through stepwise reaction of PhPH_2_, then BH_3_·THF; and **e** synthesis of **3a** through salt metathesis route

Upon addition of one equivalent of IDipp to a solution of PhPH_2_·BH_3_ in tetrahydrofuran (THF), a homogeneous solution was formed after 10 min, and analysis of the reaction mixture by ^31^P and ^11^B nuclear magnetic resonance (NMR) spectroscopy showed complete conversion to a new species (δ_P_ = −84.2ppm (br), δ_B_ = −33.4ppm (dq) in THF) (Supplementary Figs. 2 and 3). The similarity of these spectral features to those observed for Li[PhPHBH_3_] (δ_P_ = −93.8ppm (d), δ_B _= −34.6ppm (dq) in THF)^46^, an analogous compound with a different cation, is consistent with deprotonation of PhPH_2_·BH_3_ by IDipp to yield the salt [IDippH][PhHPBH_3_] (**1a**) (Fig. 2a). The formation of this salt was further confirmed by an independent synthesis via a metathesis reaction in THF between [IDippH]Cl and Li[PhHPBH_3_]. This showed ^11^B and ^31^P NMR spectral features that matched those assigned to **1a** along with precipitation of LiCl (Fig. 2b). The ^13^C NMR spectrum of **1a** showed no ^1^*J*_CP_ couplings involving the iminium carbon atom, which, together with the downfield chemical shift in the ^1^H NMR spectrum of the imidazolium proton (δ_H_ = 10.0ppm) (Supplementary Fig. 1), supports an ionic formulation for this species in solution. When Ph_2_PH·BH_3_ was reacted with IDipp, the analogous salt [IDippH][Ph_2_PBH_3_] (**1b**) was formed (Supplementary Figs. 4–6) and subsequently characterised using X-ray crystallography (Supplementary Fig. 7 and Supplementary Table 6).

Next, we attempted the analogous reaction with a CAAC as the smaller HOMO–LUMO separation of CAACs renders them potentially better candidates for E–H bond activations. The P–H activation of PhPH_2_·BH_3_ by one equivalent of CAAC^*Me*^ (Fig. 2c) occurred readily at 22 °C in THF to give **3a**, which exists as two diastereomers (**3a′** and **3a″**). The identity of **3a** was initially established based on a distinctive doublet of quartet of doublets coupling pattern observed in the ^1^H NMR spectrum for the P–H protons (Supplementary Figs. 8–10). This assignment was further corroborated by an independent synthesis via a stepwise procedure involving oxidative addition of PhPH_2_ to the carbene centre in CAAC^*Me*^ to yield **2a** (as a mixture of diastereomers each with indistinguishable enantiomers by NMR), followed by the addition of BH_3_·THF to give **3a** (Fig. 2d). The two diastereomers of **3a** were also formed immediately upon combining Li[PhHPBH_3_] and [CAAC^*Me*^H]Cl through elimination of LiCl (Fig. 2e). In contrast to the results obtained in the reaction of [IDippH]Cl and Li[PhPHBH_3_] above (Fig. 2b), the lower steric hindrance and greater π-acidity^36^ of the cation [CAAC^*Me*^H]^+^ leads to the formation of a molecular species with a distinct P–C bond, rather than the corresponding iminium salt [CAAC^*Me*^H][PhPHBH_3_]. The molecular formulation of **3a** is supported by the observation of both ^1^*J*_CP_ (^1^*J*_CP_ = 41.0 Hz (**3a′**), ^1^*J*_CP_ = 38.3 Hz (**3a″**)) and ^2^*J*_HP_ (^2^*J*_HP_ = 4.2 Hz (**3a′**), ^2^*J*_HP_ = 5.8 Hz (**3a″**)) coupling constants in the ^13^C and ^1^H NMR spectra.

Attempts to crystallographically characterise **3a** were unsuccessful as solutions in THF (0.10 M) spontaneously decomposed to a mixture of poly(phenylphosphinoborane) [PhHPBH_2_]_*n*_ and (CAAC^*Me*^)H_2_ as shown by ^1^H, ^11^B and ^31^P NMR spectroscopy. Although only sensitive to low molar mass fractions^47^, electrospray ionisation-mass spectrometry (ESI-MS) confirmed the formation of [PhHPBH_2_]_*n*_ (up to *n* = 22) by identifying repeat units of ∆(*m/*z) = 122.05 (molecular weight of [PhHPBH_2_] = 122.05 g mol^−1^). Isolation of pure [PhHPBH_2_]_*n*_ was achieved through precipitation of the reaction mixture into cold (−40 °C) hexanes to remove the hydrogenated carbene, (CAAC^*Me*^)H_2_ (Supplementary Figs. 25 and 26), which was also characterised by X-ray crystallography (Supplementary Fig. 27 and Supplementary Table 6). In the present case, the eliminated phosphinoborane monomer [PhHPBH_2_] polymerises, presumably due to the small size of the substituents at P and B.

The influence of temperature, solvent and concentration upon the molar mass of the poly(phenylphosphinoborane) obtained was systematically investigated with a view of optimising the polymerisation conditions (Table 1; Supplementary Table 1; and Supplementary Figs. 18–21, 23 and 24). In each case, ESI-MS and gel permeation chromatography (GPC) analyses were carried out (Supplementary Figs. 11–17, 22 and 23). ESI-MS clearly confirmed the presence of the [PhHPBH_2_] monomeric repeat unit in each case and allowed us to detect the presence of either BH_3_ or PPhH_2_ end groups (Supplementary Fig. 22). However, due to only the low molar mass fraction being detected by the method, it is not possible to draw links between the reaction conditions and the degree of polymerisation using these data^47^. In contrast, GPC analysis permitted optimisation of the polymerisation conditions as this technique reveals the complete molar mass distribution (Table 1 and Supplementary Table 1). Increasing the temperature (run 1 vs. 3, and 5 vs. 6) reduced the reaction time, but has no significant effect on the molar mass of the polymer obtained. Using a non-polar solvent (toluene) rather than THF (runs 3 vs. 5) also had no significant effect on the polymer molar mass. It was found that at higher concentrations (run 2 vs. 3 vs. 4), a larger quantity of polymeric relative to oligomeric material was formed (Supplementary Fig. 17). This observation is consistent with head-to-tail polymerisation of transiently generated phenylphosphinoborane, [PhHPBH_2_]. The reaction was also attempted under solvent-free, melt conditions at 110 °C (run 7), and, although high molar mass material was formed, the molar mass was no greater than that obtained using a concentrated solution at 60 °C. Due to concerns about the homogeneity of the reaction as a result of poor mixing, subsequent studies were performed in concentrated solutions rather than in the melt phase.

**Table 1:** Influence of temperature, solvent and concentration on the formation of poly(phenylphosphinoborane), [PhHPBH_2_]_*n*_, in a closed system


Run	Temp. (°C)	Solvent	Conc. (M)	Time (h)^a^	DP^b^	M_*n*_ (Da)^c^	PDI^c^
1	22	THF	0.50	120	205	25,000	1.55
2	60	THF	0.10	3	–^d^	–^d^	–^d^
3	60	THF	0.50	3	410	50,100	1.27
4	60	THF	1.26	3	686	83,800	1.13
5	60	Toluene	0.50	3	290	35,400	1.28
6	110	Toluene	0.50	0.5	230	28,000	1.52
7	110	None	N/A	3	302	36,800	1.39

*GPC* gel permeation chromatography, *THF* tetrahydrofuran, *N/A* not available, *NMR* nuclear magnetic resonance, DP degree of polymerisation, *PDI* polydispersity index

^a^Time taken for full conversion by ^31^P NMR spectroscopy

^b^DP measured by GPC

^c^Measured using GPC analysis

^d^No high-molecular-weight material recovered after precipitation

### Mechanistic studies

**Fig. 3:**
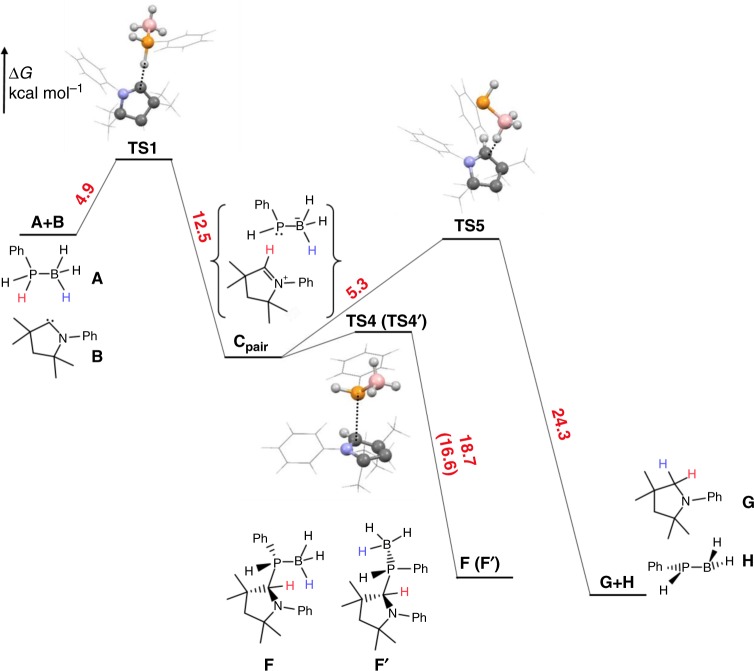
DFT study. Simplified schematic reaction profile calculated for the reaction of PhPH_2_·BH_3_ (**A**) with *N*-phenyl CAAC (**B**) at the PBE0/6-31 + G(d,p)/IEFPCM(THF) level of theory; Gibbs free energies for the second diastereomer are given in round brackets (for a comprehensive depiction of the reaction profile see Supplementary Fig. 31)

A series of experimental and density functional theory (DFT) studies have been undertaken to probe the mechanism of the dehydrogenation of PhPH_2_·BH_3_ with CAAC^*Me*^. Several mechanisms for the generation of monomeric [PhHPBH_2_] were considered and subsequently discounted, based on experimental and computational evidence (for a full discussion see ‘Proposed and subsequently discounted mechanisms for phosphine-borane dehydrogenation mediated by CAAC^*Me*^’ in the Supplementary Information; Supplementary Figs. 32 and 33; and Supplementary Tables 3 and 5), before the final mechanism shown below was proposed and supported (Fig. 3). Attempts to trap the released monomer with either cyclohexene^48^ or 1,3-cyclohexadiene^49^ proved unsuccessful.

Kinetic studies were conducted to assess the proposed mechanisms (Supplementary Table 2 and Supplementary Fig. 28). A plot of ln[**3a**] vs. reaction time showed equivalent half-lives of 1.5 h (Supplementary Fig. 29) for several initial concentrations between 0.3 and 0.7 M at 50 °C, indicating a first-order process in **3a**. Monitoring the reaction at several temperatures between 22 and 60 °C allowed the enthalpy and entropy of activation to be calculated as 21.5 kcal mol^−1^ and −9.5 cal K^−1^ mol^−1^, respectively, consistent with a substantial energy barrier involving a relatively ordered transition state (Supplementary Fig. 30).

DFT calculations were carried out at the PBE0/6-31 + G(d,p)/IEFPCM(THF) level of theory^50–52^ with an *N*-phenyl model system for the CAAC^*Me*^ (**B**) to further elucidate the dehydrogenation mechanism (Fig. 3). An initial deprotonation of the P–H bond of PhPH_2_·BH_3_ (**A**) with **B** to give a [CAAC(H)]^+^ and [PhPH(BH_3_)]^−^ ion pair (**C**_**pair**_) was the most favoured first reaction step with a low Gibbs free energy of activation of 4.9 kcal mol^−1^ (see ‘DFT calculations’ in the Supplementary Information). Subsequent nucleophilic attack at the iminium carbon of the [CAAC(H)]^+^ cation by the phosphorus centre of the [PhPH(BH_3_)]^−^ anion leads via **TS4** to the *S*_P_,*S* (**F**) diastereomer of the P–H activation product, or via **TS4′**, to the other *R*_P_,*S* (**F′**) diastereomer. The calculation of the activation barrier for this step was hampered by the inherently flat progression of the potential energy hypersurface between **TS4** or **TS4′** and **C**_**pair**_, which suggests, in agreement with the experimentally found rapid formation of **3a**, that this step occurs with a very small activation barrier. **F** and **F′** are kinetic products of the reaction. Significantly, this step is reversible via P–C dissociation, for which a maximum activation barrier of 18.7 kcal mol^−1^ was calculated from **F** to **TS4**. This opens up a second reaction pathway from **C**_**pair**_ leading to (CAAC)H_2_ (**G**) and [PhHPBH_2_] (**H**) via B–H hydride abstraction from the [PhPH(BH_3_)]^−^ anion by the π-acidic (CAAC-H)^+^ cation with a low activation barrier of 5.3 kcal mol^−1^ (via **TS5**). Thus, the formation of [PhHPBH_2_]_*n*_ from **3a** can be rationalised by the formation of transient [CAAC(H)]^+^ and [PhPH(BH_3_)]^−^ ions via consecutive P–C bond scission and B–H hydride abstraction leading to (CAAC^*Me*^)H_2_ and [PhHPBH_2_], the latter undergoing head-to-tail polymerisation to thermodynamically favoured [PhHPBH_2_]_*n*_ (Supplementary Fig. 31). For a discussion of the proposed polymerisation mechanism, see ‘Supplementary discussion of the polymerisation mechanism from phosphinoborane monomers’ in the Supplementary Information. The fact that the reaction between PhPH_2_·BH_3_ and IDipp stops at the [IDipp(H)]^+^ and [PhPH(BH_3_)]^−^ ions (Fig. 2a) can be traced back to the greater π-acidity of the [CAAC(H)]^+^ compared to the analogous [NHC(H)]^+^ cation, as suggested by the high exergonicity of the isodesmic reaction [CAAC(H)]^+^ + (NHC)H_2_ → [NHC(H)]^+^ + (CAAC)H_2_ (Δ*G*^0^ = −66.8 kcal mol^−1^; *N*-phenyl model systems) (Supplementary Table 4).

According to the calculations, the dissociation of the P–H activation products **F** or **F′** via the transition states **TS4** or **TS4′** requires the highest activation energy in the overall mechanism, which is in agreement with the first-order rate law found for **3a** by the kinetic measurements. In addition, the calculated enthalpy of activation for this step (Δ*H*^0^ = 20.2 (**TS4**), 19.2 (**TS4′**) kcal mol^−1^) is in good agreement with the substantial experimentally derived enthalpy of activation for the overall reaction (Δ*H*^0^ = 21.5 kcal mol^−1^). The higher Gibbs free energy of activation required for the dissociation of the *S*_P_,*S* diastereomer **F** (Δ*G*^0^ = 18.7 kcal mol^−1^) compared to the *R*_P_,*S* diastereomer **F′** (Δ*G*^0^ = 16.6 kcal mol^−1^) accounts for the experimentally observed faster conversion of one diastereomer during the reaction. Moreover, the observed enhanced reaction rates in THF (see Supplementary Table 2) can be rationalised by the better stabilisation of the [CAAC(H)]^+^ and [PhPH(BH_3_)]^−^ ions in THF than in toluene, which is further corroborated by the calculations (Supplementary Fig. 31).

### Substrate scope

**Fig. 4:**
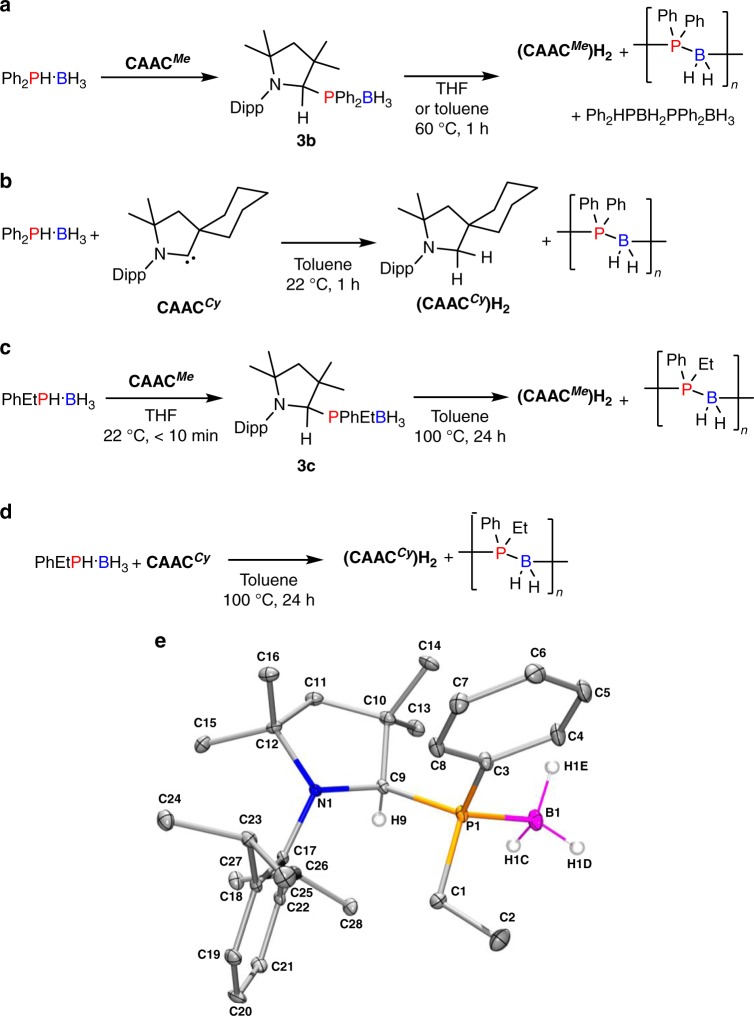
Reactions of Ph_2_PH·BH_3_ and *rac*-Ph(Et)PH·BH_3_ with CAAC^*Me*^ and CAAC^*Cy*^. **a** CAAC^*Me*^*-*mediated dehydrocoupling of Ph_2_PH·BH_3_; **b** CAAC^*Cy*^*-*mediated dehydrocoupling of Ph_2_PH·BH_3_; **c** CAAC^*Me*^-mediated dehydrocoupling of *rac*-Ph(Et)PH·BH_3_; **d** CAAC^*Cy*^*-*mediated dehydrocoupling of *rac*-Ph(Et)PH·BH_3_; and **e** thermal ellipsoid plot of **3c**. H atoms other than those bound to C9 and B1 have been omitted for clarity. Ellipsoids are shown at the 30% probability level

Given the success with PhPH_2_·BH_3_, the scope of the CAAC^*Me*^-mediated dehydropolymerisation was extended with the aim of targeting hitherto inaccessible high molar mass P-disubstituted polyphosphinoboranes. CAAC^*Me*^(H)Ph_2_PBH_3_ (**3b**) was synthesised from Ph_2_PH·BH_3_ and CAAC^*Me*^ in THF (Fig. 4a and Supplementary Figs. 34 and 35). Formation of **3b** was also detected immediately upon combining Li[Ph_2_PBH_3_] and [CAAC^*Me*^H]Cl, and also through the stepwise addition of Ph_2_PH followed by BH_3_·THF to a solution of CAAC^*Me*^.

Heating a concentrated solution of **3b** (2.5 M, 60 °C, 1 h, THF or toluene) effected complete conversion to (CAAC^*Me*^)H_2_, the linear dimer Ph_2_PHBH_2_PPh_2_BH_3_, cyclic oligomers [Ph_2_PBH_2_]_*x*_ (*x* = 3, 4), and the polymer [Ph_2_PBH_2_]_*n*_ as observed by ^1^H and ^31^P NMR (Fig. 4a and Supplementary Fig. 40a)^53^. Removal of (CAAC^*Me*^)H_2_ and cyclic oligomers was achieved by precipitation into hexanes, but attempts to separate Ph_2_PHBH_2_PPh_2_BH_3_ and [Ph_2_PBH_2_]_*n*_ proved unsuccessful (for details see ‘Dehydropolymerisation of Ph_2_PH·BH_3_’ in the Supplementary Information). ESI-MS analysis of the product after precipitation nevertheless confirmed the presence of the repeat unit ∆(*m/*z) = 198.08 (molecular weight of [Ph_2_PBH_2_] = 198.08 g mol^−1^, maximum value of *n* = 10) (Supplementary Fig. 36). However, GPC analysis showed only a very small amount of high molar mass material. Interestingly, when toluene, rather than THF, is used as the solvent, a much smaller quantity of linear dimer is formed (Supplementary Figs. 37 and 40b). Under these conditions, GPC analysis on the precipitated material showed a majority of low molar mass material (M_*n*_ = ca. 1,300; polydispersity index (PDI)  = 1.31) and a small amount (ca. 10%) of high molar mass material (M_*n*_ = 54,300; PDI = 1.12) (Supplementary Fig. 38).

With the aim of increasing the yield and amount of high molar mass material, we investigated the use of the more reactive CAAC^*Cy*^, exemplified by its ability to activate dihydrogen under mild conditions^37^. The initially formed P–H activation compound is consumed within 1 h at 22 °C (Fig. 4b). However, GPC analysis again showed only a small amount (ca. 12%) of high molar mass material (M_*n*_ = 59,600; PDI = 1.08) with the majority being low molar mass material (M_*n*_ = ca. 1100; PDI = 1.28) (Supplementary Figs. 39, 40c and 41–45).

In an attempt to further extend the scope of the dehydropolymerisation to other P-disubstituted phosphine-boranes the reactivity of *rac*-Ph(Et)PH·BH_3_ with CAAC^*Me*^ and CAAC^*Cy*^ was investigated. CAAC^*Me*^(H)PhEtPBH_3_ (**3c**) was formed through direct reaction of CAAC^*Me*^ with *rac*-PhEtPH·BH_3_ (Supplementary Figs. 46–50). Unlike with the mono- and di-phenyl derivatives, **3c** is stable at 22 °C, which allowed the structure to be confirmed by X-ray diffraction (Fig. 4e and Supplementary Table 6). Upon heating isolated **3c** to 100 °C, the targeted dehydropolymerisation occurred to give [PhEtPBH_2_]_*n*_ and (CAAC^*Me*^)H_2_ (Fig. 4c and Supplementary Fig. 51). Pure [PhEtPBH_2_]_*n*_ was obtained in 23% yield as a fine white powder following precipitation. ESI-MS analysis of the precipitated sample confirmed the presence of the repeat unit of [PhEtPBH_2_]_*n*_ (∆(*m/*z) = 150.08, molar mass of [PhEtPBH_2_] = 150.08 g mol^−1^) and *n* = 33 (Supplementary Fig. 52); however, there was no convincing high molar mass material observed using GPC. In the analogous reaction using CAAC^*Cy*^, the yield was also low (19%); however, a GPC peak corresponding to high molar mass material (M_*n*_ = 62,600, PDI = 1.19, *n* = ca. 400) was observed (Supplementary Figs. 53–58). Again this was only a small amount (ca. 18 %) compared to the low molar mass fraction (M_*n*_ = ca. 1900; PDI = 1.47, *n* = ca. 13). Upon closer analysis of the ESI-MS spectra for each synthesis of [Ph_2_PBH_2_]_*n*_ and [PhEtBH_2_]_*n*_, the end group of the major distribution was detected as being either CAAC^*Me*^ or CAAC^*Cy*^ (Supplementary Figs. 36, 37, 44, 52 and 56). These results suggest that trace amounts of CAACs may react with the polymer chain at some point during the polymerisation (for further discussion see ‘Supplementary discussion of the polymerisation mechanism from phosphinoborane monomers’ in the Supplementary Information).

When the reactivity of CAAC^*Me*^ with bulkier P-disubstituted phosphine-borane substrates (R = R′ = *t*Bu or R = R′ = Mes) was explored, an enlightening divergence in reactivity was noted. For full conversion of these substrates, two equivalents of CAAC^*Me*^ are required. In situ ^1^H NMR reveals that an equimolar mixture of (CAAC^*Me*^)H_2_ and the new species **4a**/**4b** are formed (Fig. 5a and Supplementary Figs. 59–68).

**Fig. 5:**
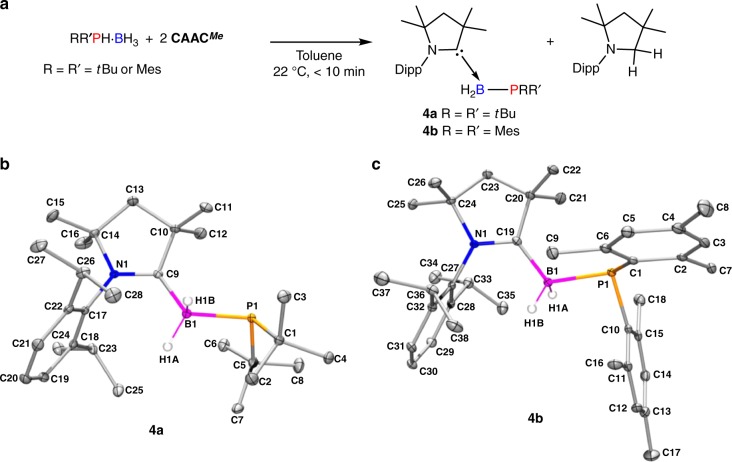
Synthesis and structure of cyclic (alkyl)(amino)carbene-phosphinoborane adducts **4a** and **4b**. **a** Synthesis of **4a** and **4b**; **b** thermal ellipsoid plot of **4a**; and **c** thermal ellipsoid plot of **4b**. For both **4a** and **4b** ellipsoids are shown at the 30% probability level, and H atoms other than those at the B1 centre have been omitted for clarity

The structures of **4a** and **4b** were confirmed by X-ray crystallography revealing that in both compounds the CAAC^*Me*^ C-donor was bound to the boron of the phosphinoborane moiety (Fig. 5b, c and Supplementary Table 7). Interestingly, species **4a** and **4b** are analogous to the previously mentioned NHC–phosphinoborane adducts that have been recently reported^45^.

The reactivity with the bulky, P-disubstituted phosphine-boranes contrasts with that observed with PhPH_2_·BH_3_, Ph_2_PH·BH_3_ and PhEtPH·BH_3_ as an initial P–H oxidative-addition product analogous to compounds **3a–c** is not observed. The monomeric phosphinoborane generated upon dehydrogenation does not undergo head-to-tail polymerisation, instead it is trapped by a second equivalent of carbene. The absence of an observable P–H activation compound can be explained by the greater steric bulk around the phosphorus centre. The trapping, however, provides further evidence for the release of monomeric phosphinoboranes in the proposed polymerisation mechanism. It is noteworthy that when Ph_2_PH·BH_3_ is reacted with two equivalents of CAAC^*Me*^ clean conversion to the species analogous to **4a** and **4b** is not observed; however, peaks for the short-chain oligomers CAAC(BH_2_PPh_2_)_*x*_ (*x* = 1–4) have been identified using ESI-MS (Supplementary Fig. 69).

## Discussion

In summary, we have shown that cyclic alkyl(amino)carbenes can be used as stoichiometric reagents to effect P–H/B–H dehydrogenative coupling of primary and secondary phosphine-boranes. These results illustrate the complementarity between organic and transition metal ambiphiles in the context of the main-group redox transformations, and hint at a potentially broad utility for CAACs in accessing new inorganic polymers and materials. The carbene centre in CAAC^*Me*^ inserts into the P–H bond of phosphine-boranes, RR′PH·BH_3_ (R = Ph; R′ = H, Ph, or Et), to give derivatives of CAAC^*Me*^(H)PRR′BH_3_ (**3a–c**) which undergo thermolysis to give the hydrogenated carbene (CAAC^*Me*^)H_2_ and polymers [RR′PBH_2_]_*n*_. Most remarkable is that in the case of Ph_2_PH·BH_3_ with CAAC^*Cy*^ the dehydropolymerisation proceeds within 1 h at 22 °C. In contrast, with respect to the reactivity of sterically encumbered P-disubstituted phosphine-boranes (R = R′ = *t*Bu or Mes) with CAAC^*Me*^, it is noteworthy that polymers are not generated post H_2_ transfer. Instead, the transient phosphinoboranes were trapped by a second equivalent of carbene to yield CAAC^*Me*^–phosphinoborane adducts, **4a** and **4b**. The novel dehydropolymerisation using CAACs has been used to prepare samples of P-disubstituted polyphosphinoboranes, [Ph_2_PBH_2_]_*n*_ and [PhEtPBH_2_]_*n*_, which cannot be accessed via previous transition metal-catalysed or stoichiometric routes, and contain high molar mass fractions. The development of catalytic rather than stoichiometric reactions involving main-group species is a rapidly developing field^54,55^. The reactions of phosphine-boranes with species that undergo E–H bond activation, for example, stannylenes^56^ and frustrated Lewis pairs that reversibly bind H_2_^57,58^, are under current investigation. Future studies will target the generation of a well-defined propagating site, which should allow access to predominantly linear polymers, molar mass control and potentially block copolymers. A more atom-economic catalytic synthesis would also allow a more facile scale-up and thereby the properties of the new materials to be investigated in detail.

## Methods

### Detailed procedure for polymerisation of PhPH_2_*·*BH_3_ using CAAC^*Me*^ (run 4)

PhPH_2_*·*BH_3_ (156 mg, 1.26 mmol) and CAAC^*Me*^ (360 mg, 1.26 mmol) were dissolved in THF (1 mL) in a J. Young Schlenk tube, sealed and the reaction mixture was stirred at 60 °C for 3 h. The reaction mixture was added dropwise into 20 mL of rapidly stirred cold hexanes at −40 °C, yielding a precipitate, and the supernatant was decanted. The precipitation was repeated twice more prior to drying in vacuo to leave a white powder of the [PhHPBH_2_]_*n*_ polymer product. Yield (precipitated material) = 42 mg (27%). GPC (2 mg mL^−1^): M_*n*_ = 83,800 Da; PDI = 1.17. Full experimental details for all polymerisations can be found in the Supplementary Methods.

## Supplementary information

Supplementary Information

## Data Availability

Crystallographic data for the structures reported in this article have been deposited at the Cambridge Crystallographic Data Centre, under deposition nos. CCDC 1867656–1867660.

## References

[1] Jäkle F (2010). Advances in the synthesis of organoborane polymers for optical, electronic, and sensory applications. Chem. Rev..

[2] Priegert AM, Rawe BW, Serin SC, Gates DP (2016). Polymers and the *p*-block elements. Chem. Soc. Rev..

[3] He X, Baumgartner T (2013). Conjugated main-group polymers for optoelectronics. RSC Adv..

[4] Jäkle, F. & Vidal, F. Functional polymeric materials based on main group elements. *Angew. Chem. Int. Ed*. 10.1002/anie.201810611 (2018). 10.1002/anie.201810611 30426641

[5] Fazen PJ (1995). Synthesis, properties, and ceramic conversion reactions of polyborazylene. a high-yield polymeric precursor to boron nitride. Chem. Mater..

[6] Chivers, T. & Manners, M. *Inorganic Rings and Polymers of the p-Block Elements* (Royal Society of Chemistry, London, 2009).

[7] Tsang CW, Yam M, Gates DP (2003). The addition polymerization of a P = C bond: a route to new phosphine polymers. J. Am. Chem. Soc..

[8] Pavelka, L. C., Holder, S. J. & Baines, K. M. Addition polymerization of 1,1-dimesitylneopentylgermene: synthesis of a polygermene. *Chem. Commun*. 2346–2348 (2008). 10.1039/b801762j 18473065

[9] He G (2013). The Marriage of Metallacycle Transfer Chemistry with Suzuki−Miyaura Cross-Coupling To Give Main Group Element-Containing Conjugated Polymers. J. Am. Chem. Soc..

[10] Leitao EM, Jurca T, Manners I (2013). Catalysis in service of main group chemistry offers a versatile approach to *p*-block molecules and materials. Nat. Chem..

[11] Linshoeft J (2014). Highly tin-selective stille coupling: synthesis of a polymer containing a stannole in the main chain. Angew. Chem. Int. Ed..

[12] McKeown GR (2016). Synthesis of macrocyclic poly(3-hexylthiophene) and poly(3-heptylselenophene) by alkyne homocoupling. ACS Macro Lett..

[13] Matsumura Y (2016). Arsole-containing π-conjugated polymer by the post-element-transformation technique. Angew. Chem. Int. Ed..

[14] Adams GM (2018). Dehydropolymerization of H_3_B·NMeH_2_ to form polyaminoboranes using [Rh(Xantphos-alkyl)] catalysts. J. Am. Chem. Soc..

[15] Melen RL (2016). Dehydrocoupling routes to element-element bonds catalysed by main group compounds. Chem. Soc. Rev..

[16] Johnson, H. C., Hooper, T. N. & Weller, A. S. In *Synthesis and Application of Organoboron Compounds*, Vol. 49 (eds Fernandez, E. & Whiting, A.) 153–220 (Springer, Springer International Publishing, New York, 2015).

[17] Colebatch, A. L. & Weller, A. S. Amine-borane dehydropolymerization: challenges and opportunities. *Chem. Eur. J*. **25**, 1379–1390 (2019). 10.1002/chem.201804592 PMC6391989 30338876

[18] Tilley DT (1993). The coordination polymerization of silanes to polysilanes by a ‘σ-bond metathesis’ mechanism. Implications for linear chain growth. Acc. Chem. Res..

[19] Imori T, Lu V, Cai H, Tilley TD (1995). Metal-catalyzed dehydropolymerization of secondary stannanes to high molecular weight polystannanes. J. Am. Chem. Soc..

[20] Choffat F (2007). Synthesis and characterization of linear poly(dialkylstannane)s. Macromolecules.

[21] Parshall, G. W. *The Chemistry of Boron and Its Compounds* (Wiley, New York, 1967).

[22] Mayer-Gall T, Knittel D, Gutmann JS, Opwis K (2015). Permanent flame retardant finishing of textiles by allyl-functionalized polyphosphazenes. ACS Appl. Mater. Interfaces.

[23] Priegert AM, Siu PW, Hu TQ, Gates DP (2015). Flammability properties of paper coated with poly (methylenephosphine), an organophosphorus polymer. Fire Mater..

[24] Burg A (1959). Phosphinoborine polymer rings and chains from tetramethylbiphosphine. J. Inorg. Nucl. Chem..

[25] Wagner RI, Caserio FF (1959). Linear phosphinoborane polymers. J. Inorg. Nucl. Chem..

[26] Dorn H, Singh RA, Massey JA, Lough AJ, Manners I (1999). Rhodium-catalyzed formation of phosphorus-boron bonds: synthesis of the first high molecular weight poly(phosphinoborane). Angew. Chem. Int. Ed..

[27] Pandey, S., Lönnecke, P. & Hey-Hawkins, E. Phosphorus-boron-based polymers obtained by dehydrocoupling of ferrocenylphosphine–borane adducts. *Eur. J. Inorg. Chem*. **2014**, 2456–2465 (2014).

[28] Cavaye H (2017). Primary alkylphosphine-borane polymers: synthesis, low glass transition temperature, and a predictive capability thereof. Macromolecules.

[29] Paul USD, Braunschweig H, Radius U (2016). Iridium-catalysed dehydrocoupling of aryl phosphine–borane adducts: synthesis and characterisation of high molecular weight poly(phosphinoboranes). Chem. Commun..

[30] Schäfer A (2015). Iron-catalyzed dehydropolymerization: a convenient route to poly(phosphinoboranes) with molecular-weight control. Angew. Chem. Int. Ed..

[31] Marquardt C (2015). Metal-free addition/head-to-tail polymerization of transient phosphinoboranes, RPH-BH_2_: a route to poly(alkylphosphinoboranes). Angew. Chem. Int. Ed..

[32] Stauber, A. et al. A convenient route to monoalkyl-substituted phosphanylboranes (HRP-BH_2_-NMe_3_): prospective precursors to poly[(alkylphosphino)­boranes]. *Eur. J. Inorg. Chem*. **2016**, 2684–2687 (2016).

[33] Lavallo V, Canac Y, Präsang C, Donnadieu B, Bertrand G (2005). Stable cyclic (alkyl)(amino)carbenes as rigid or flexible, bulky, electron-rich ligands for transition-metal catalysts: a quaternary carbon atom makes the difference. Angew. Chem. Int. Ed..

[34] Jazzar R (2007). Intramolecular ‘hydroiminiumation’ of alkenes: application to the synthesis of conjugate acids of cyclic alkyl amino carbenes (CAACs). Angew. Chem. Int. Ed..

[35] Bertrand G, Soleilhavoup M (2015). Cyclic (alkyl)(amino) carbenes (CAACs): stable carbenes on the rise. Acc. Chem. Res..

[36] Melaimi M, Jazzar R, Soleilhavoup M, Bertrand G (2017). Cyclic (alkyl)(amino) carbenes (CAACs): recent developments. Angew. Chem. Int. Ed..

[37] Frey GD, Lavallo V, Donnadieu B, Schoeller WW, Bertrand G (2007). Facile splitting of hydrogen and ammonia by nucleophilic activation at a single carbon center. Science.

[38] Frey GD, Masuda JD, Donnadieu B, Bertrand G (2010). Activation of Si-H, B-H, and P-H bonds at a single nonmetal center. Angew. Chem. Int. Ed..

[39] Mohapatra C (2016). Insertion of cyclic alkyl(amino) carbene into the Si−H Bonds of hydrochlorosilanes. Inorg. Chem..

[40] Turner ZR (2016). Chemically non-innocent cyclic (alkyl)(amino) carbenes: ligand rearrangement, C-H and C-F bond activation. Chem. Eur. J..

[41] Jin L (2016). Isolation of cationic and neutral (allenylidene)(carbene) and bis(allenylidene)gold complexes. Chem. Sci..

[42] Li Y (2013). Acyclic germylones: congeners of allenes with a central germanium atom. J. Am. Chem. Soc..

[43] Sabourin KJ, Malcolm AC, Mcdonald R, Ferguson MJ, Rivard E (2013). Metal-free dehydrogenation of amine–boranes by an *N*-heterocyclic carbene. Dalt. Trans..

[44] Stubbs NE, Jurca T, Leitao EM, Woodall CH, Manners I (2013). Polyaminoborane main chain scission using *N*-heterocyclic carbenes; formation of donor-stabilised monomeric aminoboranes. Chem. Commun..

[45] Marquardt C (2018). Depolymerization of poly(phosphinoboranes): from polymers to Lewis base stabilized monomers. Chem. Eur. J..

[46] Jaska CA, Lough AJ, Manners I (2004). Linear hybrid aminoborane/phosphinoborane chains: synthesis, proton-hydride interactions, and thermolysis behavior. Inorg. Chem..

[47] Staubitz A (2010). Catalytic dehydrocoupling/dehydrogenation of *N*-methylamine-borane and ammonia-borane: synthesis and characterization of high molecular weight polyaminoboranes. J. Am. Chem. Soc..

[48] Metters OJ (2014). Generation of aminoborane monomers RR′N = BH2 from amine-boronium cations [RR′NH-BH_2_L]( + ): metal catalyst-free formation of polyaminoboranes at ambient temperature. Chem. Commun..

[49] Breunig JM, Hübner A, Bolte M, Wagner M, Lerner HW (2013). Reactivity of phosphaboradibenzofulvene toward hydrogen, acetonitrile, benzophenone, and 2,3-dimethylbutadiene. Organometallics.

[50] Frisch, M. J. et al. *Gaussian 09, Revision D.01*. (Gaussian, Wallingford, 2009).

[51] Adamo C, Barone B (1999). Toward reliable density functional methods without adjustable parameters: the PBE0 model. J. Chem. Phys..

[52] Hehre WJ, Ditchfield R, Pople JA (1972). Self-consistent molecular orbital methods. XII. Further extensions of gaussian-—type basis sets for use in molecular orbital studies of organic molecules. J. Chem. Phys..

[53] Dorn H (2000). Transition metal-catalyzed formation of phosphorus-boron bonds: a new route to phosphinoborane rings, chains, and macromolecules. J. Am. Chem. Soc..

[54] Weetman C, Inoue S (2018). The road travelled: after main-group elements as transition metals. ChemCatChem.

[55] Hong M, Chen J, Chen EY (2018). Polymerization of polar monomers mediated by main-group Lewis acid−base pairs. Chem. Rev..

[56] Protchenko AV (2016). Enabling and probing oxidative addition and reductive elimination at a group 14 metal center: cleavage and functionalization of E-H bonds by a bis(boryl)stannylene. J. Am. Chem. Soc..

[57] Mo Z, Rit A, Campos J, Kolychev EL, Aldridge S (2016). Catalytic B-N dehydrogenation using frustrated Lewis pairs: evidence for a chain-growth coupling mechanism. J. Am. Chem. Soc..

[58] Boudjelel M (2018). Catalytic dehydrogenation of (di)amine-boranes with a geometrically constrained phosphine-borane Lewis pair. ACS Catal..

