# Supplementary material for: Metal-free dehydropolymerisation of phosphine-boranes using cyclic (alkyl)(amino)carbenes as hydrogen acceptors
Source: Nat Commun. 2019 Mar 26;10:1370. doi: 10.1038/s41467-019-08967-8 (PMC6435733; doi:10.1038/s41467-019-08967-8)
Supplement: Supplementary file 1 — Supplementary Information [file 41467_2019_8967_MOESM1_ESM.pdf]

Supplementary Information

Metal-free dehydropolymerisation of phosphine-boranes using cyclic (alkyl)(amino)carbenes as hydrogen acceptors

Manners, I. et al.

Supplementary Notes

All manipulations were carried out either under an atmosphere of nitrogen gas using standard vacuum line and Schlenk techniques, or under an atmosphere of argon within an M. Braun glovebox MB150G-B maintained at < 0.1 ppm of H_2O and < 0.1 ppm of O_2. All solvents were dried via a Grubbs design solvent purification system, except tetrahydrofuran (THF) which was distilled over sodium metal/benzophenone ketyl.

Deuterated solvents (benzene-d_6, chloroform-d and tetrahydrofuran-d_8) were purchased from Sigma Aldrich and dried by storing over activated molecular sieves (4Å). NMR spectra were recorded using Oxford Jeol Eclipse 300 MHz, Bruker 400 MHz, Bruker 500 MHz and Varian 400 MHz NMR spectrometers. ^1H NMR spectra were calibrated using the residual protio signal of the solvent: (δ (ppm) $^1H(CHCl_3)$ = 7.24; ^1H (C_6D_6) = 7.16; and ^1H $(CD_2)_4O$ = 3.58, 1.72). ^{13}C–NMR spectra were calibrated using the solvent signals (δ = $^{13}C(C_6D_6)$ = 128.0). ^{11}B and ^{31}P NMR spectra were calibrated against external standards (^{31}P: 85% H_3PO_4 (aq) (δ ^{31}P = 0.0); ^{11}B: $BF_3{\cdot}OEt_2$ (δ ^{11}B = 0.0)).

IDipp,[1] [IDippH]Cl,[1] CAAC^{Me},[2] [CAAC^{Me}H]Cl,[2] CAAC^{Cy},[2] $PhPH_2{\cdot}BH_3$,[3] $Mes_2PH{\cdot}BH_3$,[4] *rac*-PhEtPHBH$_3$,[5] and Ph_2PHBH_3[6] were prepared according to literature procedures. $BH_3{\cdot}$THF (1 M in THF) was purchased from Acros Organics and trap-to-trap vacuum transferred prior to use. $PPhH_2$ (ca. 10% weight in hexanes), Ph_2PH, *t*Bu$_2$PH·BH$_3$, and *n*BuLi (1.6 M in hexanes) were purchased from Sigma Aldrich and used as received.

GPC was performed on a Malvern RI max Gel Permeation Chromatograph, equipped with an automatic sampler, a pump, an injector, and inline degasser. The columns (T5000) were contained within an oven (35 °C) and consisted of styrene/divinyl benzene gels. Sample elution was detected by means of a differential refractometer. THF (Fisher), containing 0.1 wt% [*n*Bu$_4$N]Br, was used as the eluent at a flow rate of 1 mL min^{-1}. Samples were dissolved in the eluent (2 mg mL^{-1}) and filtered with a Ministart SRP15 filter poly(tetrafluoroethylene) membrane of 0.45 µm pore size before analysis. The calibration was conducted using monodisperse polystyrene standards obtained from Aldrich. The lowest and highest molecular

weight standards used were 2,300 Da and 994,000 Da respectively. Data for low molar mass components (i.e. M_n < 2,300 Da) is estimated.

The ESI-MS spectra were obtained using a Waters Synapt G2S instrument equipped with a nanospray ionisation module (Advion TriVersa Nanomate). Solutions (40 µL) of approximately 1 mg mL^{-1} were loaded under ambient conditions in air into the sample tray, and aliquots of 3 µL were introduced into the spectrometer using a spray voltage of 1.5 kV. Positive and negative ion spectra were recorded at a rate of 1 scan $second^{-1}$ and summed to obtain the final spectra. Elemental analyses (C, H, N) were performed externally by Elemental Microanalysis Ltd. in Devon, UK.

Supplementary Methods

Synthesis of IDipp phosphidoborane salts

Synthesis of **1a**

Method A:

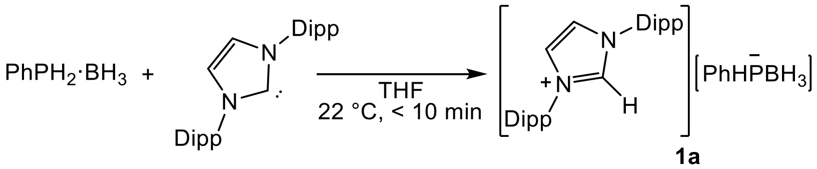

$PhPH_2{\cdot}BH_3$ (16 mg, 0.13 mmol) was dissolved in THF-d_8 (0.5 mL) in a quartz J. Young NMR tube and IDipp (50 mg, 0.13 mmol) added. Immediately a white precipitate was detected prior to the solution becoming homogeneous. The initial precipitate observed is attributed to the fast formation of the poorly soluble salt which is dissolved upon stirring. Crystals of **1a** were grown from a layered THF/hexane solution at -40 °C. However, the crystals were not of high enough quality to give a publishable data set, only a preliminary structure which confirmed the connectivity could be obtained. Yield = 38 mg (58 %).

Method B:

Independent synthesis of **1a** through salt metathesis

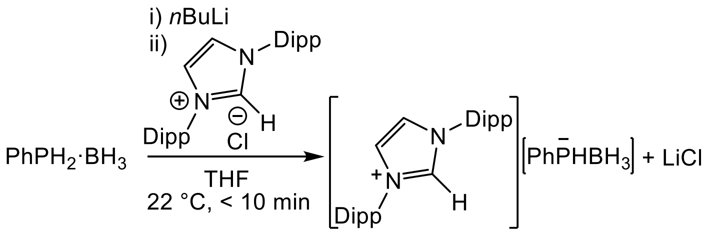

An *n*BuLi solution (630 µL of a 1.6 M in hexanes solution, 1.00 mmol) was added to a solution of $PhPH_2{\cdot}BH_3$ (124 mg, 1.00 mmol) in THF (1 mL) and allowed to stir for ten minutes at 22 °C before being added to a solution of [IDippH]Cl (435 mg, 1.00 mmol) in THF (1 mL). Immediate conversion to the product was observed and the identity confirmed using ^{1}H, ^{11}B and ^{31}P NMR spectroscopy. No attempts to isolate the product obtained through this route were made.

^{1}H NMR (400 MHz, 25 °C, THF-d_8): δ = 0.61 (m, 3H, B*H*), 1.20 (d, $^3J_{HH}$ = 6.9 Hz, 12H, CH(C*H*$_3$)$_2$), 1.24 (d, $^3J_{HH}$ = 6.9 Hz, 12H, CH(C*H*$_3$)$_2$), 2.53 (sept, $^3J_{HH}$ = 6.9 Hz, 4H, C*H*(CH$_3$)$_2$), 6.33-7.14 (m, ArH (Ph)), 7.39 (d, $^3J_{HH}$ = 7.7 Hz, Ar^m (Dipp), 4H), 7.57 (t, $^3J_{HH}$ = 7.7 Hz, Ar^p (Dipp)), 8.07 (s, 2H, NC*H*C*H*N), 10.05 (s, H, NC*H*). P–H proton cannot be detected in ^{1}H NMR spectrum.

^{11}B NMR (96 MHz, 22 °C, THF-d_8): δ = -33.4 (qd, $^1J_{BH}$ = 91.4 Hz, $^1J_{BH}$ = 26.6 Hz).

^{31}P NMR (122 MHz, 23 °C, THF-d_8): δ = -84.2 (m, br).

Elemental analysis for $C_{33}H_{46}BN_2P$ (calcd/expt): C (77.33/76.58), H (9.05/9.22), N (5.47/5.44).

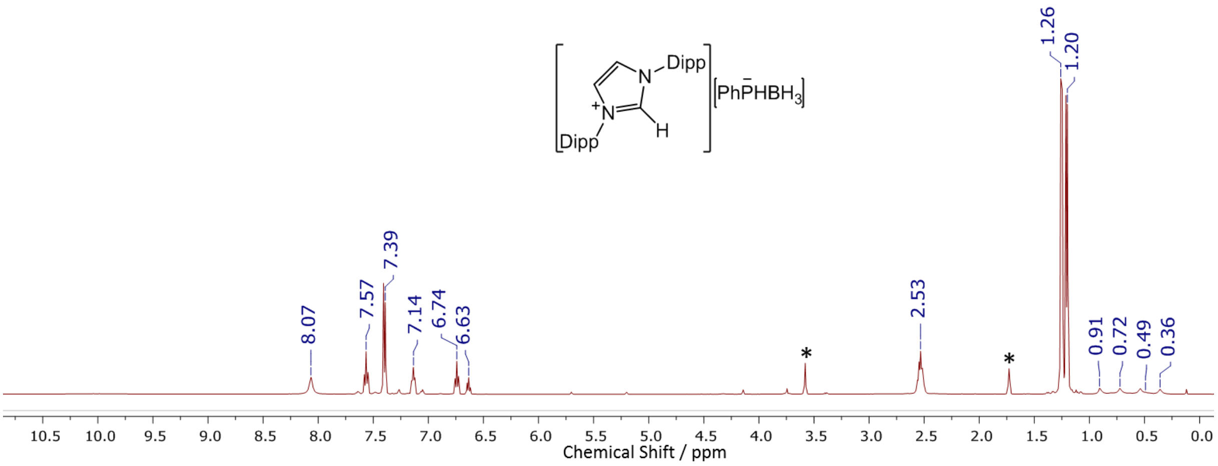

Supplementary Figure 1. ^{1}H NMR spectrum (400 MHz, 25 °C, THF-d_8) of **1a**. Iminium C-H proton is very broad. The iminium C-H proton resonance is 8.07. (*denotes residual partially protiated THF).

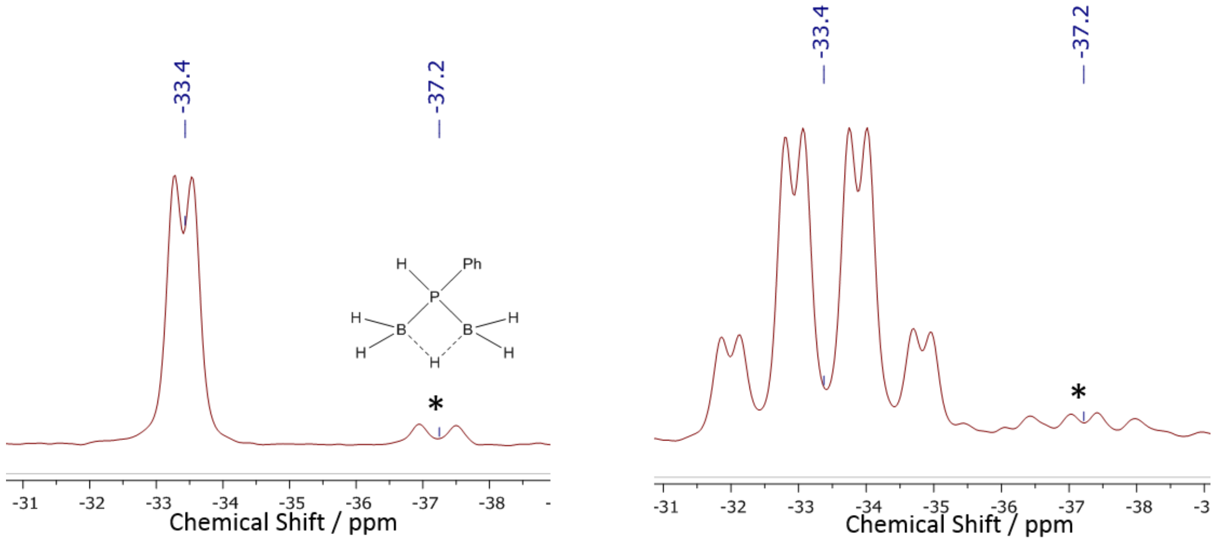

Supplementary Figure 2. $^{11}B\{^{1}H\}$ (left) and ^{11}B (right) NMR spectra (96 MHz, 22 °C, THF-d_8) of **1a** (*denotes trace μ-(PhPH)B_2H_5).

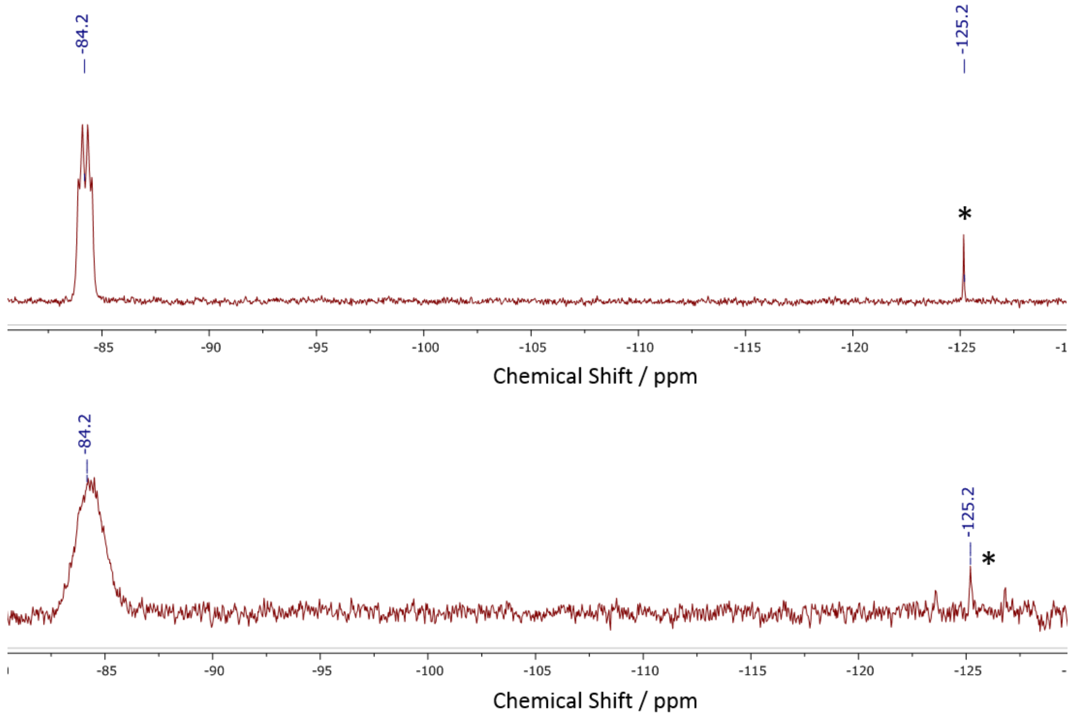

Supplementary Figure 3. $^{31}P\{^{1}H\}$ (top) and ^{31}P (bottom) NMR spectra (122 MHz, 23 °C, THF-d_8) of **1a** (* denotes trace $PhPH_2$).

Synthesis of **1b**

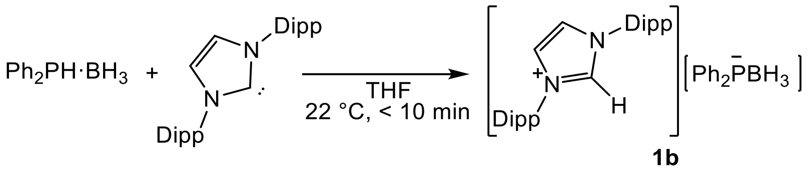

$Ph_2PH_2·BH_3$ (24 mg, 0.13 mmol) and IDipp (50 mg, 0.13 mmol) were dissolved in THF-d_8 (0.5 mL) in a quartz J. Young NMR tube and immediately a white precipitate was detected prior to the solution becoming homogeneous. The solvent was removed *in vacuo* to give a white solid which was washed with hexanes to give the product. Crystals of **1b** suitable for X-ray crystallography were grown from a layered THF/hexane solution at -40 °C. Yield prior to crystallisation = 40 mg (53 %).

^{1}H NMR (400 MHz, 25 °C, THF-d_8): δ = 0.98 (m, 3H, B*H*), 1.18 (d, $^3J_{HH}$ = 6.9 Hz, 12H, CH(C*H*$_3$)$_2$), 1.21 (d, $^3J_{HH}$ = 6.9 Hz, 12H, CH(C*H*$_3$)$_2$), 2.73 (sept, $^3J_{HH}$ = 6.9 Hz, 4H, C*H*(CH$_3$)$_2$), 7.25 – 7.33, 7.39-7.47, 7.55-7.62 (m, aromatic and NC*H*C*H*N backbone). C–H proton cannot be detected in ^{1}H NMR spectrum.

^{11}B NMR (96 MHz, 22 °C, THF-d_8): δ = -34.4 (m).

^{31}P NMR (122 MHz, 23 °C, THF-d_8): δ = -21.6 (br).

Elemental analysis for $C_{39}H_{50}BN_2P$ (calcd/expt): C(79.58/79.04), H (8.56/8.74), N(4.76/4.72).

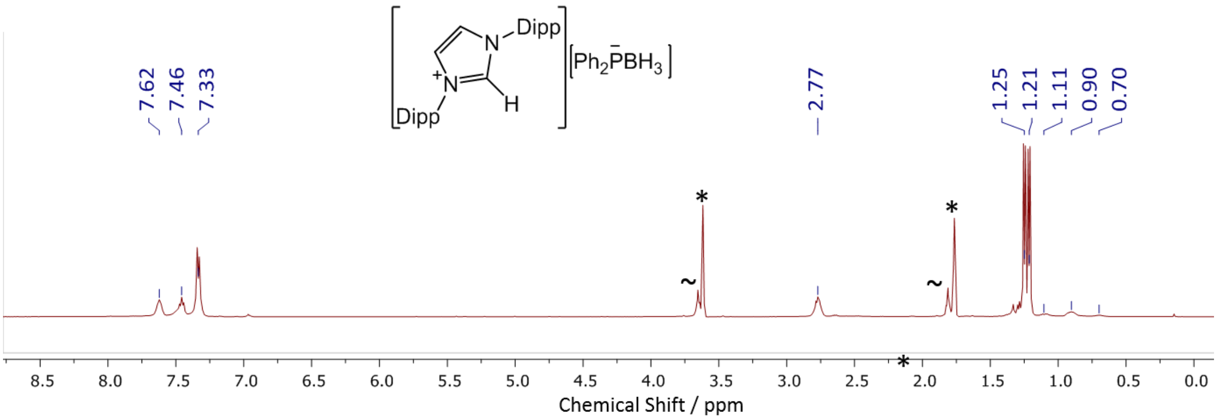

Supplementary Figure 4. ^{1}H NMR spectrum (400 MHz, 25 °C, THF-d_8) of **1b** (*denotes residual partially protiated THF, ~ denotes THF).

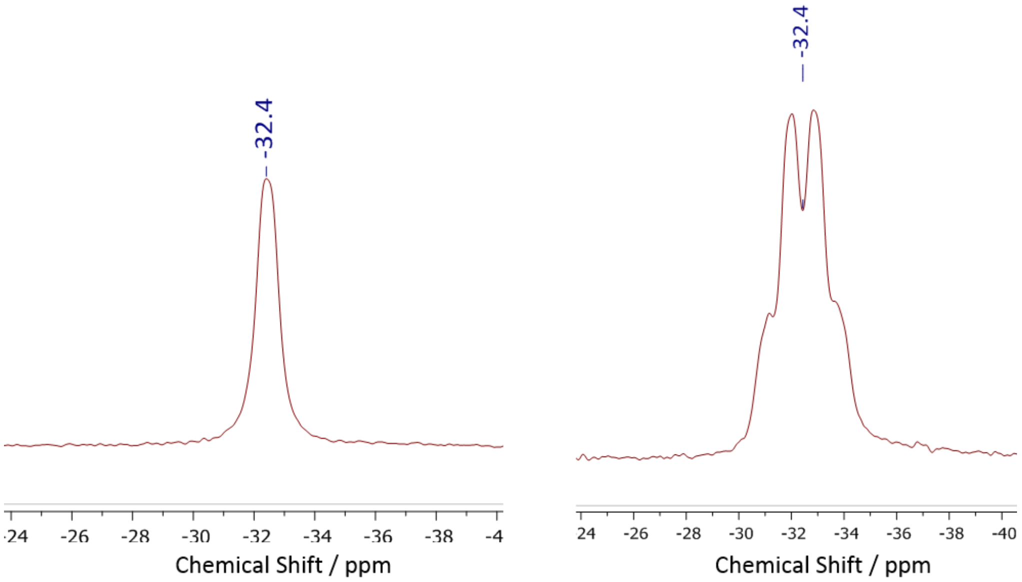

Supplementary Figure 5. $^{11}B\{^{1}H\}$ (left) and ^{11}B (right) NMR spectra (96 MHz, 22 °C, THF-d_8) of **1b**

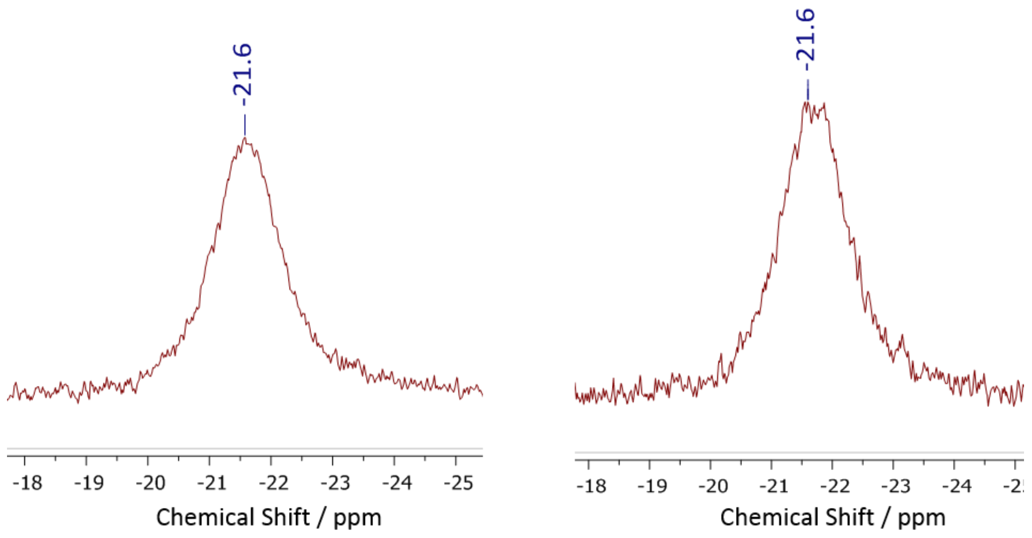

Supplementary Figure 6. $^{31}P\{^1H\}$ (left) and ^{31}P (right) NMR spectra (122 MHz, 23 °C, THF-d_8) of **1b**

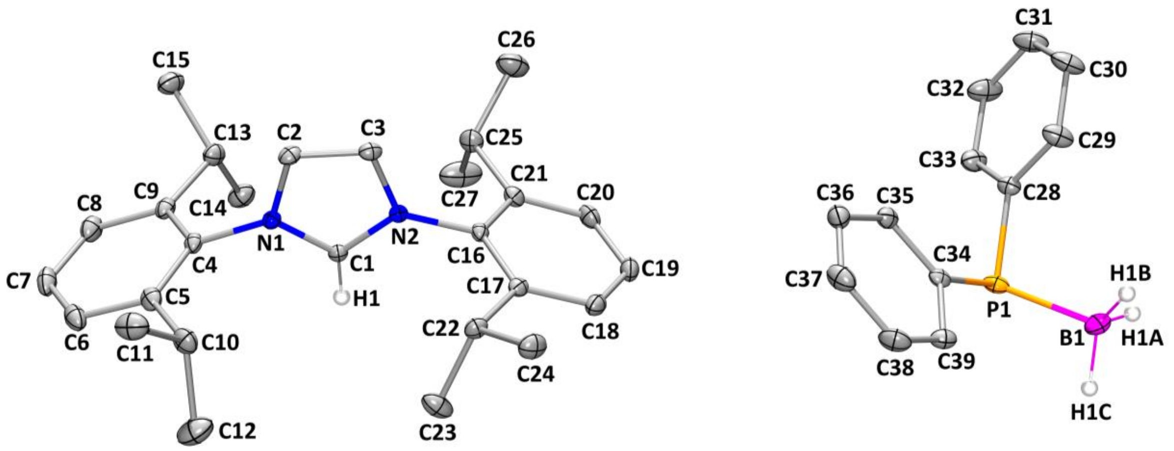

Supplementary Figure 7. Thermal ellipsoid plot of **1b**. Ellipsoids are shown at the 30% probability level. Second molecule of **1b** and THF solvent molecules from the asymmetric unit, along with H atoms other than those bound to C1 and B1 have been omitted for clarity.

Polymerisation attempts of $PhPH_2{\cdot}BH_3$ using CAAC^{Me}

<u>Synthesis of **3a**</u>

Method A:

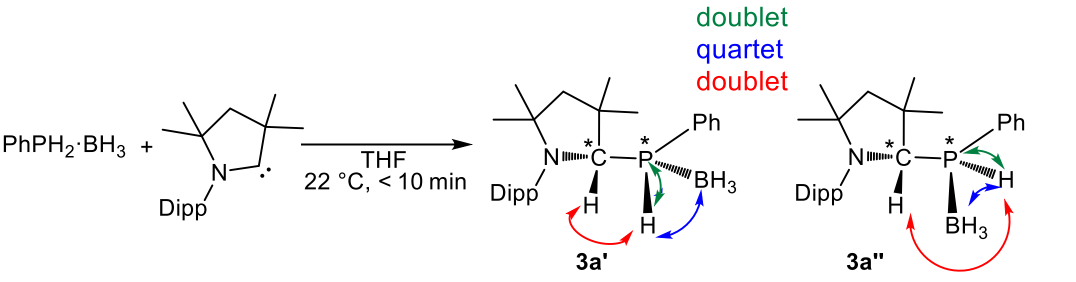

* = chiral centre

$PhPH_2{\cdot}BH_3$ (74 mg, 0.60 mmol) and CAAC^{Me} (171 mg, 0.60 mmol) were dissolved in THF (0.5 mL) in a quartz J. Young NMR tube. P–H activation occurred instantly at 22 °C to give two diastereomers. This was immediately followed by a degree of formation of $[PhHPBH_2]_n$ and (CAAC^{Me})H_2, hence isolation of these compounds has not been achieved and there are traces of (CAAC^{Me})H_2 visible in the ^{1}H NMR spectrum. The initial ratio of diastereomers observed after ten minutes in solution was 1:12.4.

Method B:

3a can also be synthesised through a stepwise procedure

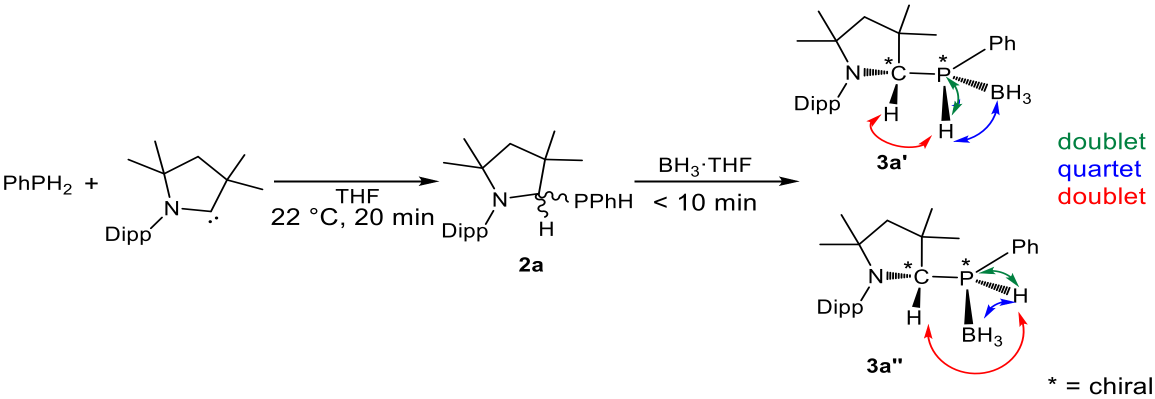

* = chiral centre

$PPhH_2$ (used as a ca. 10 % weight solution in hexanes, 4.24 g of hexanes solution, 3.90 mmol) was added to a solution of CAAC^{Me} (1.00 g, 3.50 mmol) in THF (10 mL) and stirred for 20 minutes at 22 °C. The volatiles were removed *in vacuo* to leave a pale yellow powder. NMR

spectroscopic data for CAAC^{Me}(H)(PPhH) obtained was directly comparable to those described in the literature for CAAC^{Cy}(H)(PPhH).[7] The powder was redissolved in THF (5 mL) and BH_3·THF (3.50 mL of a 1M in THF solution, 3.50 mmol) was added. Volatiles were immediately removed *in vacuo*, however as with method A above it was not possible to isolate the product as a degree of formation of $[PhHPBH_2]_n$ and (CAAC^{Me})H_2 immediately occurs.

Method C:

3a was also independently synthesised through a salt metathesis reaction

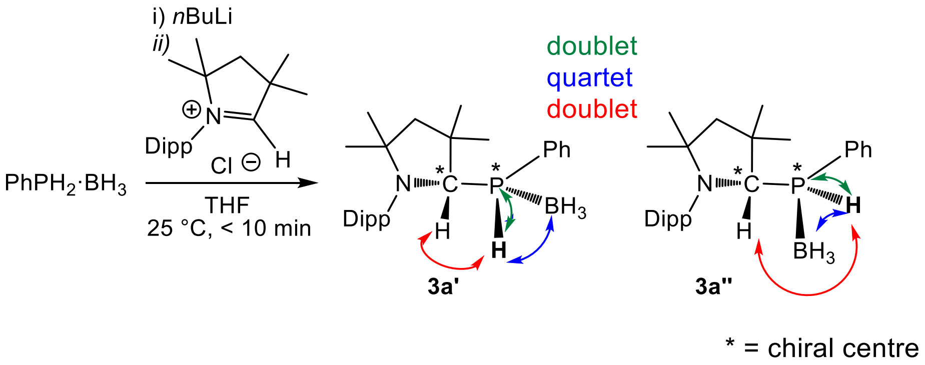

*n*BuLi (56 µL of a 1.6 M in hexanes solution, 0.09 mmol) was added to a solution of $PhPH_2·BH_3$ (10 mg, 0.09 mmol) in THF (0.5 mL) in a quartz J. Young NMR tube and at 22 °C and after 10 min. [CAAC^{Me}H]Cl (30 mg, 0.09 mmol) was added. Immediate conversion to the product was observed and the identity confirmed using ^{1}H, ^{11}B and ^{31}P NMR spectroscopy, however as above it was not possible to isolate the product as the formation of $[PhHPBH_2]_n$ and (CAAC^{Me})H_2 immediately occurs.

^{1}H NMR (400 MHz, 25 °C, THF-d_8): δ = 7.70-7.38 (m, 5H, Ar), 7.26-7.20 (m, 3H, Ar^{Dipp}), 4.84 (dqd, $^1J_{PH}$ = 364 Hz, $^3J_{HH}$ = 7.2 Hz and $^3J_{HH}$ = 2.5 Hz, 1H, P*H*), 4.42 (dd, $^2J_{HP}$ = 4.2 Hz, $^3J_{HH}$ = 2.5 Hz, 1H, NC*H*), 4.11 (sept, $^3J_{HH}$ = 6.6 Hz, 1H, C*H*(CH_3)$_2$), δ = 3.08 (sept, $^3J_{HH}$ = 6.6 Hz, 1H, C*H*(CH_3)$_2$), 2.05 (ABq, 1H, CH_2), 1.64 (s, 3H, NC(CH_3)$_2$), 1.58 (s, 3H, NC(CH_3)$_2$), 1.37 (d, $^3J_{HH}$ = 6.6 Hz, 3H, CH(CH_3)$_2$), 1.29 (d, $^3J_{HH}$ = 6.6 Hz, 3H, CH(CH_3)$_2$), 1.24 (d, $^3J_{HH}$ = 6.6 Hz, 3H, CH(CH_3)$_2$), 1.20 (d, $^3J_{HH}$ = 6.6 Hz, 3H, CH(CH_3)$_2$), 0.95 (s, 3H, C(CH_3)$_2$), 0.84 (s, 3H, C(CH_3)$_2$). Data given only for the major diastereomer, **3a'**.

^{11}B NMR (96 MHz, 22 °C THF-d_8): δ = -40.1 (m, br).

No detection of separate peaks for the different isomers, postulated to be due to broad peaks overlapping.

^{31}P NMR (122 MHz, 22 °C THF-d_8): δ = -4.1 (d, $^1J_{PH}$ = 364 Hz) (**3a''**), -12.4 (d, $^1J_{PH}$ = 364 Hz) (**3a'**).

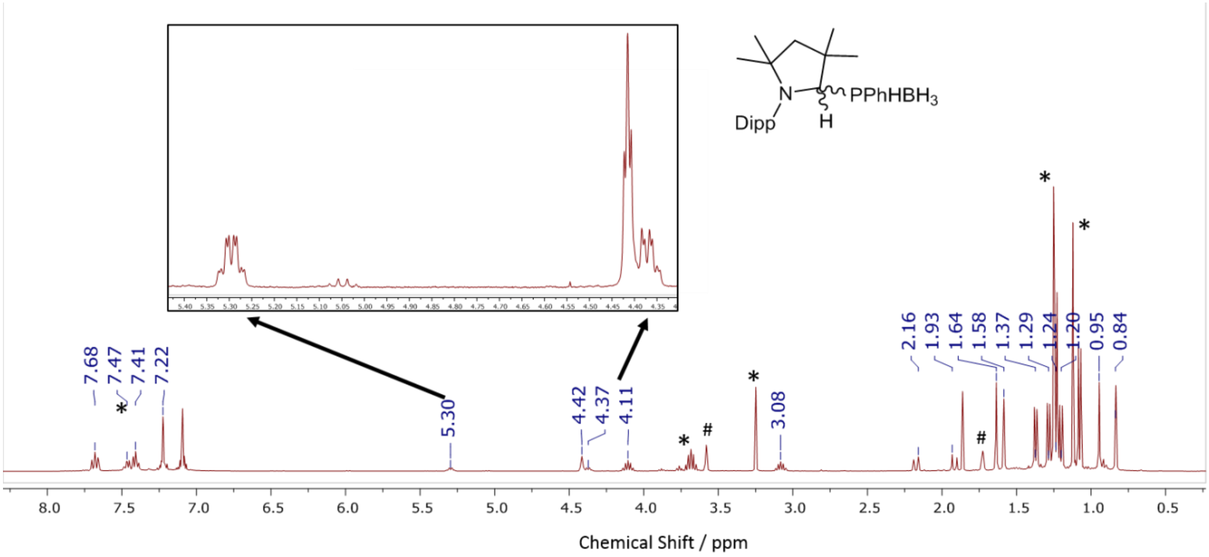

Supplementary Figure 8. ^{1}H NMR spectrum (400 MHz, 25 °C, THF-d_8) of **3a** (* denotes (CAAC^{Me})H_2, # denotes residual partially protiated THF). Doublet of quartets of doublets splitting pattern of P–H proton is expanded.

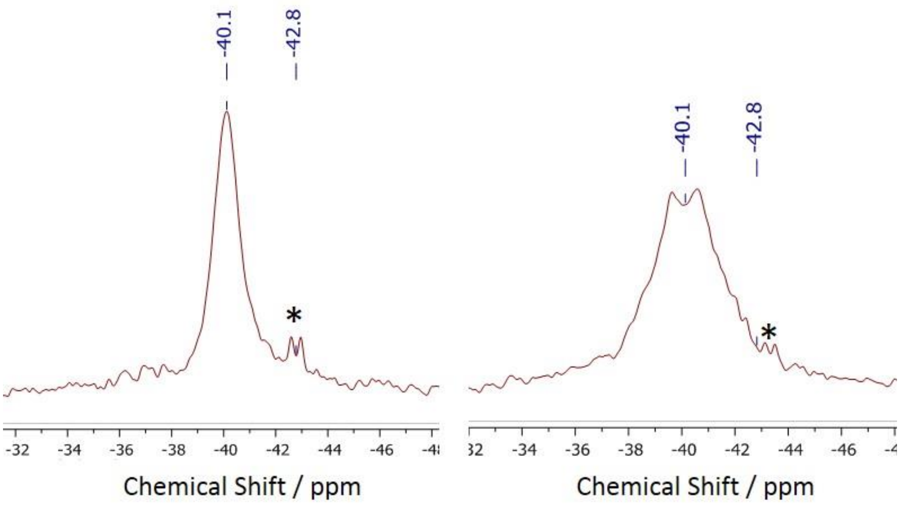

Supplementary Figure 9. ^{11}B{^{1}H} (left) and ^{11}B (right) NMR spectra (96 MHz, 22 °C, THF-d_8) of **3a** (*denotes trace (CAAC^{Me})·BH_3 adduct).[7]

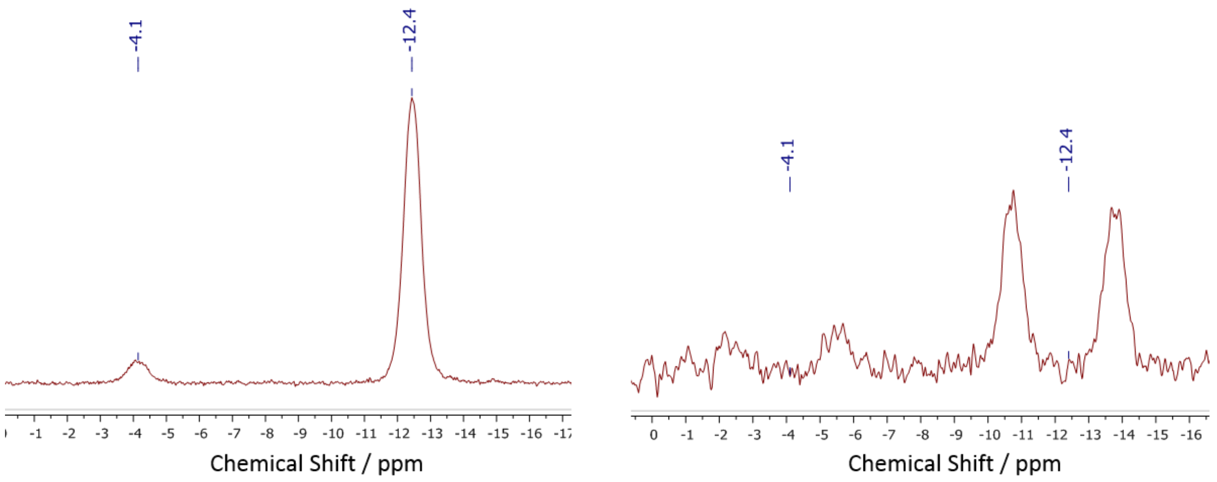

Supplementary Figure 10. $^{31}P\{^{1}H\}$ (left) and ^{31}P (right) NMR spectra (122 MHz, 22 °C, THF-d_8) of **3a**.

General procedure for dehydropolymerisation of $PhPH_2{\cdot}BH_3$ using CAAC^{Me}

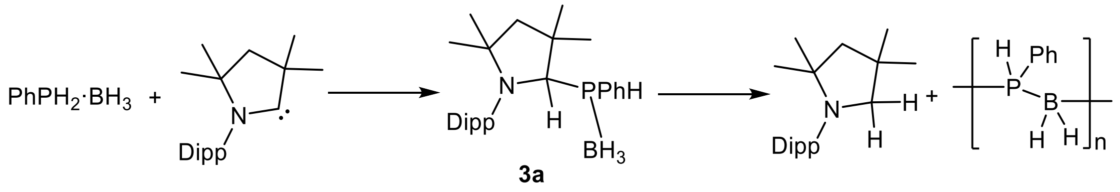

$PhPH_2{\cdot}BH_3$ and CAAC^{Me} were dissolved in THF or toluene in the quantities shown in Supplementary Table 1 and the reaction performed under the conditions shown in either a J. Young NMR tube or a J. Young Schlenk tube. Mass spectroscopy and GPC analysis were carried out after precipitation of the reaction mixture into cold (−40 °C), stirring hexanes. More detailed information on run 4 is shown below.

Supplementary Table 1. Influence of temperature, solvent and concentration on the formation of polyphenylphosphinoborane, $[PhHPBH_2]_n$ in a closed system.

Run	Temp. (°C)	Solvent	Volume (mL)	$CAAC^{Me}$ (mg)	$PhPH_2{\cdot}BH_3$ (mg)	Time (h)[a]	DP[b]	M_n (Da)[c]	PDI[c]
1	22	THF	0.5	71	31	120	205	25,000	1.55
2	60	THF	0.5	14	6	3	[d]	[d]	[d]
3	60	THF	0.5	71	31	3	410	50,100	1.27
4	60	THF	1.0	360	156	3	686	83,800	1.13
5	60	toluene	0.5	71	31	3	290	35,400	1.28
6	110	toluene	0.5	71	31	0.5	230	28,000	1.52
7	110	none	n/a	142	62	3	302	36,800	1.39

[a]time taken for full conversion by ^{31}P NMR spectroscopy [b] degree of polymerisation measured by GPC [c]measured using GPC analysis [d]no high molecular weight material recovered after precipitation.

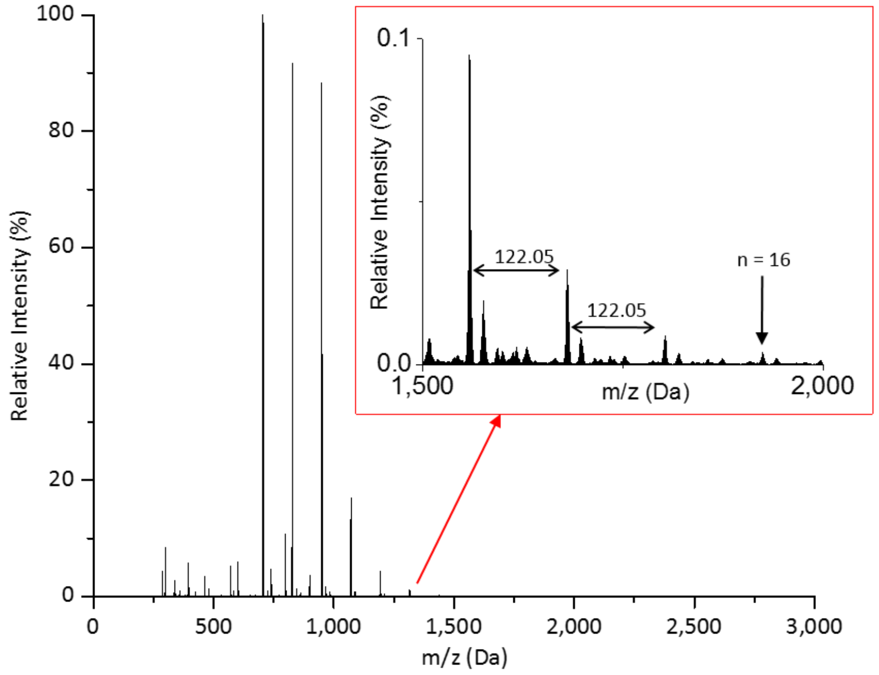

Supplementary Figure 11. ESI(+)-MS spectrum of $[PhHPBH_2]_n$ product from run 1 of Supplementary Table 1 in DCM.

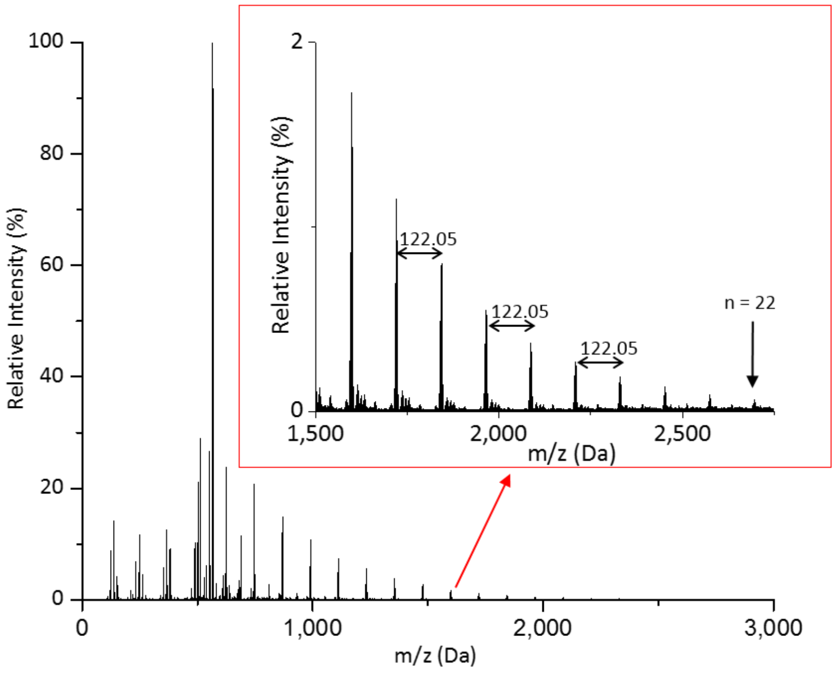

Supplementary Figure 12. ESI(+)-MS spectrum of $[PhHPBH_2]_n$ product from run 2 of Supplementary Table 1 in DCM.

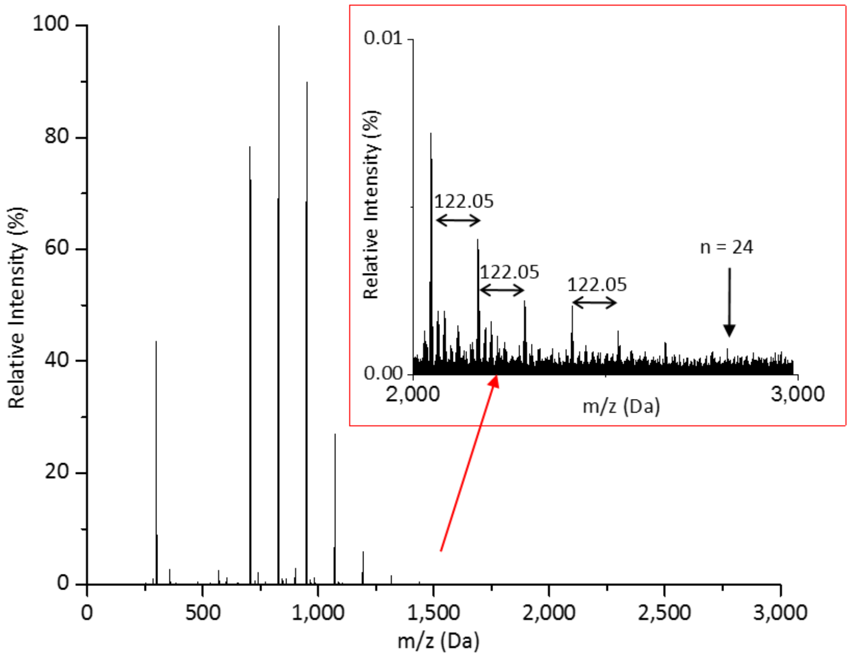

Supplementary Figure 13. ESI(-)-MS spectrum of $[PhHPBH_2]_n$ product from run 3 of Supplementary Table 1 in DCM.

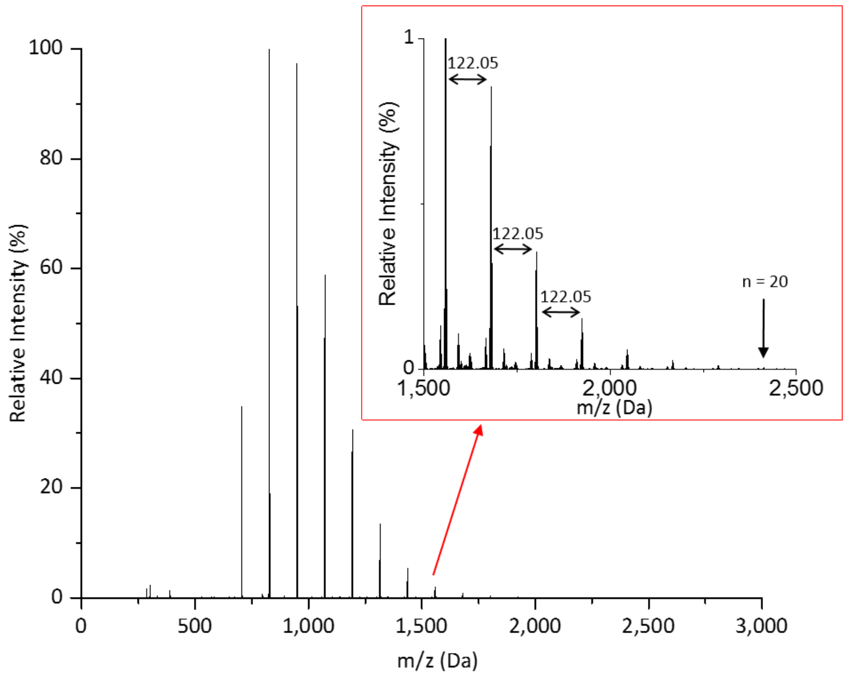

Supplementary Figure 14. ESI(+)-MS spectrum of $[PhHPBH_2]_n$ product from run 5 of Supplementary Table 1 in DCM.

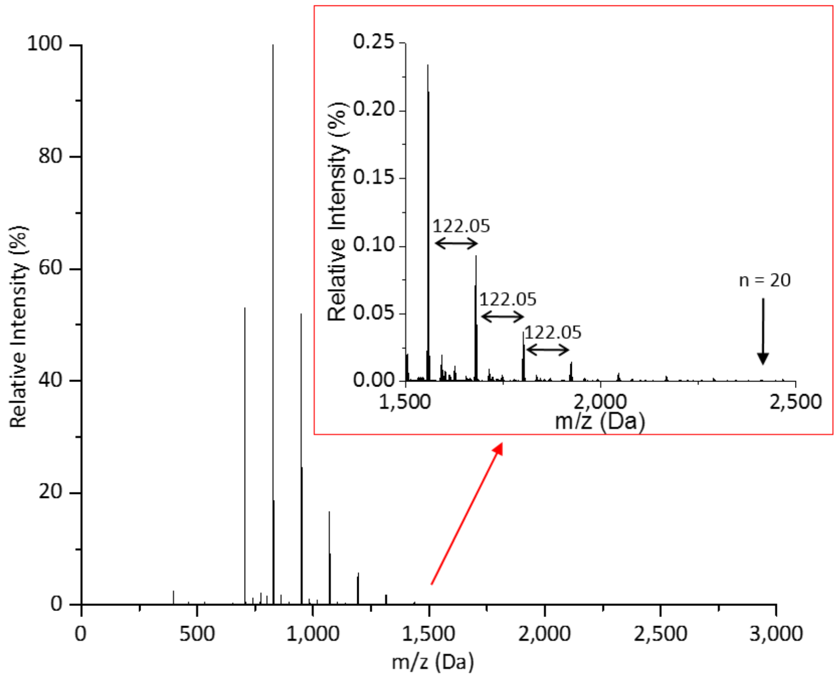

Supplementary Figure 15. ESI(+)-MS spectrum of $[PhHPBH_2]_n$ product from run 6 of Supplementary Table 1 in DCM.

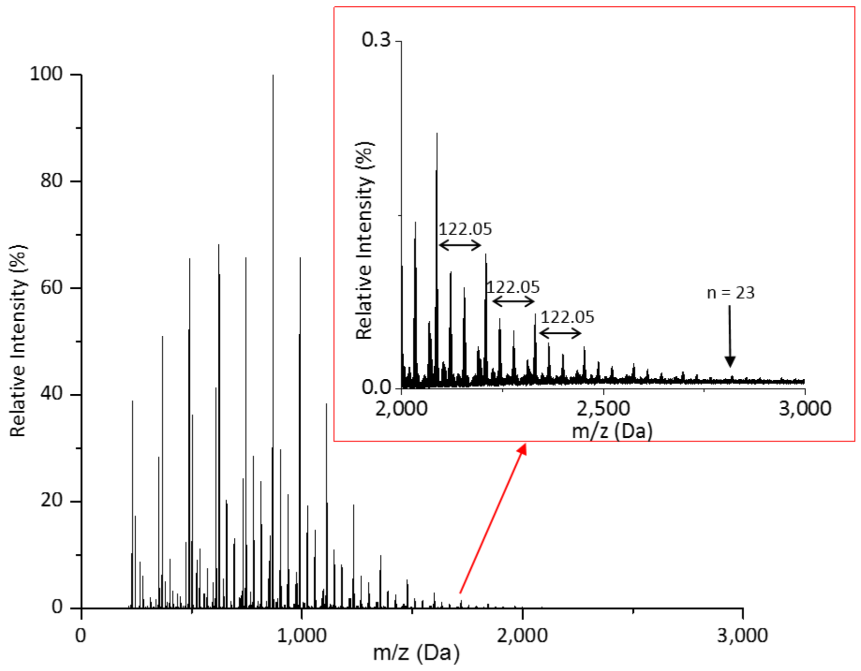

Supplementary Figure 16. ESI(+)-MS spectrum of $[PhHPBH_2]_n$ product from run 7 of Supplementary Table 1 in DCM.

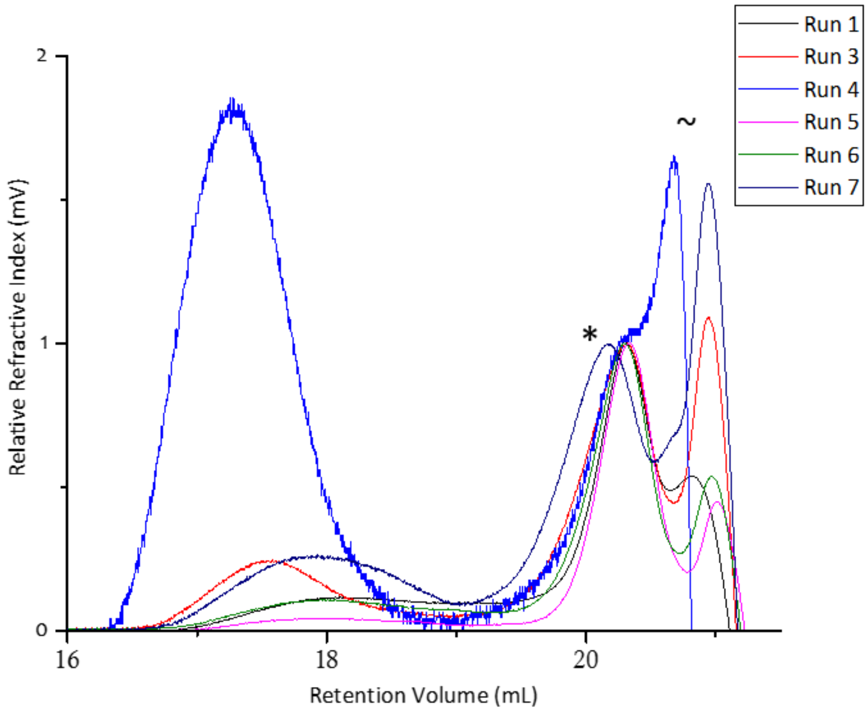

Supplementary Figure 17. GPC chromatogram of precipitated $[PhPHBH_2]_n$ products from for runs 1-7 from Supplementary Table 1. 2 mg mL^{-1} in THF with 0.1 w/w % $n$$Bu_4$NBr in the THF eluent (*oligomeric material, ~system peak).

More detailed procedure for run 4

$PhPH_2·BH_3$ (156 mg, 1.26 mmol) and CAAC^{Me} (360 mg, 1.26 mmol) were dissolved in THF (1 mL) in a J. Young Schlenk tube, sealed, and the reaction mixture was stirred at 60 °C for three hours. The reaction mixture was added dropwise into 20 mL of rapidly stirred cold hexanes at -40 °C yielding a precipitate and the supernatant was decanted. The precipitation was repeated twice more prior to drying in vacuo to leave a white powder of the $[PhHPBH_2]_n$ polymer product. Yield (precipitated material) = 42 mg (27 %). GPC (2 mg mL^{-1}): M_n = 83,800 Da; PDI = 1.17.

^{1}H NMR (400 MHz, 25 °C, THF-d_8): δ = 7.38-7.03 (m, 5H, Ar), 4.32 (d, $^1J_{PH}$ = 370 Hz, 1H, PH), 1.86-1.20 (m, 2H, BH).

^{11}B NMR (96 MHz, 21 °C, THF-d_8): δ = -34.6 (m, br).

^{31}P NMR (122 MHz, 21 °C, THF-d_8): δ = -48.4 (d, $^1J_{PH}$ = 348 Hz).

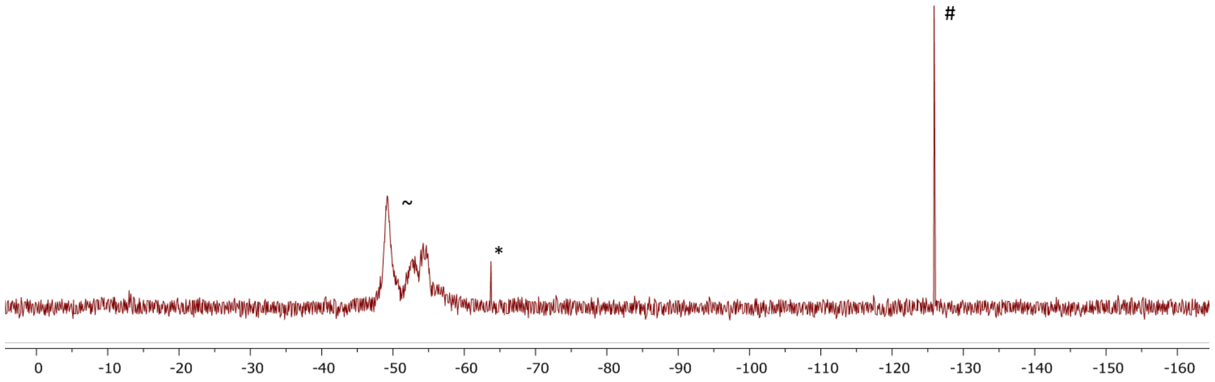

Supplementary Figure 18. ^{31}P{^{1}H} NMR spectra (122 MHz, 25 °C, THF-d_8) of the crude reaction mixture between $PhPH_2·BH_3$ and CAAC^{Me} for run 4 (~ denotes material which may be a mixture of linear and cyclic oligomers[8] and polymeric $[PhHPBH_2]_n$, * denotes **2a**, # denotes $PhPH_2$).

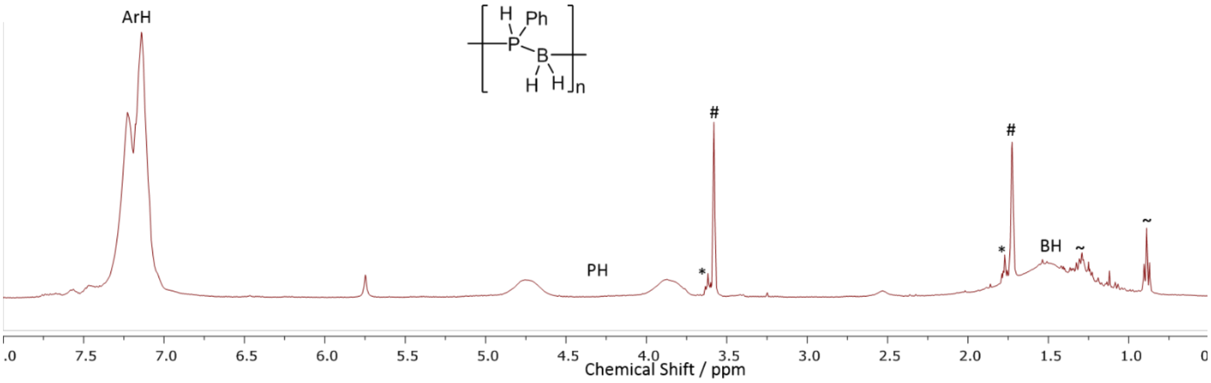

Supplementary Figure 19. ^{1}H NMR spectrum (400 MHz, 25 °C, THF-d_8) of isolated $[PhPHBH_2]_n$ from run 4 (* denotes partially protiated THF, # denotes THF, ~ denotes hexanes).

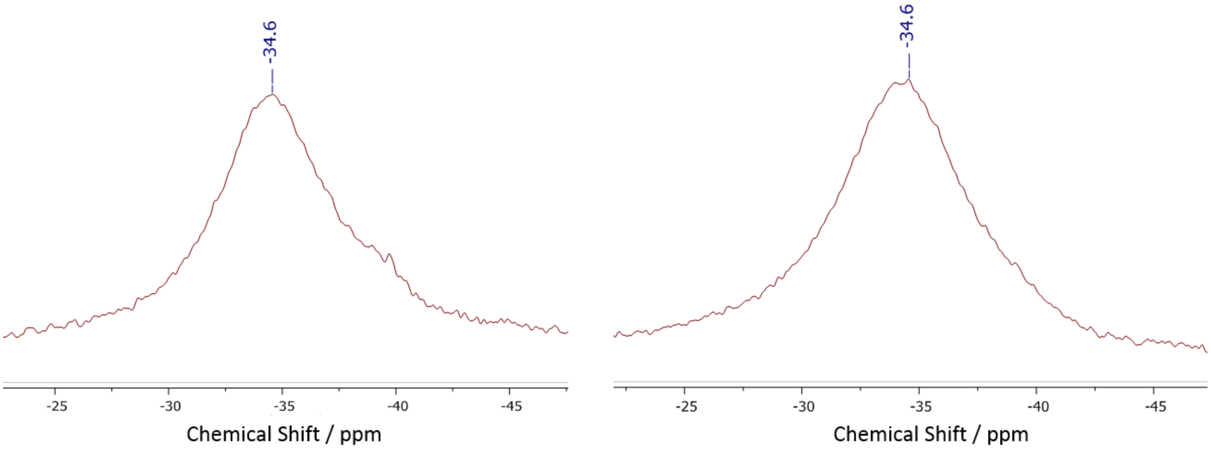

Supplementary Figure 20. ^{11}B{^{1}H} (left) and ^{11}B (right) NMR spectra (96 MHz, 22 °C, THF-d_8) of isolated $[PhPHBH_2]_n$ from run 4.

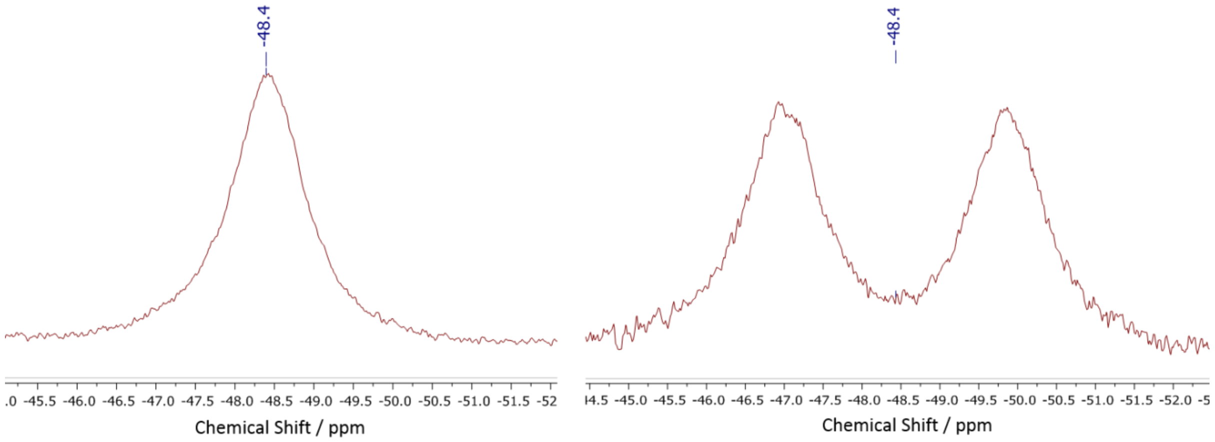

Supplementary Figure 21. $^{31}P\{^{1}H\}$ (left) and ^{31}P (right) NMR spectra (122 MHz, 25 °C, THF-d_8) of isolated $[PhPHBH_2]_n$ from run 4.

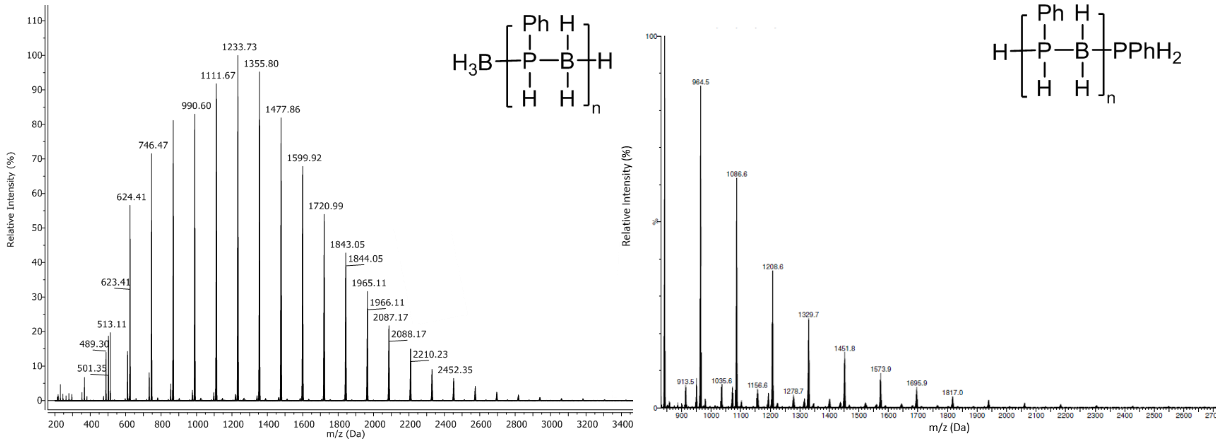

Supplementary Figure 22. ESI(-)-MS (left) and ESI(+)-MS (right) spectra of $[PhPHBH_2]_n$ product from run 4 in Supplementary Table 1. The predominant species are linear systems with a BH_3 end group $(BH_3\text{-}[PhPHBH_2]_n\text{-}H)^-$ or a $PPhH_2$ end group $(H\text{-}[PhPHBH_2]_n\text{-}PPhH)^+$ respectively.

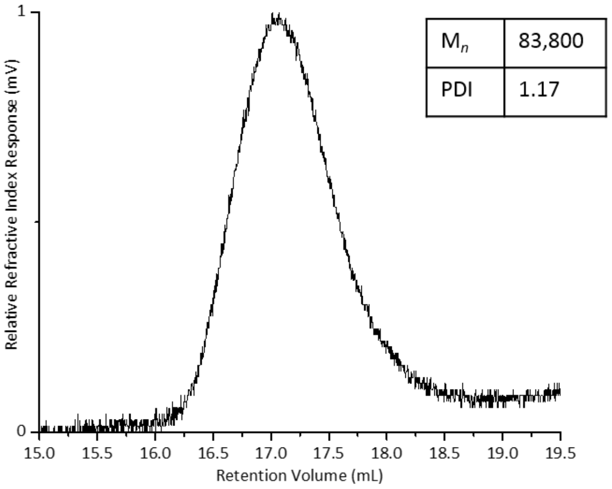

Supplementary Figure 23. GPC chromatogram of precipitated $[PhPHBH_2]_n$ product from run 4 of Supplementary Table 1 (2 mg mL^{-1} in THF with 0.1 w/w % nBu$_4$NBr in the THF eluent).

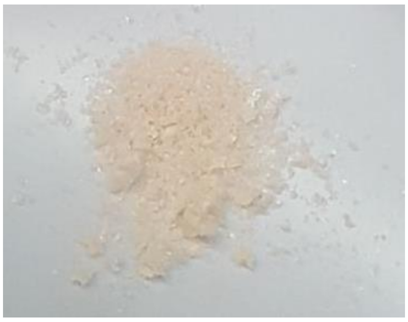

Supplementary Figure 24. Photograph of isolated $[PhPHBH_2]_n$. Photograph taken by N. L. Oldroyd.

Synthesis of (CAAC^{Me})H_2

(CAAC^{Me})H_2 was obtained by removing the solvent in vacuo from the filtrate post precipitation of the polymer in run 4. X-ray diffraction quality crystals were obtained through sublimation (22 °C, 5 x 10^{-2} Pa).

^{1}H NMR (400 MHz, 25 °C, C_6D_6): δ = 7.24 (m, 1H, Ar^p), 7.32 (d, 2H, Ar^m), 3.72 (sept, $^3J_{HH}$ = 6.7 Hz, 2H, CH(CH_3)$_2$), 3.34 (s, 2H, NCH_2), 1.97 (s, 2H, NC(CH_3)$_2$CH_2), 1.38 (d, $^3J_{HH}$ = 6.7 Hz, 6H, CH(CH_3)$_2$), 1.37 (s, 6H, NC(CH_3)$_2$), 1.25 (s, 6H, C(CH_3)$_2$), 1.23 (d, $^3J_{HH}$ = 6.7 Hz, 6H, CH(CH_3)$_2$).

^{13}C NMR (101 MHz, 25 °C, C_6D_6): δ = 152.4 (Ar^i), 139.1 (Ar^o), 126.5 (Ar^m), 123.9 (Ar^p), 67.7 (NCH$_2$), δ = 63.3 (NC(CH_3)$_2$), 56.8 (NC(CH_3)$_2$(CH$_2$), 37.3 (NC(CH$_3$)$_2$), 30.4 (N(CH_2)C(CH$_3$)$_2$), 29.0 (CH(CH_3)$_2$), 28.4 (NC(CH$_3$)$_2$), 26.7 (CH(CH$_3$)$_2$), 23.2 (CH(CH$_3$)$_2$).

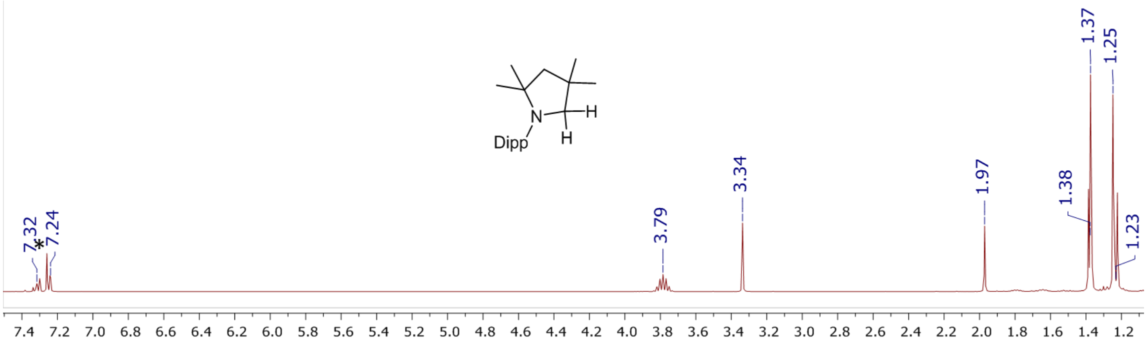

Supplementary Figure 25. ^{1}H NMR spectrum (400 MHz, 25 °C, C_6D_6) of (CAAC^{Me})H_2 (*denotes residual partially protiated benzene).

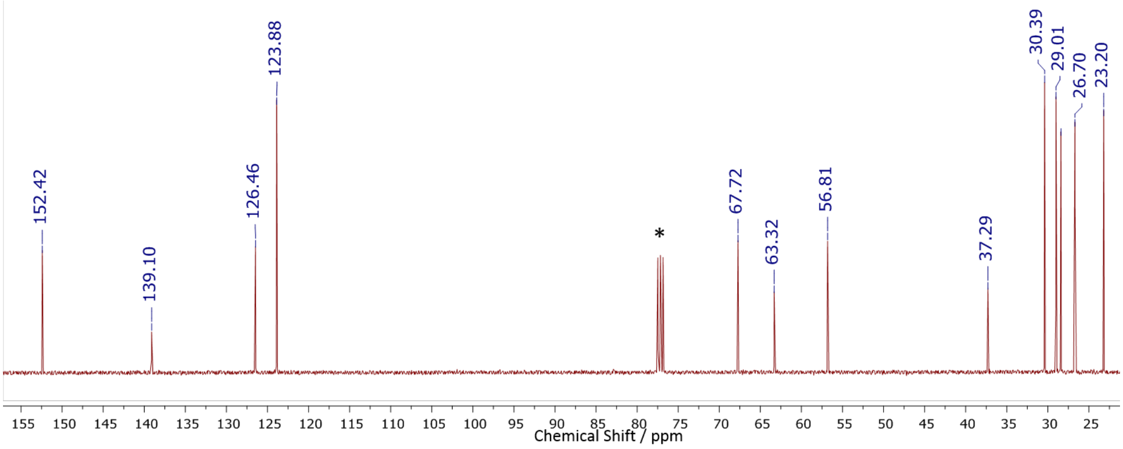

Supplementary Figure 26. ^{13}C NMR spectrum (101 MHz, 25 °C, C_6D_6) of (CAAC^{Me})H_2 (*denotes benzene).

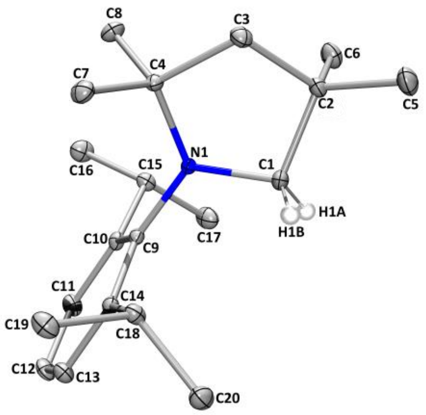

Supplementary Figure 27. Thermal ellipsoid plot of (CAAC^{Me})H_2. Ellipsoids are shown at the 30% probability level. H atoms other than those at the C1 centre have been omitted for clarity.

Mechanistic studies

Proposed and subsequently discounted mechanisms for phosphine-borane dehydrogenation mediated by CAAC^{Me}

i) P–H activation followed by σ-bond metathesis

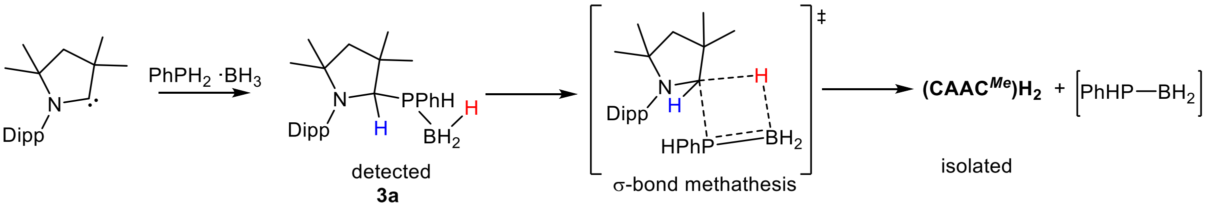

P–H activation to give **3a** followed by a σ-bond metathesis step, where simultaneous B–H and C–P bond cleavage occurs, releasing the reactive monomer, [PhHP-BH_2]. A similar hydride transfer mechanism has been reported previously for the conversion of an N-heterocyclic phosphinophosphine-borane to a mixture of N-heterocyclic phosphine and cyclic phosphinoboranes.[9] This mechanism was ruled out using DFT as no concerted transition state leading to (CAAC^{Me})H_2 and [PhHP-BH_2] could be located.

ii) P–H activation followed by H_2 elimination

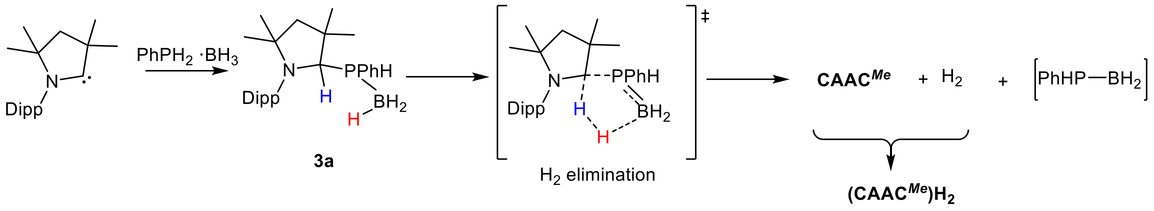

P–H activation to give **3a** followed by the formation of a five-centre transition state where the B–H hydride and C–H proton combine to eliminate H_2 together with [PhH-BH$_2$] and CAAC^{Me} which subsequently reacts with H_2 to give (CAAC^{Me})H_2. We were able to experimentally rule out this mechanistic proposal based on control experiments that showed CAAC^{Me} does not react with H_2 (0.2 M, 4 atm of H_2, C_6D_6, 60 °C, 24 h) (See 'Reaction of CAAC^{Me} with H_2 at ca. 4atm' below).

iii) Concerted hydrogen transfer

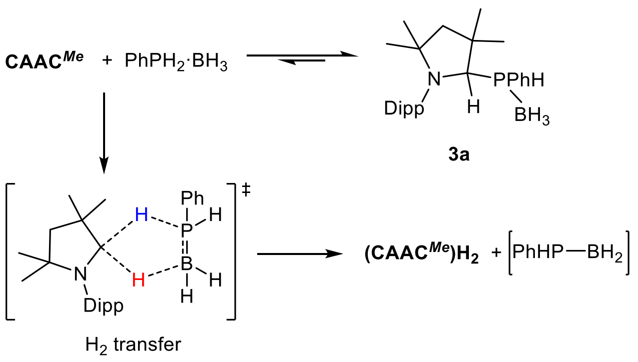

The P–H activation to give **3a** is reversible and there is an irreversible concerted hydrogen transfer from $PhPH_2{\cdot}BH_3$ to CAAC^{Me} to give (CAAC^{Me})H_2 and [PhHP-BH$_2$]. This mechanism was ruled out using DFT as no concerted transition state (TS) leading to (CAAC^{Me})H_2 and [PhHP-BH$_2$] in one step could be located.

iv) B–H activation followed by σ-bond metathesis

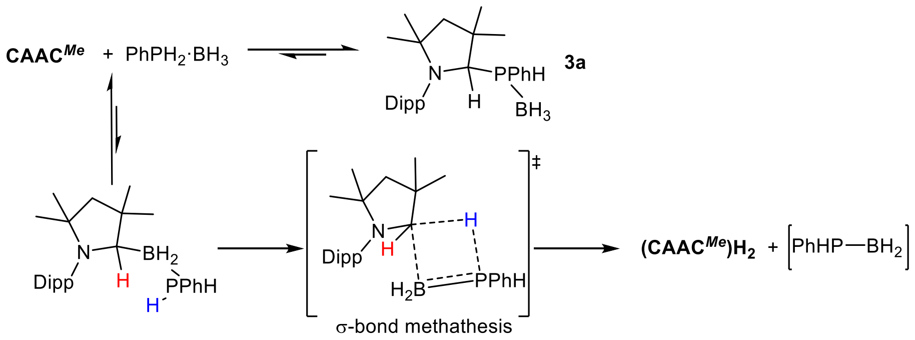

The P–H activation to give **3a** is reversible and the products are achieved via a B–H activation compound and undergoing σ-bond metathesis step where simultaneous P–H and C–B bond cleavage occurs. This mechanism was ruled out using DFT as the TS for the B–H activation step has a significantly higher energy barrier than for the P–H activation step (34.2 kcal mol^{-1} vs 4.9 kcal mol^{-1}).

v) Homolytic cleavage of P–C bond

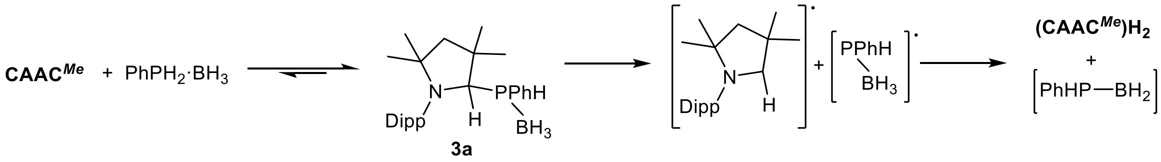

After **3a** is formed homolytic cleavage of the P–C bond occurs to give a $[CAAC^{Me}(H)]^{\bullet}$ and a $[PhPH(BH_3)]^{\bullet}$ radical. The mechanism was ruled out using DFT as the energy for P–C bond homolysis is very high (55.8 kcal mol^{-1}) and significantly higher than the heterolytic cleavage of the P–C bond (35.3 kcal mol^{-1})

vi) Intermolecular hydride abstraction with an iminium ion leading to a borenium ion

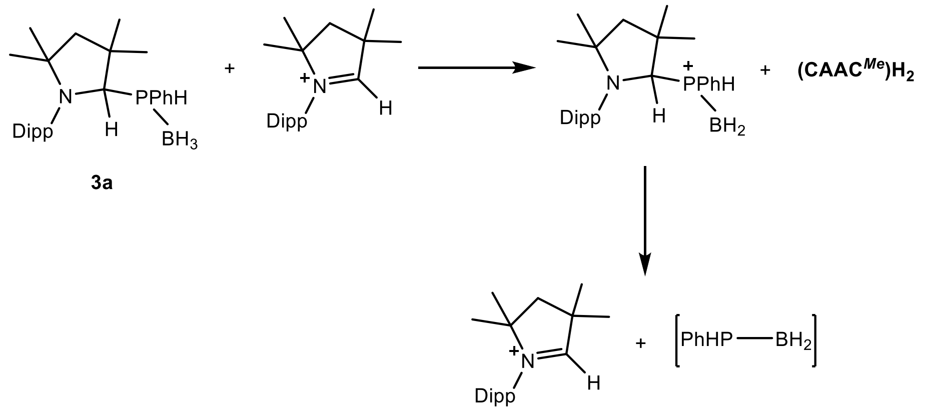

We considered an intermolecular hydride abstraction from **3a**, using the [CAAC(H)]$^+$ iminum ion, to give (CAAC^{Me})H_2 and a phosphinoborenium ion, which is subsequently converted to [CAAC(H)]$^+$ and [PhHP-BH_2] via P–C cleavage. The intermolecular hydride abstraction pathway requires the presence of an iminium ion which is formed by P–C bond dissociation of **3a** as discussed in the main text. According to DFT studies the intermolecular hydride abstraction step is energetically unlikely as the barrier for hydride abstraction from [PhHP–BH_3]$^-$ via **TS5** (5.3 kcal mol^{-1}) (Supplementary Figure 31) is considerably lower than the energy associated with the hydride abstraction from **3a** to give the phosphinoborenium intermediate (> 18 kcal mol^{-1}). Experimentally it was also found that the addition of [CAAC^{Me}H]Cl did not accelerate the reaction rate.

<u>Supplementary discussion of the polymerisation mechanism from phosphinoborane monomers</u>

Studies on the mechanism of polymerisation of phosphinoborane monomers, [RR'P-BH_2], generated from the reaction of a CAAC with a phosphine-borane substrate, are complicated due to several key factors:

1) The monomers generated *in situ* are highly reactive and readily polymerise and thus have not been isolated in their pure monomeric form.

2) A number of different polymer architectures are obtained from the polymerisation reaction including cyclic and linear oligomers, and linear polymers.

3) There is not a single well-defined initiator-derived end-group, and there are limitations in determining the identity of the various end-groups present in samples of the polymers based on the experimental techniques available.

Here we propose a polymerisation mechanism which involves several non-mutually exclusive pathways which likely occur in parallel, and discuss our experimental observations and limitations.

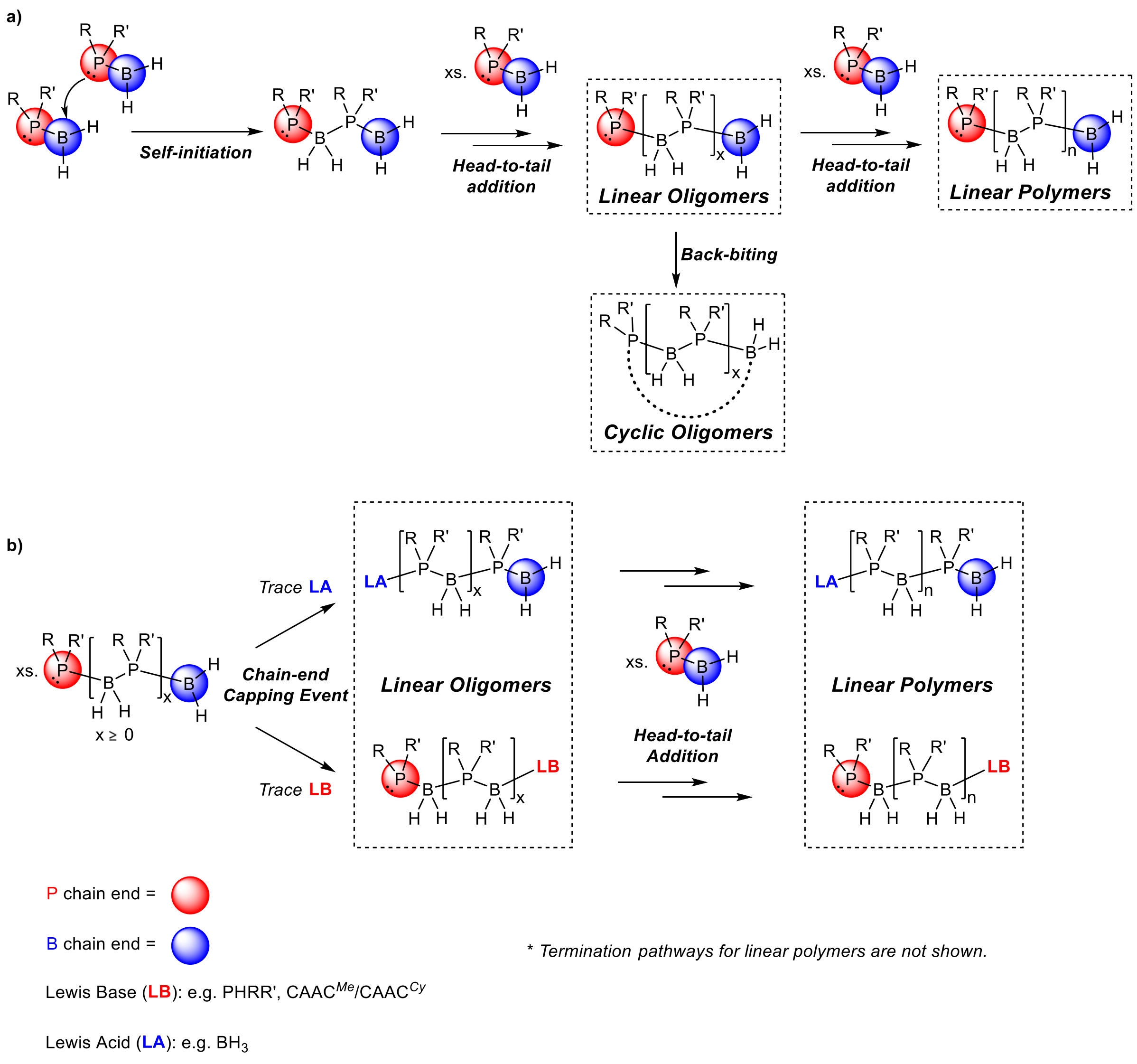

We propose that the ambipolar phosphinoborane monomer can self-initiate and spontaneously undergo an addition head-to-tail polymerisation sequence, see pathway a) shown above. This mechanism is reminiscent of the metal-free thermolysis of amine-stabilised phosphinoboranes reported by Scheer and Manners.[10] Given the polar structure of the

monomers and the fact that B–B and P–P linkages are not observable in polymer samples by either ^{11}B or ^{31}P NMR spectroscopy it is likely that head-to-head and tail-to-tail propagation are not major pathways. It is possible to obtain both cyclic oligomers and linear polymers via a self-initiated route. Cyclic oligomers likely form through back-biting of the phosphorus chain end with the boron chain end. Linear polymers are obtained when the rate of linear chain propagation is greater than the rate of back-biting; these rates are often competitive and highly dependent on the nature of the substituents and the degree of substitution on the phosphinoborane monomer. For example, polymerisation of [$PhHP-BH_2$] favours mostly linear polymer, while for the *P*-disubstituted [Ph_2P-BH_2] a significant quantity of cyclic oligomers are obtained as determined by NMR spectroscopy in the crude polymeric material (Supplementary Figure 40). The milder conditions required to generate the phosphinoborane using CAAC likely do not facilitate branching or cross-linking that could inhibit high molar mass material being formed as opposed to when the monomer is thermally generated from the phosphine-borane under more harsh thermal conditions.[8]

In addition, in concert with the proposed self-initiated pathway a) above, trace Lewis acids or bases present in the reaction mixture could engage in chain-end capping of polymer chain or monomer at any point during the polymerisation. These chain-end capping reactions may favour linear propagation by preventing the back-biting reaction, see pathway b) above. Traces of free phosphine and borane can be generated thermally from dissociation of the phosphine-borane adduct, and traces of free CAAC can also be present in the reaction mixture. It is noteworthy that the electronic effect of the neutral Lewis acid or base capping group on the reactive P or B chain-end diminishes as the polymer chain lengthens, this is in contrast to radical or ionic chain polymerisations. In well-defined chain polymerisations the radical or charged reactive site consistently migrates to the end of polymer chain with each monomer addition, leading to solely unidirectional chain growth. The reactivity of the P or B chain-end towards the successive addition of further monomer or oligomers is expected to be approximately equivalent for both Lewis acid or base capped polymer chains with a high degree of polymerisation and for completely uncapped (oligo)polymer chains capable of bidirectional growth.

Some experimental evidence for chain termination involving chain-end capping with Lewis acids and bases are the detection of phosphine- and borane-based end groups for $[PhHPBH_2]_n$ (Supplementary Figure 22), and CAAC-based end groups for $[Ph_2PBH_2]_n$ and $[PhEtPBH_2]_n$ (see Supplementary Figs. 36, 37, 44, 52 and 56) using ESI-MS. The reaction of 2 equiv. of $CAAC^{Me}$ with $PhPH_2{\cdot}BH_3$ at an initial concentration of 1.26 M in THF at 60 °C for 3h (see Table 1, run 4, for analogous conditions with one equivalent of $CAAC^{Me}$) resulted in primarily oligomeric material below the calibration limit of the GPC (M_n < ca. 2,300 Da) and only trace quantities of higher mass polymer. The excess of $CAAC^{Me}$ likely results in a significant degree of chain-end capping reactions leading to reduced molecular masses for the resulting material.

The polymerisation mechanism may involve a combination of both chain growth and step growth processes. Another possibility during the polymerisation reaction that may be occurring to an extent in either of the pathways shown above is the head-to-tail addition of oligomers which possess reactive B and/or P chain ends onto existing polymer chains with reactive termini.[11]

There are some experimental limitations of ESI-MS as a technique, in particular for the identification of cyclic species and polymer end-groups. The lack of evidence for cyclic species in the ESI-MS data could be a feature of the ionisation method rather than the sample. ESI-MS only detects charged species and the ionisation of cyclic species is inherently difficult given the lack of available lone pairs or vacant orbitals. In comparison, depending on the end-groups, linear species can readily be ionised and are expected to be over-represented in the ESI-MS spectra. ESI-MS is also only detecting the oligomeric fraction of the material that is present (below ca. 3,000 Da) and it is possible that the bulk of the high molar mass material could have different end groups. In addition, the ionisation conditions within the ESI-MS instrument may be altering the end groups actually present in the polymer sample.

Reaction of $CAAC^{Me}$ with H_2 at ca. 4 atm

$CAAC^{Me}$ (86 mg, 0.30 mmol) was dissolved in C_6D_6 (1.50 mL) in a vial and 0.50 mL of this solution was transferred to a J. Young NMR tube. The solution was degassed through freeze-pump-thaw cycles prior to adding H_2 (1 atm) whilst chilling at -196 °C using liquid nitrogen. Upon thawing no dihydrogen activation was observed using ^1H NMR either at 22 °C or upon heating to 60 °C for 24 hours.

Kinetics measurements to probe reaction mechanism

$PhPH_2{\cdot}BH_3$ and $CAAC^{Me}$, in the quantities shown in Supplementary Table 2 were dissolved in THF (0.5 mL) in a quartz J. Young NMR tube containing a capillary of neat PCl_3 as an internal standard. The peak corresponding to **3a''** (δ_P = -12.4 ppm) was integrated relative to the capillary over at least two half lives in each case. The kinetic studies were all performed solely on the **3a''** disatereomer as **3a'** is consumed much more rapidly.

Supplementary Table 2. Conditions for kinetics experiments.

Run	Conc. (M)	$CAAC^{Me}$ (mg)	$PhPH_2{\cdot}BH_3$ (mg)	Temp. (°C)	k	$T^{1/2}$ (h)
8	0.50	71	31	22	7.87 x 10^{-6}	24.5
9	0.50	71	31	30	1.64 x 10^{-5}	11.7
10	0.50	71	31	40	5.56 x 10^{-5}	3.5
11	0.50	71	31	50	1.42 x 10^{-4}	1.4
12	0.50	71	31	60	4.33 x 10^{-4}	0.5
13	0.30	43	19	50	1.39 x 10^{-4}	1.4
14	0.70	100	43	50	1.24 x 10^{-4}	1.6
15[a]	0.50	71	31	50	8.00 x 10^{-5}	2.4

[a]toluene used as solvent

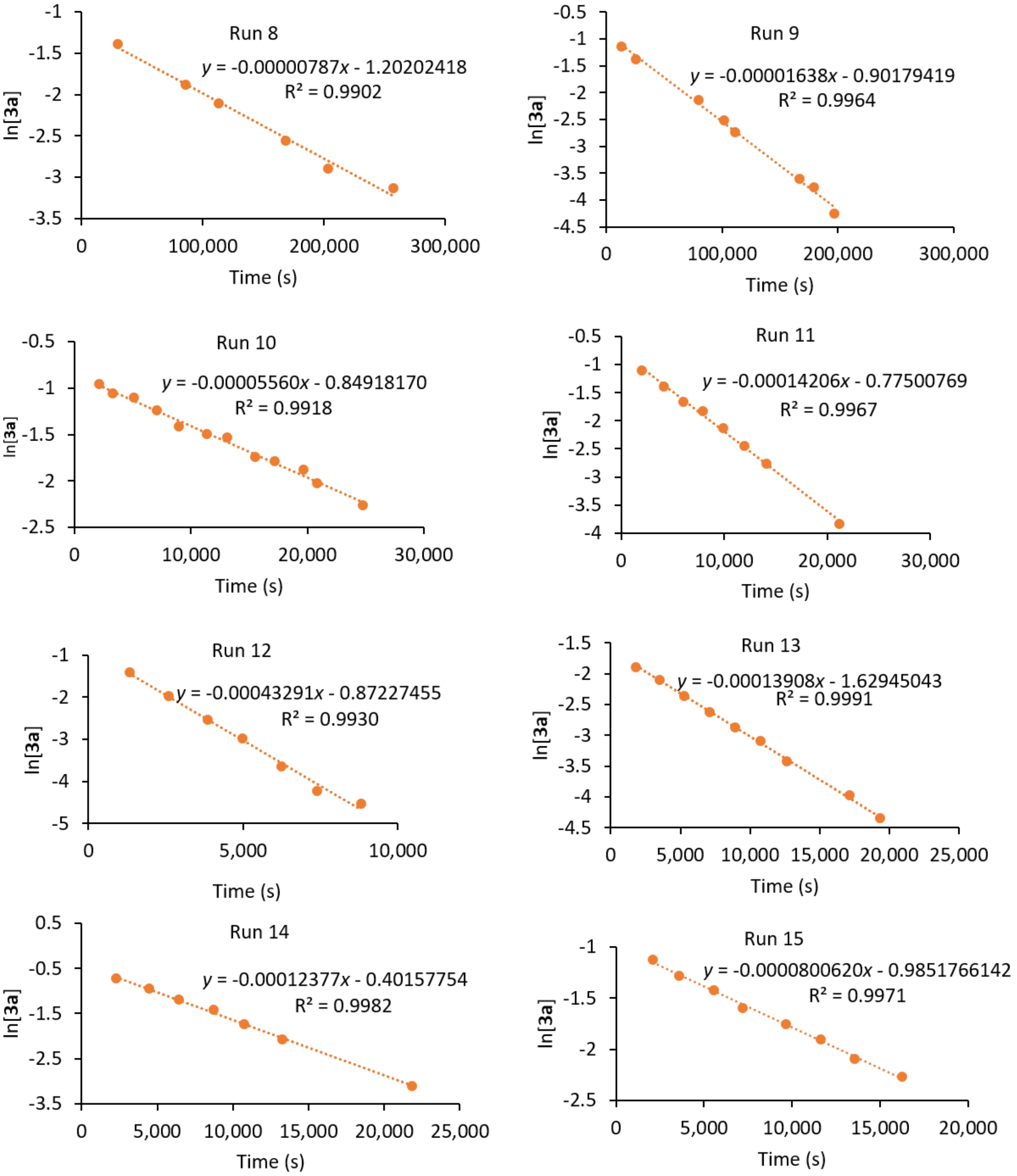

Supplementary Figure 28. Plot of ln[**3a**] with reaction time for runs 8 – 15 from Supplementary Table 2 (y = ln[**3a**], x = time (s)).

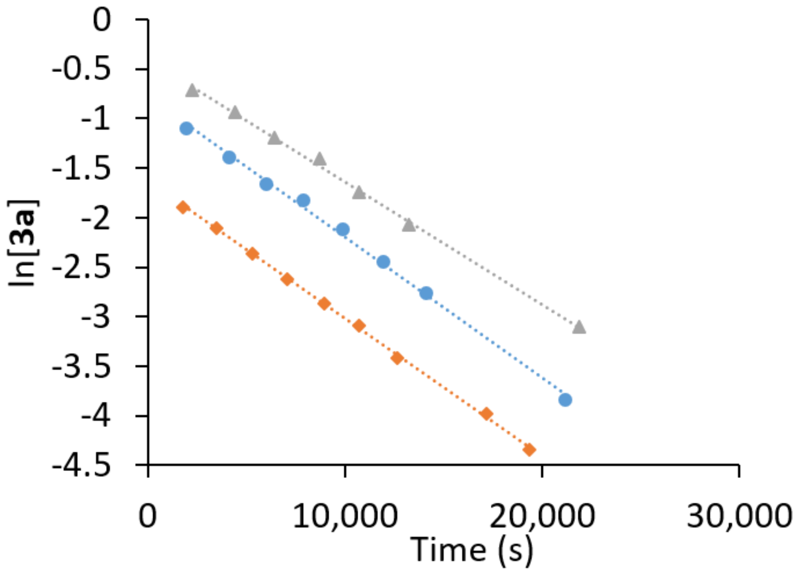

Supplementary Figure 29. Plot of ln[**3a**] with reaction time for runs 11 ([**3a**] = 0.5 M, blue circles), 13 ([**3a**] = 0.3 M, orange diamonds) and 14 ([**3a**] = 0.7 M, grey triangles) from Supplementary Table 2. The calculation of equivalent half-lives for different initial concentration of **3a** using Supplementary Equations 1 and 2 indicates unimolecular first order kinetics.

$$\ln[\mathrm{A}] = -kt + \ln[\mathrm{A}]_0 \qquad (1)$$

$$t\left(\frac{1}{2}\right) = \frac{\ln(2)}{k} \qquad (2)$$

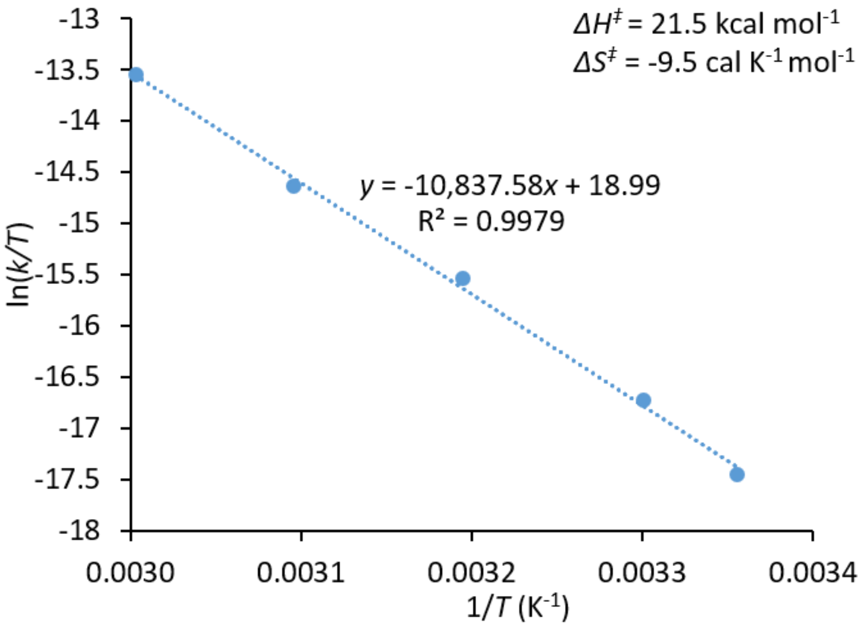

Supplementary Figure 30. Eyring plot using data from runs 8 -12 from Supplementary Table 2 used to calculate values for *ΔH*‡ and *ΔS*‡ using Supplementary Equations 3-6 (*ΔH*‡ = 21.5 kcal mol^{-1}, *ΔS*‡ = -9.5 K^{-1} mol^{-1}). (*y* = ln[**3a**], *x* = time (s)).

$$k = \frac{k_\mathrm{B}\,T}{h}\mathrm{e}^{\frac{-\Delta H^\ddagger}{RT}}\mathrm{e}^{\frac{-\Delta S^\ddagger}{R}} \quad (3)$$

$$\ln\left(\frac{k}{T}\right) = \frac{-\Delta H^\ddagger}{RT} + \ln\left(\frac{k_\mathrm{B}}{h}\right) + \frac{\Delta S^\ddagger}{R} \quad (4)$$

$$\text{Gradient} = \frac{-\Delta H^\ddagger}{R} \quad (5)$$

$$\text{Intercept} = \ln\left(\frac{k_\mathrm{B}}{h}\right) + \frac{\Delta S^\ddagger}{R} \quad (6)$$

DFT calculations

Density functional theory (DFT) calculations were carried out with the Gaussian 09 program package[12] using the hybrid exchange-correlation functional PBE0[13,14] in combination with the split-valence double-ζ basis set 6-31+G(d,p).[15–20] All *N*-heterocyclic compounds were calculated using *N*-phenyl model systems. Optimisations were carried out with tight convergence criteria and pruned (99,590) grids[21] were used for numerical integrations. Solvents [THF (ε = 7.4257) and toluene (ε = 2.3741)] were accounted for during the optimisations with the polarisable continuum model (PCM) using the integral equation formalism variant (IEFPCM).[22–25] Initial guesses for transition states were obtained by relaxed potential energy surface scans along major reaction coordinates. All stationary points were identified as minima or transition states by analytical vibrational frequency calculations. Transition states were confirmed to interconnect precursors and products by reverse and forward internal reaction coordinate (IRC) scans.[26,27] Standard Gibbs free energies are reported with thermal and zero-point energy corrections.

Schematic illustrations of the calculated reaction pathways are depicted in Supplementary Figure 31 and Supplementary Figure 32 and the calculated structures and their energy eigenvalues are summarised in Supplementary Table 3 and Supplementary Table 4. Four initial reaction steps were considered: a) P–H activation, b) B–H activation, c) substitution at boron, and d) concerted P–H/B–H activation. Whereas pathways a), b) and c) could be modelled by the calculations, attempts to locate a concerted transition state (TS) leading to (CAAC)H_2 and [PhHP-BH_2] in one step (pathway d) were unsuccessful. The P–H activation step, which first involves deprotonation of the P–H bond by the CAAC, requires a significantly low activation barrier (**TS1**: 4.9 kcal mol^{-1}; **TS1’**: 7.6 kcal mol^{-1}) when compared to B–H activation (**TS2**: 34.2 kcal mol^{-1}) and nucleophilic substitution at boron (**TS3**: 26.2 kcal mol^{-1}) (ΔG^0 values in THF are given). We therefore inferred that neither initial B–H activation nor nucleophilic substitution plays a role in product formation and focused on further pathways from the P–H proton transfer.

Two transition states (**TS1** and **TS1’**) were calculated for the deprotonation of $PhPH_2{\cdot}BH_3$ (**A**), which differ in the spatial orientation of the CAAC (**B**) with respect to the

phosphine-borane bearing a prochiral phosphorus atom. The structures of the formed ion pairs **C-1$_{pair}$** and **C-1'$_{pair}$** were derived by reverse IRC calculations and subsequent optimisation of the obtained structures. Note that the spatial orientation of $[CAAC(H)]^+$ and $[PhPH(BH_3)]^-$ in **C-1'$_{pair}$** differs from the orientation in **TS1'** and is similar to that found for **C-1$_{pair}$**, indicating that the relative orientation present in **C-1$_{pair}$** is favoured (See Supplementary Table 3). In addition, attempts to structurally optimise **C-1'$_{pair}$** with toluene as solvent only furnished only the P–H activation product **F'**.

The P–H activation product was calculated with either a S_P,*S* configuration (**F**) or a R_P,*S* configuration (**F'**), the latter being slightly higher in energy. A slightly higher activation barrier was found for the dissociation of the S_P,*S* diastereomer **F** via **TS4** (ΔG^0 = 18.7 kcal mol^{-1}, ΔH^0 = 20.2 kcal mol^{-1}) compared to the dissociation of the R_P,*S* diastereomer **F'** via **TS4'** (ΔG^0 = 16.6 kcal mol^{-1}, ΔH^0 = 19.2 kcal mol^{-1}), which is in agreement with the experimentally observed faster conversion of one diastereomer of **3a**. Attempts to obtain structures for the contact ion pairs **C-2$_{pair}$** and **C-2'$_{pair}$** by reverse IRC calculations from **TS4** or **TS4'** and subsequent optimisation of the respective structures were unsuccessful and furnished back the P-H activation products **F** or **F'**, respectively. This is characteristic of a very flat progression of the potential energy hypersurface and suggests a very low activation barrier for the formation of **F** and **F'**, which is in agreement with the experimentally found rapid formation of **3a**.

The structure of the contact ion pair **C-3$_{pair}$** was derived from a reverse IRC calculation from **TS5** and subsequent optimisation of the obtained structure. Importantly, the dissociation of **F** or **F'** followed by B–H hydride transfer to give $[PhHP\text{-}BH_2]$ (**H**) and $(CAAC)H_2$ (**G**) requires a change in the spatial orientation of the $[CAAC(H)]^+$ (**D**) cation with respect to the $[PhPH(BH_3)]^-$ (**E**) anion, as evidenced from the different orientation found in **C-3$_{pair}$**. A comparison of the energy differences of separated $[CAAC(H)]^+$ (**D**) and $[PhPH(BH_3)]^-$ (**E**) ions and the optimised ion pairs **C-1$_{pair}$**, **C-1'$_{pair}$** or **C-3$_{pair}$** reveals maximum energy differences of 2.1 kcal mol^{-1} in THF and 20.9 kcal mol^{-1} in toluene. This implies a considerably better stabilisation of the $[CAAC(H)]^+$ (**D**) and $[PhPH(BH_3)]^-$ (**E**) ions in THF and suggests a lower activation barrier for the change in the spatial orientation to access **C-3$_{pair}$** in THF than in toluene, providing a rationale

for the experimentally observed more rapid conversion of **3a** into $[PhPHBH_2]_n$ and $(CAAC^{Me})H_2$ in THF compared to toluene.

The six-membered heterocycle *all-cis*-$(PhPHBH_2)_3$ (**L**), containing all phenyl substituents in equatorial positions, was calculated to model the thermodynamic driving force for the polymerisation of $[PhPH\text{-}BH_2]$ (**H**) to $[PhHPBH_2]_n$. The calculations show that the formation of the heterocycle is favoured by 25.6 kcal mol^{-1} with respect to the $[PhPH\text{-}BH_2]$ (**H**) monomer and the overall formation of *all-cis*-$(PhPH–BH_2)_3$ and $(CAAC)H_2$ (**G**) from $PhPH_2{\cdot}BH_3$ (**A**) and CAAC (**B**) is favoured by 52.7 kcal mol^{-1}.

The heterolytic cleavage of the $P–C_{CAAC}$ bond of the P–H activation product **F** (**F’**) to separated $[CAAC(H)]^+$ (**D**) and $[PhPH(BH_3)]^-$ (**E**) ions is energetically favoured compared to the homolysis to the $[CAAC(H)]^\bullet$ and $[PhPH(BH_3)]^\bullet$ radicals. This is evident from a comparison of the computed ΔG^0 and ΔH^0 values in THF, which strongly disfavour the involvement of radicals during the formation of monomeric $[PhPH\text{-}BH_2]$ (**H**):

(1) Heterolysis of **F** (**F’**): ΔH^0 = 35.3 (34.3), ΔG^0 = 20.7 (19.5) kcal mol^{-1}

(2) Homolysis of **F** (**F’**): ΔH^0 = 55.8 (54.8), ΔG^0 = 40.1 (38.9) kcal mol^{-1}

The intermolecular hydride abstraction from **F** (**F’**) by the $[CAAC(H)]^+$ (**D**) iminium ion leading to a phosphinoborenium ion **M** (**M’**) and $(CAAC)H_2$ (**G**) has been calculated to be endergonic by ca. 18 kcal mol^{-1} (Supplementary Figure 33a).The activation barrier for this hydride abstraction step (>ca. 18 kcal mol^{-1}) is therefore significantly higher than barrier for the hydride abstraction from $[PhPH–BH_3]^-$ (**E**) via **TS5** (5.3 kcal mol^{-1}), which renders an intermolecular hydride abstraction via this route energetically unlikely.

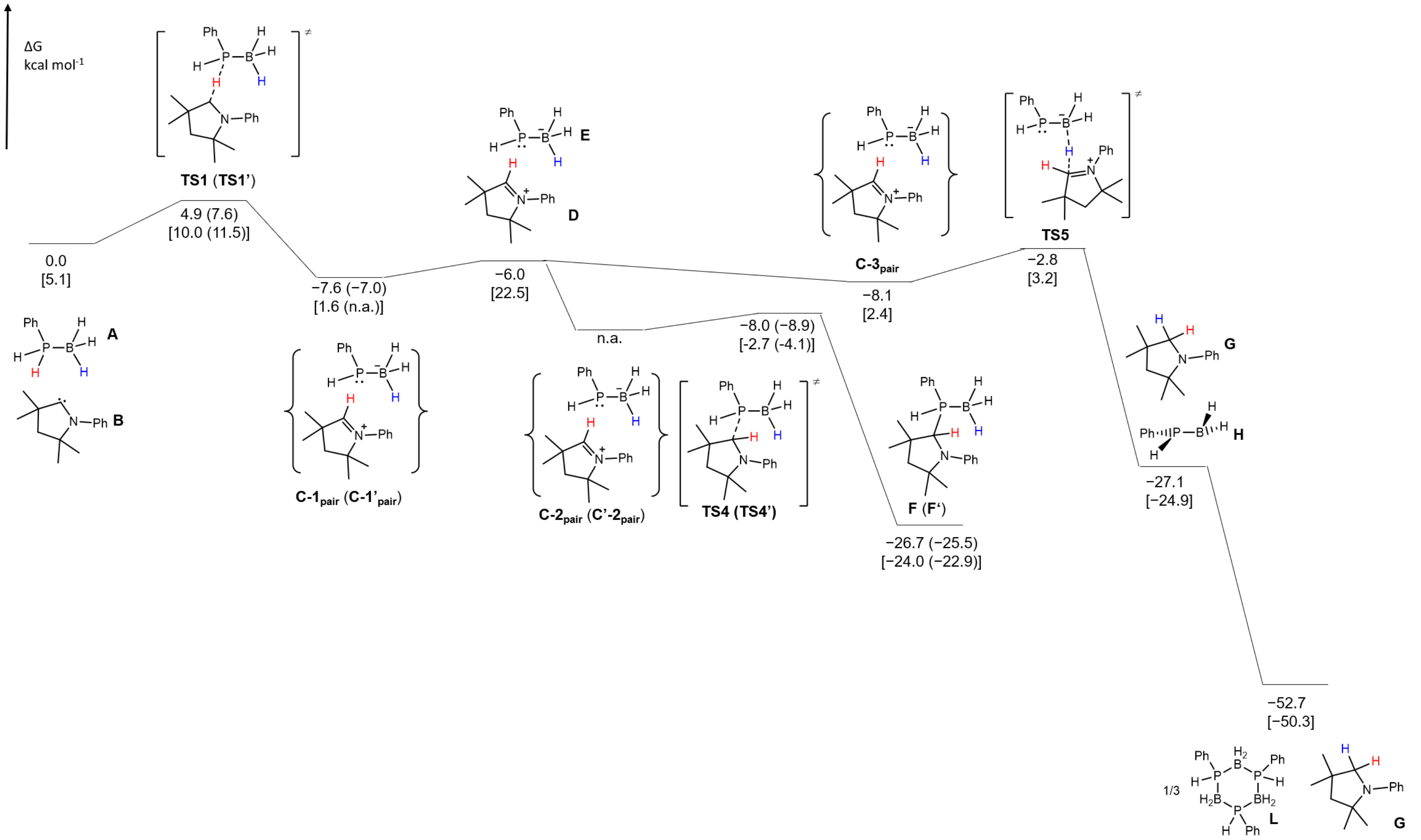

Supplementary Figure 31. Schematic Gibbs energy profile for the P–H activation of $PhPH_2{\cdot}BH_3$ (**A**) with CAAC (**B**) and subsequent reaction steps; standard Gibbs free energies are given in kcal mol^{-1} relative to those of **A** and **B** in THF; Gibbs free energies for toluene as solvent are given in square brackets.

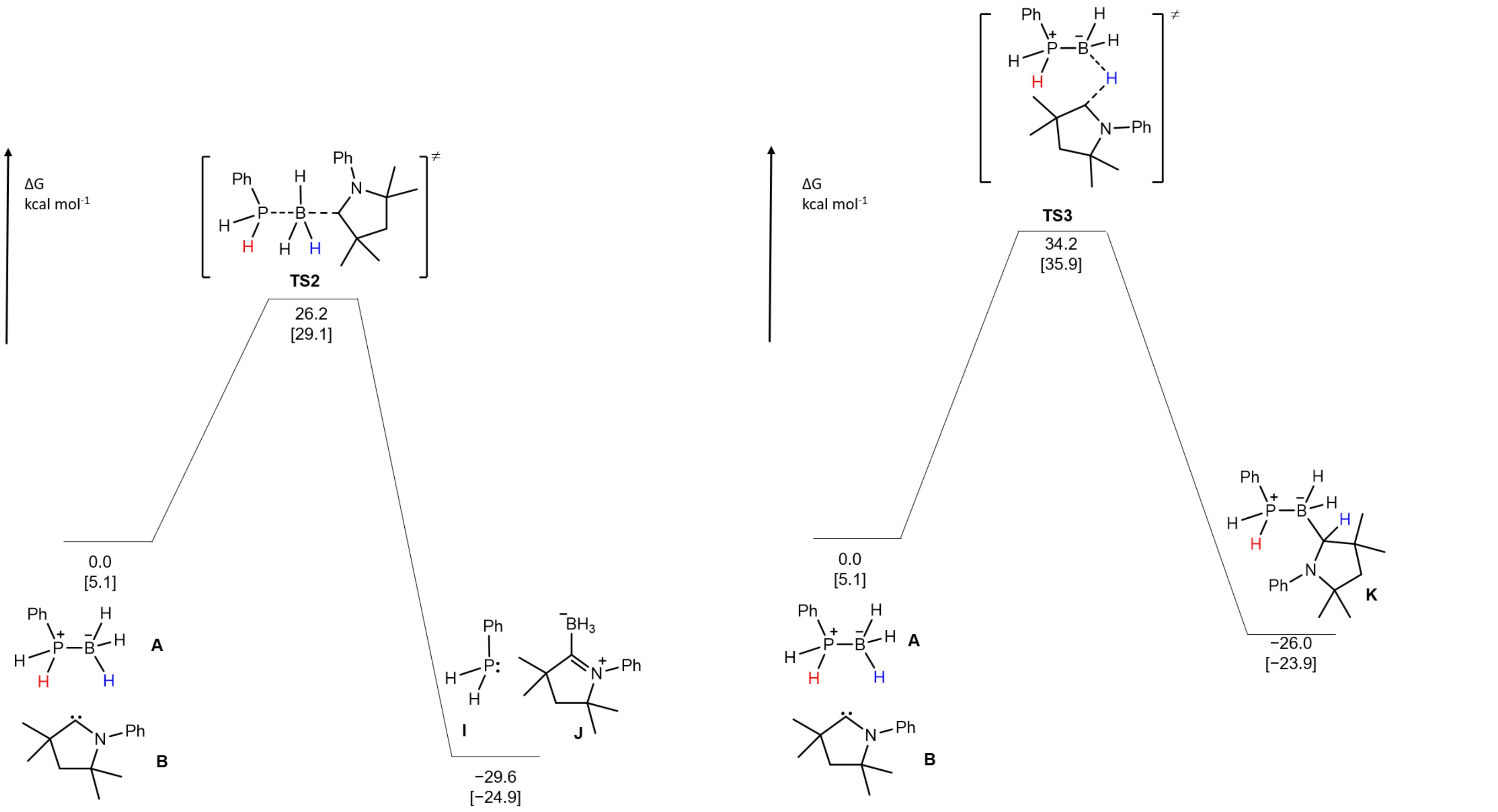

Supplementary Figure 32. Schematic Gibbs energy profiles for substitution at boron (left) and B–H activation from CAAC (**B**) and $PhPH_2 \cdot BH_3$ (**A**) (right); standard Gibbs free energies are given in kcal mol^{-1} relative to those of **A** and **B** in THF; Gibbs free energies for toluene as solvent are given in square brackets.

Supplementary Table 3: Optimised minimum structures and transition states with their imaginary frequencies in THF; standard Gibbs free energies are given below the structures in kcal mol^{-1} relative to the sum of the Gibbs free energies of **A** and **B** in THF; Gibbs free energies and imaginary frequencies for toluene as solvent are given in square brackets.

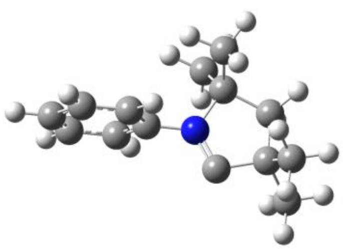	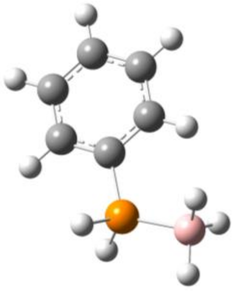	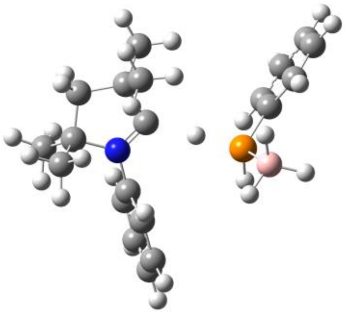
**A**	**B**	**TS1**, −426 [−677] cm^{-1}
0.0 [5.1]		4.9 [10.0]
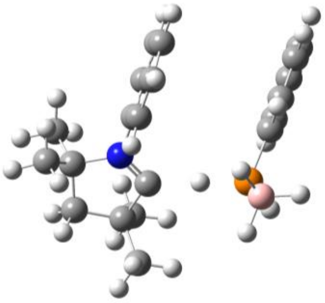	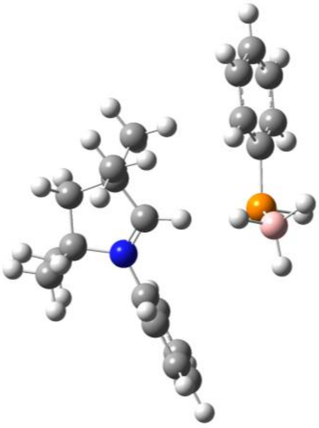	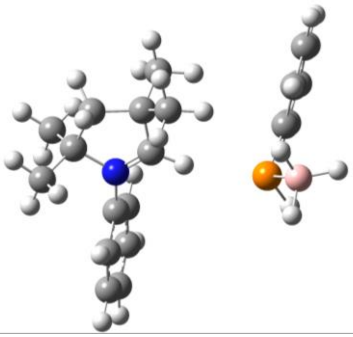
**TS1’**, −446 [−695] cm^{-1}	**C-1$_{pair}$**	**C-1’$_{pair}$**
7.6 [11.5]	−7.6 [1.6]	−7.0 [n.a.][a]
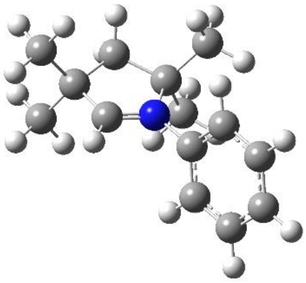	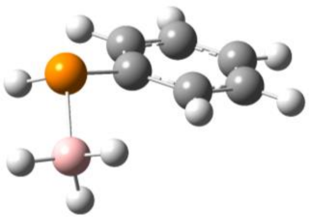	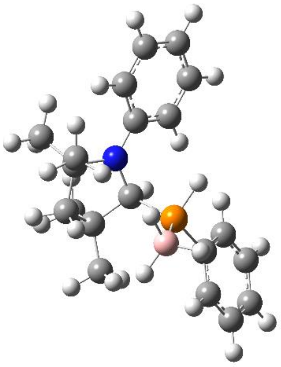
**D**	**E**	**F**
−6.0 [22.5]		−26.7 [−24.0]

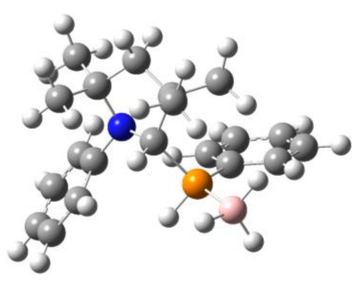	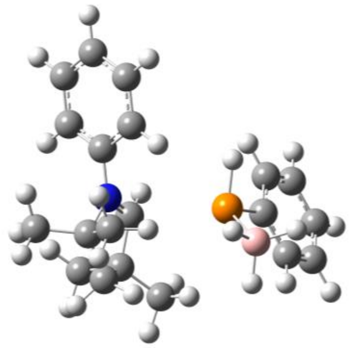	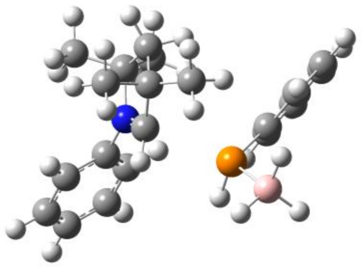
**F’**	**TS4**, −91.3 [−96.5] cm^{-1}	**TS4’**, −122.5 [−120.2] cm^{-1}
−25.5 [−22.9]	−8.0 [−2.7]	−8.9 [−4.1]
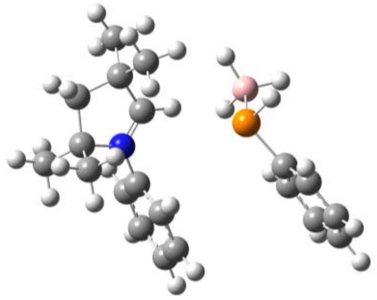	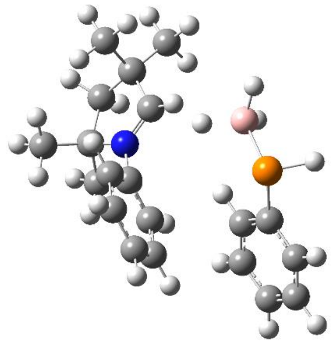	
**C-3**$_{\mathbf{pair}}$	**TS5**, −181 [−70] cm^{-1}	
−8.1 [2.4]	−2.8 [3.2]	
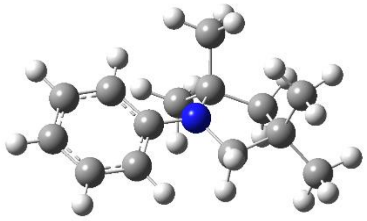	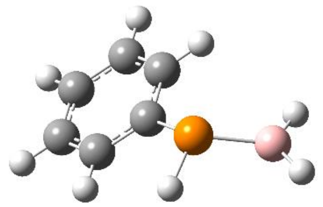	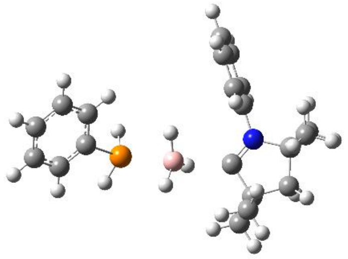
**G**	**H**	**TS2**, −404 [−390] cm^{-1}
−27.1 [−24.9]		26.2 [29.1]

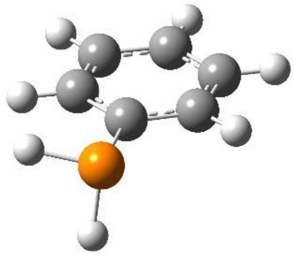	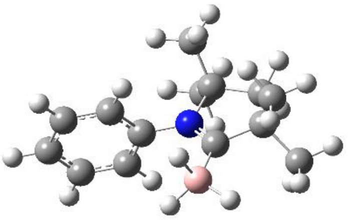	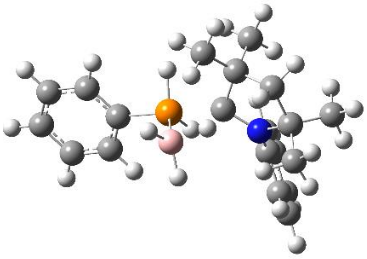
**I**	**J**	**TS3**, −219 [−194] cm^{-1}
−29.6 [−24.9]		34.2 [35.9]
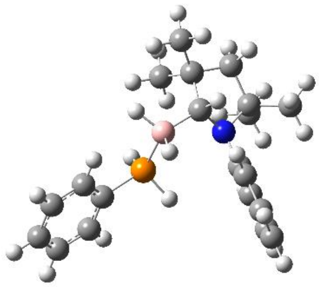	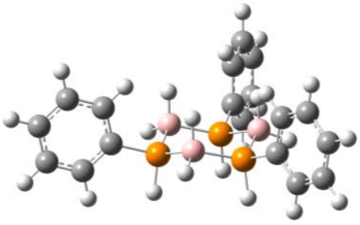	
**K**	**L**	
−26.0 [−23.9]	−52.7 [−50.3][b]	

[a] Structural optimisation of **C-1’$_{pair}$** in toluene was unsuccessful.

[b] The Gibbs free energies for the formation of **L** were calculated using the formula $\Delta G^0 = [1/3\cdot\Delta G^0(\mathbf{L}) + \Delta G^0(\mathbf{G})] - [\Delta G^0(\mathbf{A})_{THF} - \Delta G^0(\mathbf{B})_{THF}]$.

Supplementary Table 4: Calculated isodesmic reaction between $[CAAC(H)]^+$ and $(NHC)H_2$; Gibbs free energies are given below the structures in kcal mol^{-1} in the gas phase.

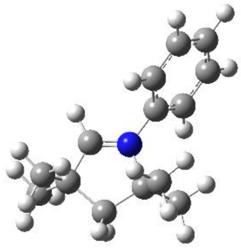	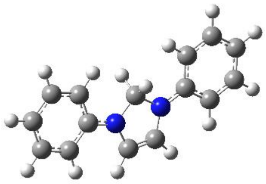	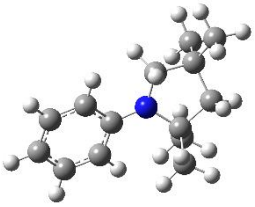	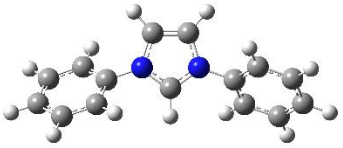
$[CAAC(H)]^+$	NHC-H_2	CAAC-H_2	$[NHC(H)]^+$
0.0		−66.8	

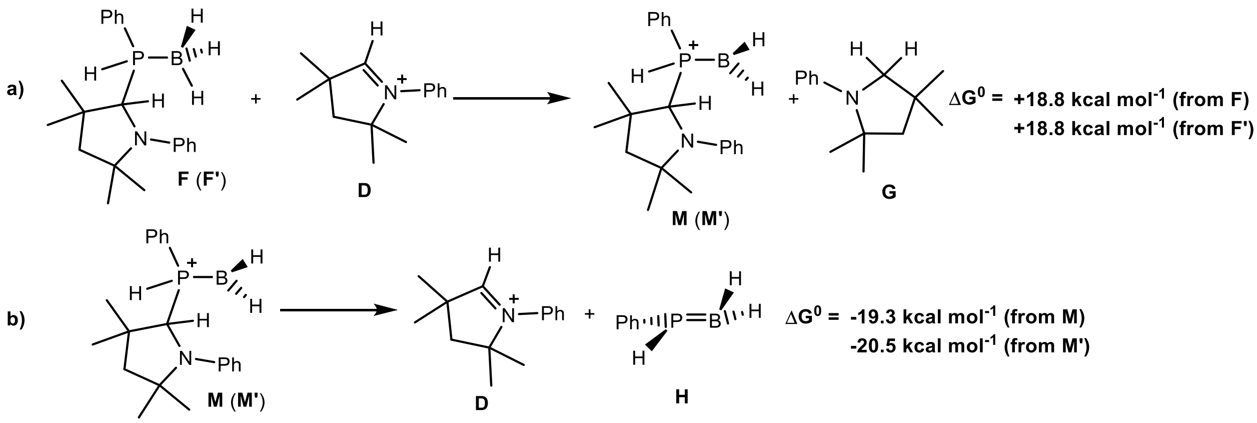

Supplementary Figure 33. Schemes depicting the intermolecular hydride abstraction pathway: a) intermolecular hydride abstraction from **F** (**F'**) by the [CAAC(H)]$^{+}$ (**D**) iminium ion leading to a phosphinoborenium ion **M** (**M'**) and CAAC(H_2) (**G**) and b) subsequent conversion of **M** (**M'**) to **D** and phosphinoborane [PhHP-BH_2] (**H**); standard Gibbs free energies are given in THF.

Supplementary Table 5: Optimised minimum structures of **M** and **M'**.

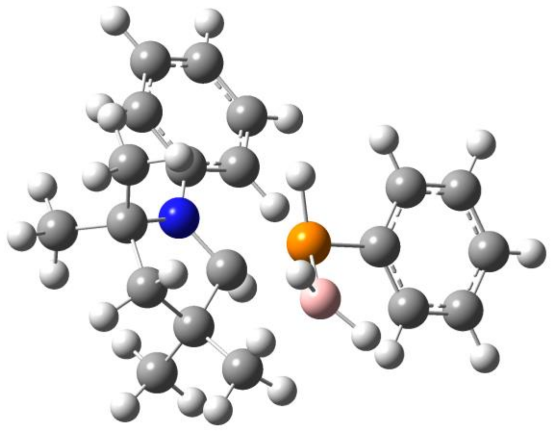	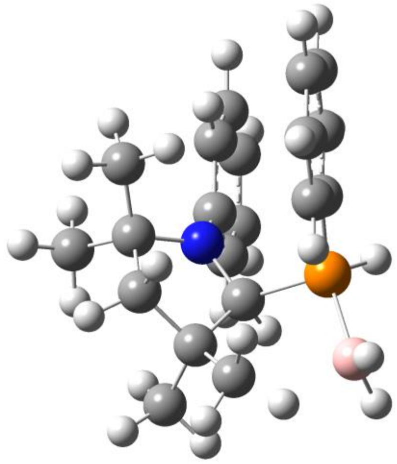
**M**	**M'**

Polymerisation attempts of P-disubstituted phosphine-boranes using CAAC^{Me} and CAAC^{Cy}

Synthesis of **3b**

Method A:

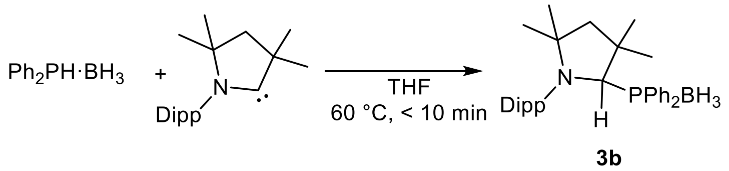

$Ph_2PH{\cdot}BH_3$ (500 mg, 2.50 mmol) and CAAC^{Me} (714 mg, 2.50 mmol) were dissolved in THF (2 mL) in a J. Young Schlenk tube and immediately at 22 °C a white precipitate was observed. The vessel was sealed, and the reaction mixture was heated to 60 °C, after ten minutes the solution became homogeneous. Similar to the case of compound **3a**, isolation of compound **3b** has not been achieved due to significant polymerisation occurring immediately at 22 °C.

It is proposed that the initial precipitate is due to initial deprotonation of the phosphine-borane to form ionic [CAAC^{Me}H][Ph_2PBH_3], as is observed in the reaction between $PhRPH{\cdot}BH_3$ (R= H or Ph) and IDipp, prior to the formation of the neutral molecular species by cation-anion recombination.

Method B:

3b can also be synthesised through a stepwise procedure

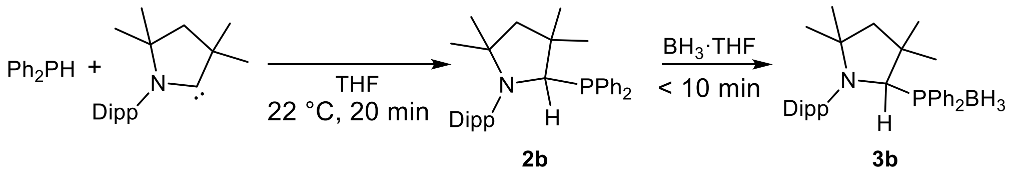

Ph_2PH (250 mg, 1.34 mmol) was added to a solution of CAAC^{Me} (383 mg, 1.34 mmol) in THF (1 mL) in a J. Young Schlenk tube, the vessel was sealed and stirred for 20 minutes at 22 °C. $BH_3{\cdot}THF$ (1.34 mL of a 1M in THF solution, 1.34 mmol) was added and the reaction mixture was stirred for 10 minutes before ^{11}B and ^{31}P NMR spectra were recorded.

Method C:

3b was also independently synthesised through a salt metathesis reaction

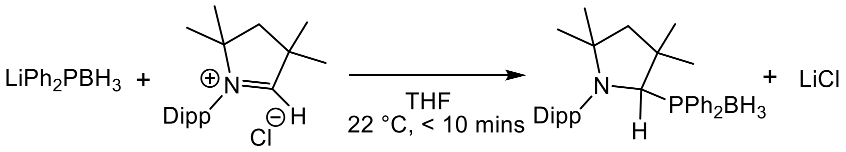

3b

$LiPh_2PBH_3$ (15 mg, 0.07 mmol) and [CAAC^{Me}H]Cl (23 mg, 0.07 mmol) were dissolved in THF (0.5 mL) in a quartz J. Young NMR tube. Immediate conversion to the products was observed and the identity confirmed using ^{11}B and ^{31}P NMR spectroscopy. No purification of the product formed from this method was carried out.

^{11}B NMR (96 MHz, 22 °C, THF): δ = -39.1 (br).

^{31}P NMR (122 MHz, 22°C, THF): δ = 10.9 (br).

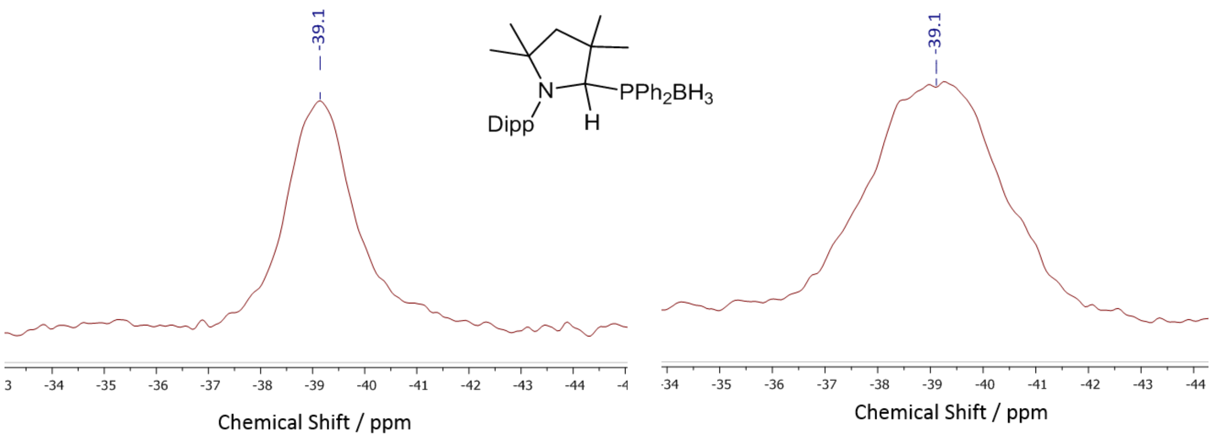

Supplementary Figure 34. ^{11}B{^{1}H} (left) and ^{11}B (right) NMR spectra (96 MHz, 22 °C, THF) of **3b**.

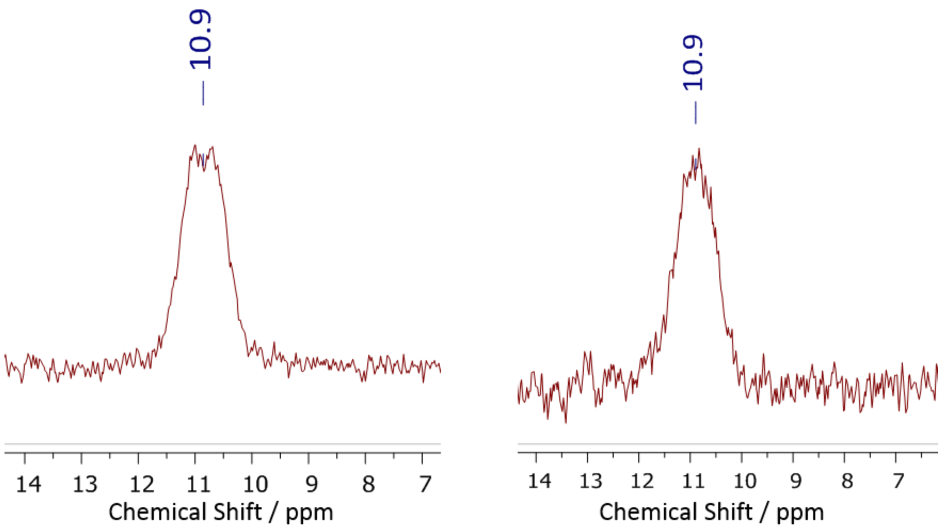

Supplementary Figure 35. $^{31}P\{^{1}H\}$ (left) and ^{31}P (right) NMR spectra (122 MHz, 22 °C, THF) of **3b**

Dehydropolymerisation of $Ph_2PH{\cdot}BH_3$

Method A:

CAAC*Me* in THF

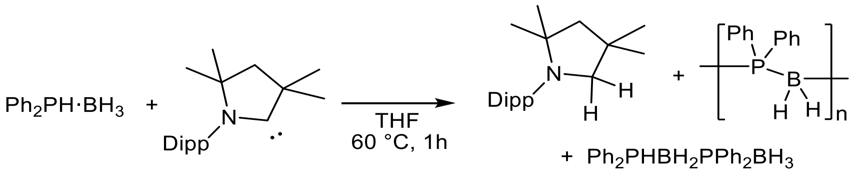

$Ph_2PH{\cdot}BH_3$ (500 mg, 2.50 mmol), CAAC^{Me} (714 mg, 2.50 mmol) and THF (1 mL) were added to a J. Young Schlenk and heated as a closed system at 60 °C for one hour. ^{31}P NMR of the crude reaction mixture showed the formation of $Ph_2PHBH_2PPh_2BH_3$ (~ 25 %) and $[Ph_2PBH_2]_n$ (Supplementary Figure 40). The simultaneous formation of cyclic oligomers of the form $[Ph_2PBH_2]_x$ cannot be unambiguously ruled out as the phosphorus and boron chemical shifts coincide with those of the internal atoms of the linear dimer. The reaction mixture was added dropwise into 20 mL of rapidly stirred cold hexanes at -40 °C yielding a precipitate and the supernatant was decanted. The precipitation was repeated twice more prior to drying in vacuo to leave a white powder which by ^{11}B and ^{31}P NMR analysis was determined to be a mixture of $Ph_2PHBH_2Ph_2PBH_3$ (~ 35 %) and $[Ph_2PBH_2]_n$ (Supplementary Figure 40). Attempts to

separate the product further by precipitation into acetonitrile, or through washing with diethyl ether were unsuccessful. GPC analysis showed only a very small amount of high molar mass material which it was not possible to analyse. Yield (precipitated material) = 260 mg (52 %).

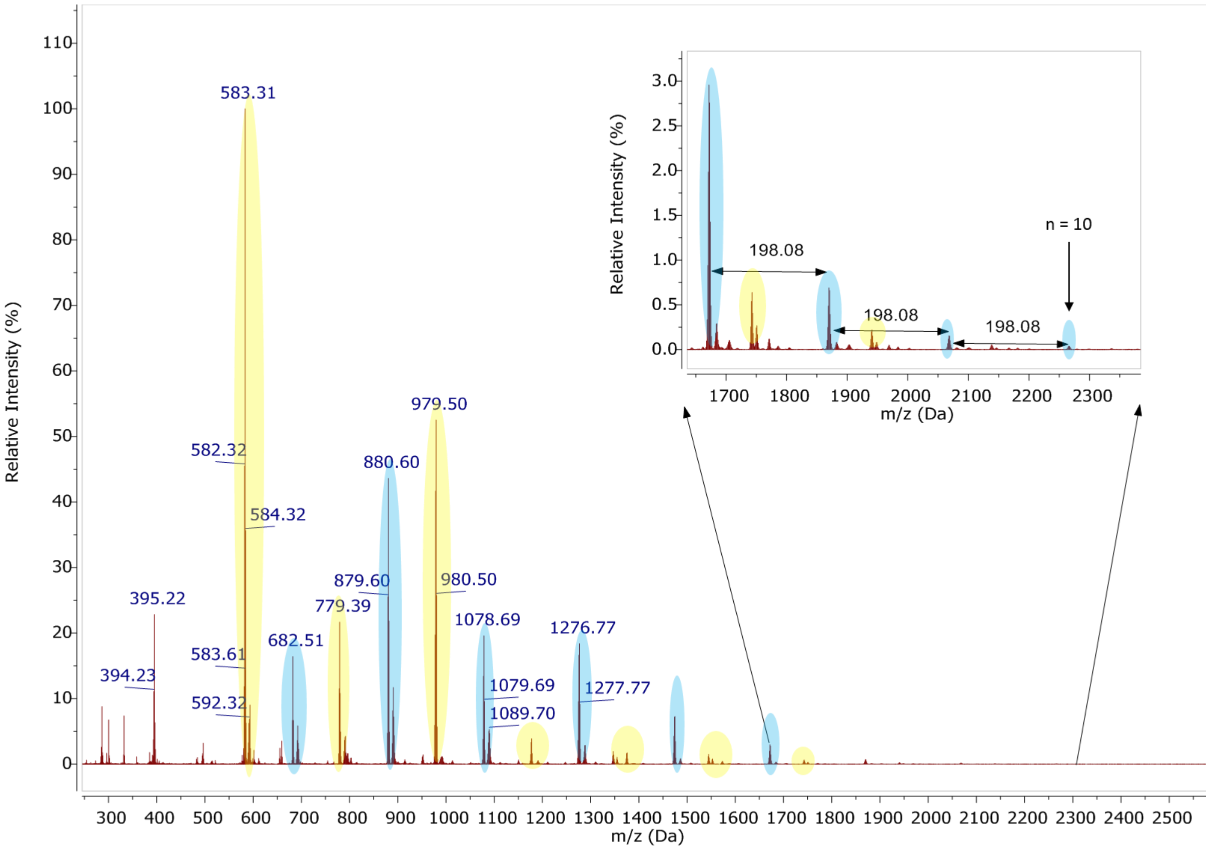

Supplementary Figure 36. ESI(+)-MS spectrum in positive mode of unseparated $[Ph_2PBH_2]_n$ and $Ph_2PHBH_2Ph_2PBH_3$ (m/z = 398.17) formed from $Ph_2PH{\cdot}BH_3$ and CAAC^{Me} in THF. The species highlighted in yellow is a linear system with a Ph_2PH end group $(H\text{-}[Ph_2PBH_2]_n\text{-}Ph_2PH)^+$ and the species highlighted in blue is a linear system with a CAAC^{Me} end group $(H\text{-}[Ph_2PBH_2]_n\text{-}CAAC^{Me})^+$.

Method B:

CAAC^{Me} in toluene

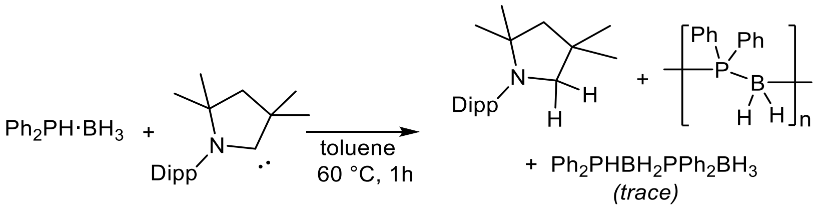

$Ph_2PH{\cdot}BH_3$ (200 mg, 1.00 mmol), CAAC^{Me} (285 mg, 1.00 mmol) and toluene (400 µL) were added to a J. Young NMR tube and heated in a closed system at 60 °C for one hour. ^{31}P NMR of the crude reaction mixture showed the formation of $Ph_2PHBH_2PPh_2BH_3$ (~ 8 %) and $[Ph_2PBH_2]_n$. The reaction mixture was added dropwise into 20 mL of rapidly stirred cold hexanes at -40 °C yielding a precipitate and the supernatant was decanted. The precipitation was repeated twice more prior to drying in vacuo to leave a white powder. GPC analysis showed a bimodal distribution with a small amount (ca. 10 %) of high molar mass material (Supplementary Figure 38). Yield (precipitated material) = 26 mg (25 %).

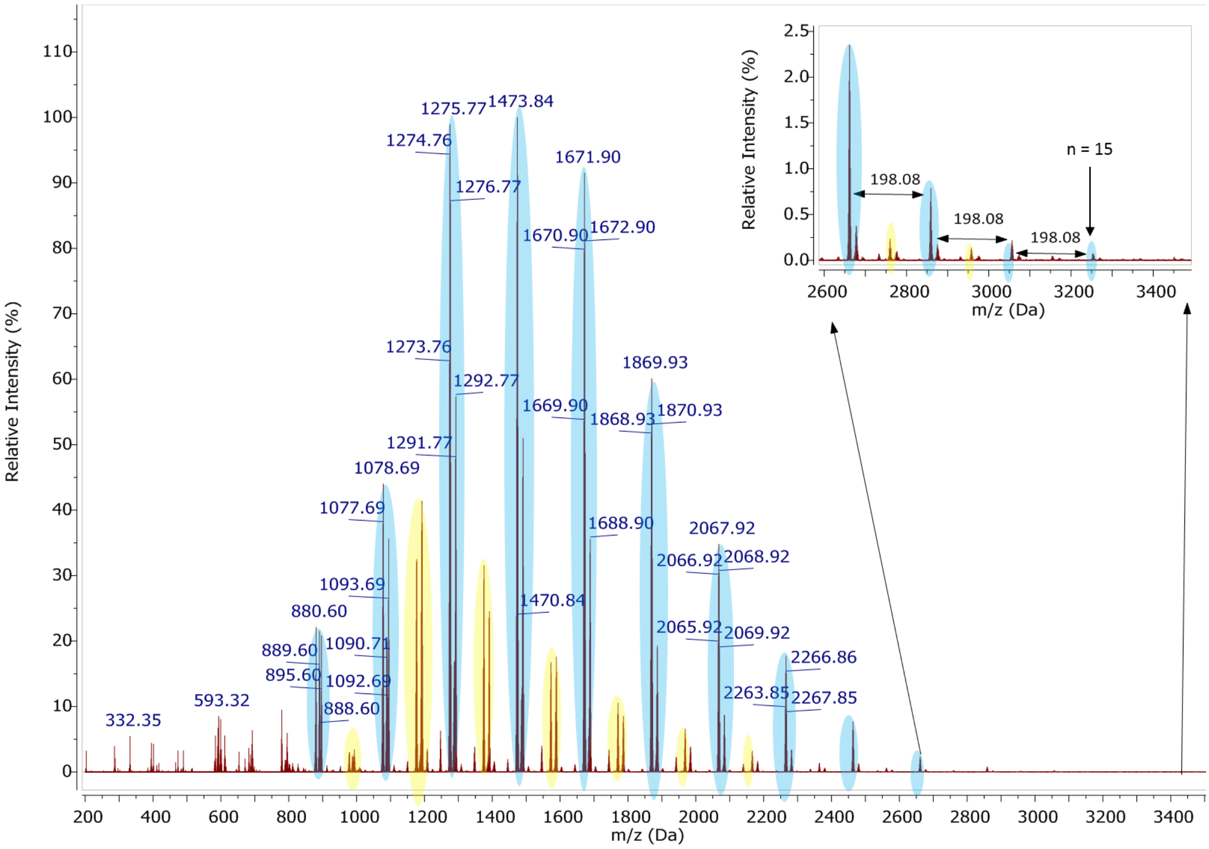

Supplementary Figure 37. ESI(+)-MS spectrum in positive mode of $[Ph_2PBH_2]_n$ formed from $Ph_2PH{\cdot}BH_3$ and CAAC^{Me} and in toluene. The species highlighted in yellow is a linear system with a Ph_2PH end group $(H\text{-}[Ph_2PBH_2]_n\text{-}Ph_2PH)^+$ and the species highlighted in blue is a linear system with a CAAC^{Me} end group $(H\text{-}[Ph_2PBH_2]_n\text{-}CAAC^{Me})^+$.

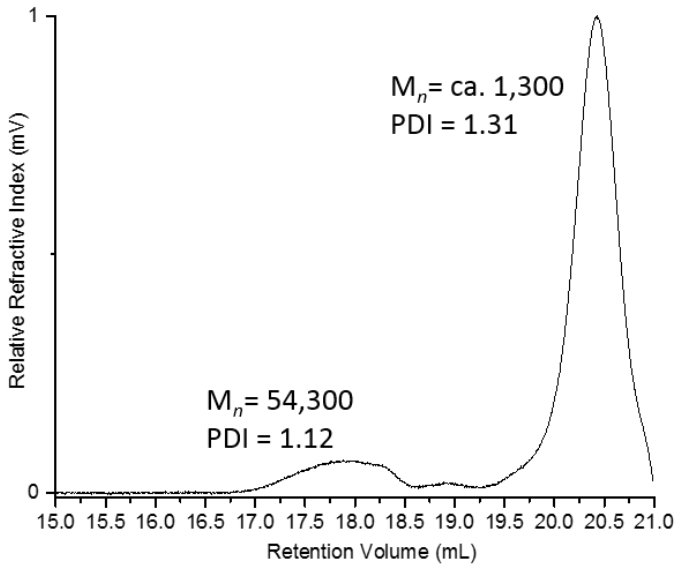

Supplementary Figure 38. GPC chromatogram of $[Ph_2PBH_2]_n$ formed from $Ph_2PH·BH_3$ and CAAC^{Me} in toluene (2 mg mL^{-1} in THF with 0.1 w/w % *n*Bu_4NBr in the THF eluent). The highest molar mass peak accounts for ca. 10 % of the precipitated material. The bimodal distribution can be explained by the competition between chain termination and propagation. Most of the material undergoes early termination, potentially by addition of a free CAAC^{Me} unit as evidenced in the ESI-MS, whereas a small percentage of the material undergoes significant further polymerisation to give high molar mass material.

Method C:

CAAC^{Cy} in toluene

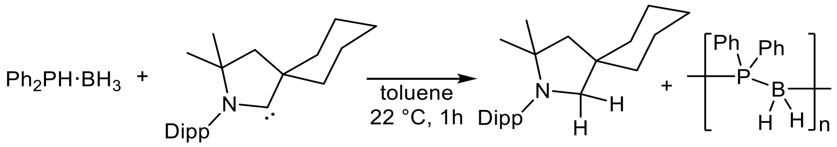

$Ph_2PH·BH_3$ (200 mg, 1.00 mmol), CAAC^{Cy} (325mg, 1.00 mmol) and toluene (400 µL) were added to a J. Young NMR tube. After 1 hour at 22 °C ^{31}P NMR of the crude reaction mixture showed the complete consumption of $Ph_2PH·BH_3$ and formation of $[Ph_2PBH_2]_n$. The reaction mixture was added dropwise into 20 mL of rapidly stirred cold hexanes at -40 °C yielding a precipitate

and the supernatant was decanted. The precipitation was repeated twice more prior to drying in vacuo to leave an off-white powder. GPC analysis showed a bimodal distribution with a small amount (ca. 12 %) of high molar mass material. Yield (precipitated material) = 95 mg (48 %).

An attempt to further separate the high molar mass component observed in the GPC chromatogram from lower mass oligomers was made. The material was dissolved in THF (5 mL) and a small quantities of hexanes (5 mL portions) until some material precipitated. The supernatant was removed to leave fraction A. The process was repeated, adding further 5 mL portions of hexanes to the supernatant each time, to give three further fractions of product. The results of this separation attempt are reported below and show that isolation of the pure high molar mass fraction was unsuccessful, although some fractionation was possible.

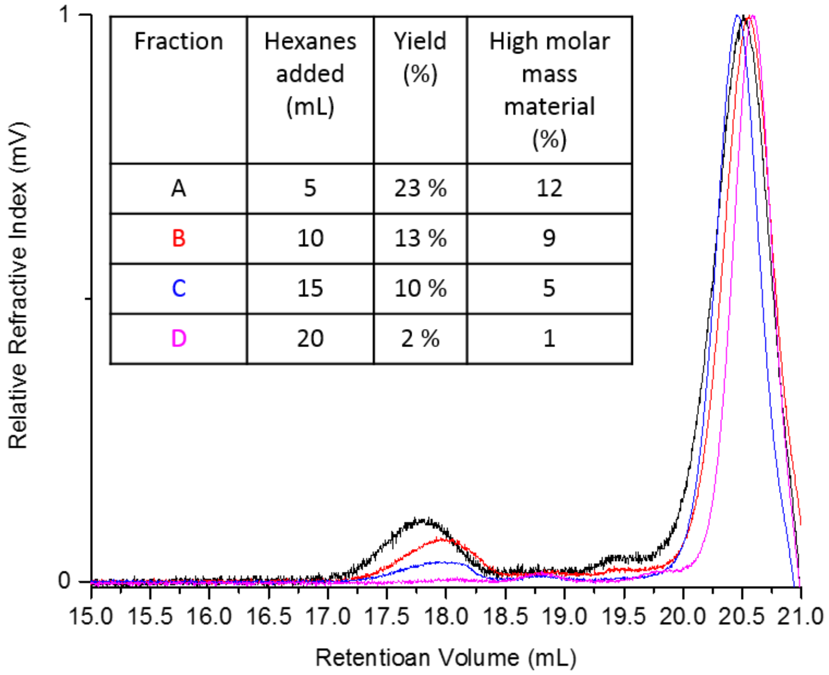

Fraction	Hexanes added (mL)	Yield (%)	High molar mass material (%)
A	5	23 %	12
B	10	13 %	9
C	15	10 %	5
D	20	2 %	1

Supplementary Figure 39. GPC chromatograms of the separation attempts of the $[Ph_2PBH_2]_n$ formed from $Ph_2PH{\cdot}BH_3$ and CAAC^{Cy} in toluene (2 mg mL^{-1} in THF with 0.1 w/w % *n*Bu_4NBr in the THF eluent). The GPC chromatograms have been normalised to the low molar mass material. Although the stepwise precipitation of material does show an increase in the percentage of high molar mass material for the earlier fraction there is still not a significant amount. For Fraction A M_n = 59,600 and PDI = 1.08, however, since the standard used for the calibration of the GPC instrument is polystyrene $[PhCHCH_2]_n$, the intrinsic values shown here may not be accurate especially as the presence of a second phenyl group will most likely reduce the amount of coiling of the polymer chains in solution.

NMR data is given for Fraction A

^{1}H NMR (400 MHz, 25 °C, $CDCl_3$): δ = 7.50- 6.50 (m, br, Ar), 2.85 – 0.25 (m, br, BH_2)

^{11}B NMR (96 MHz, 22 °C, $CDCl_3$): δ = -30.4 (br)

^{31}P NMR (122 MHz, 22 °C, $CDCl_3$): δ = -15.2, -16.6 (br)

It is postulated that three polymer architectures are formed in the head-to-tail polymerisation step: small cyclic oligomers, short chain linear oligomers and long chain linear polymers.

In an attempt to explain the differences in the products formed under the different conditions (THF vs toluene, CAAC^{Me} vs CAAC^{Cy}) we compared the ^{31}P NMR spectra of the crude material and after precipitation into hexanes (Supplementary Figure 40). For CAAC^{Me} in THF $Ph_2PHBH_2PPh_2BH_3$ (δ = -3.3 ppm, -18.2 ppm [$CDCl_3$]) is detected both before and after precipitation along with a broad peak (δ = -15.6 ppm [$CDCl_3$]). When CAAC^{Me} is used in toluene a much smaller quantity of $Ph_2PHBH_2PPh_2BH_3$ is observed, however in the crude material there are two obvious broad peaks (δ = -15.7 ppm and -18.3 ppm [toluene]). The peak at -18.3 ppm is removed by precipitation. It is this coupled with the similarity in the shift to $[Ph_2PBH_2]_{3\text{ or }4}$[8] which leads us to identify the product as small cyclic species. When using the more reactive CAAC^{Cy} only a very small amount of small cyclics are formed (δ = -18.3 [toluene]), postulated to be due to the greater reactivity of CAAC^{Cy} leading to a higher concentration of monomer in solution thereby favouring oligomerisation over small cyclic oligomers. This is also evidenced by the higher yield after precipitation when using CAAC^{Cy} as opposed to CAAC^{Me}. The bimodal appearance of the broad peak observed after precipitation can be explained by the presence of both oligomeric and polymeric products, as evidenced by the GPC chromatograms.

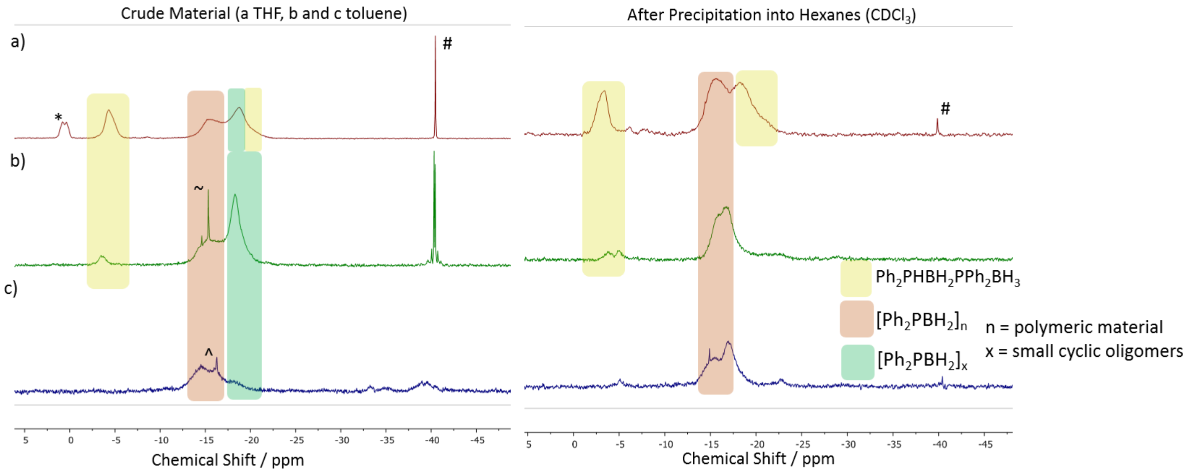

Supplementary Figure 40. ^{31}P NMR of products of the dehydropolymerisation of $Ph_2PH{\cdot}BH_3$ using a) CAAC^{Me} in THF, b) CAAC^{Me} in toluene and c) CAAC^{Cy} in toluene before and after precipitation into hexanes (* denotes excess $Ph_2PH{\cdot}BH_3$, ~ denotes CAAC^{Me}(H)PPH_2, ^ denotes CAAC^{Cy}(H)PPH_2, # denotes Ph_2Ph).

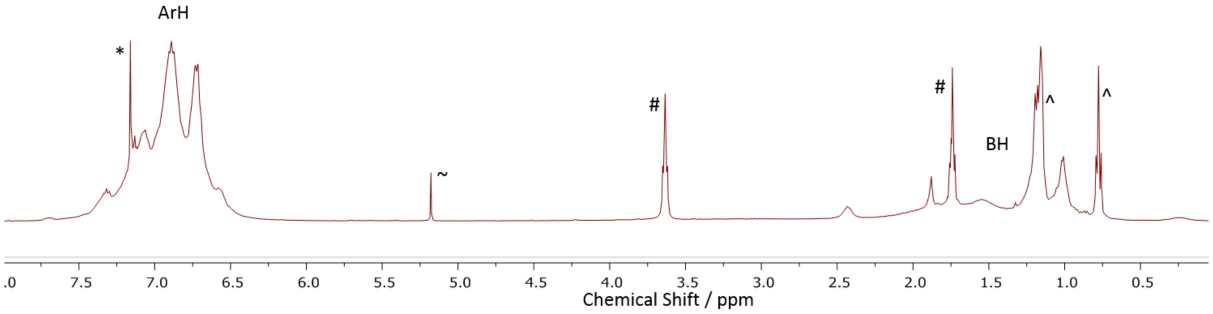

Supplementary Figure 41. ^{1}H NMR spectrum (400 MHz, 25 °C, $CDCl_3$) of $[Ph_2PBH_2]_n$ formed from $Ph_2PH{\cdot}BH_3$ and CAAC^{Cy} in toluene (Fraction A) (* denotes partially protiated $CDCl_3$, ~ denotes trace DCM, # denotes trace THF, ^ denotes trace hexanes).

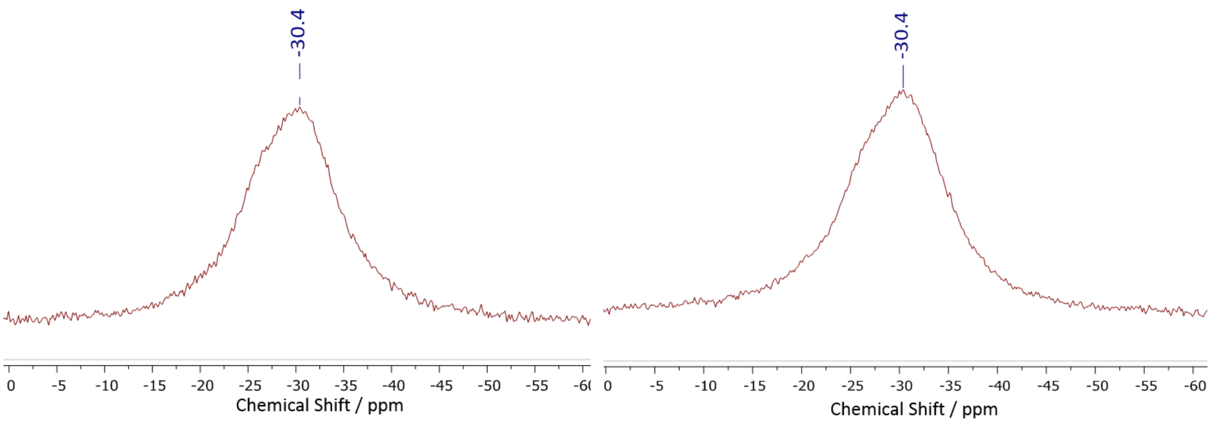

Supplementary Figure 42. $^{11}B\{^1H\}$ (left) and ^{11}B (right) NMR spectra (96 MHz, 22 °C, $CDCl_3$) of $[Ph_2PBH_2]_n$ formed from $Ph_2PH{\cdot}BH_3$ and CAAC^{Cy} in toluene. (Fraction A).

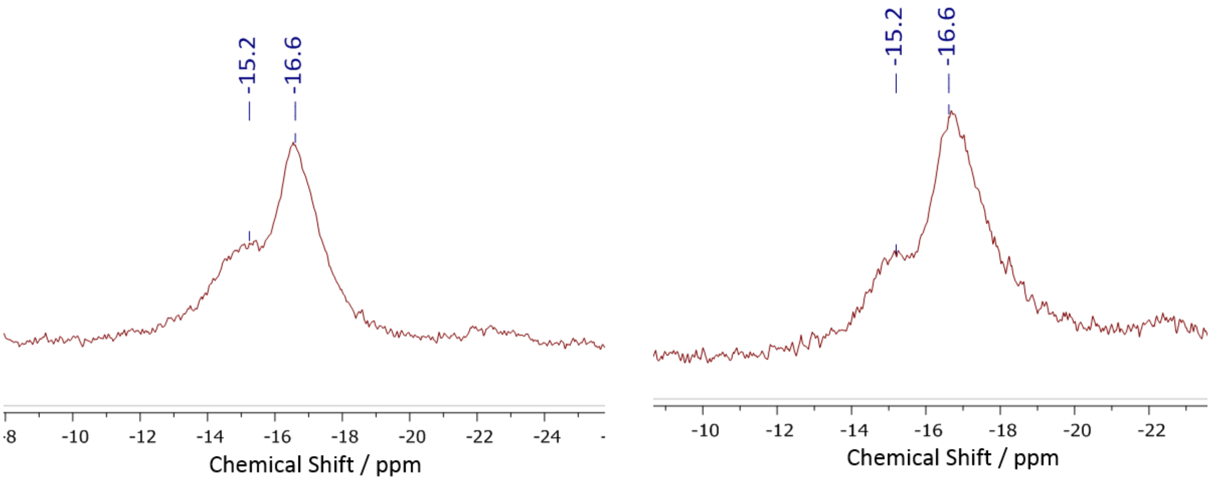

Supplementary Figure 43. $^{31}P\{^1H\}$ (left) and ^{31}P (right) NMR spectra (122 MHz, 22 °C, $CDCl_3$) of $[Ph_2PBH_2]_n$ formed from $Ph_2PH{\cdot}BH_3$ and CAAC^{Cy} in toluene. (Fraction A). The observation of two peaks can be explained by the presence of both oligomeric and polymeric material, as is observed in the GPC chromatogram.

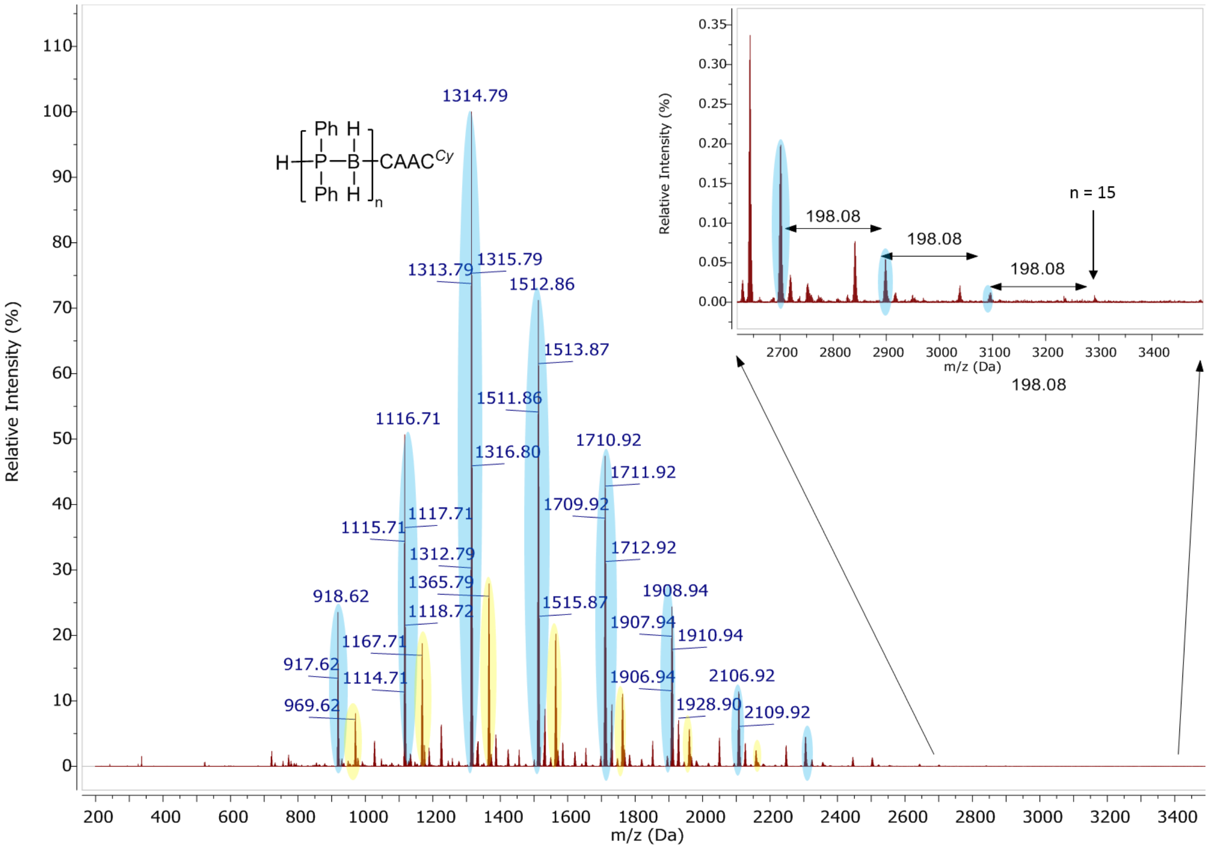

Supplementary Figure 44. ESI(+)-MS spectrum in positive mode of $[Ph_2PBH_2]_n$ formed from $Ph_2PH{\cdot}BH_3$ and CAAC^{Cy} in toluene (Fraction A). The species highlighted in blue is a linear system with a CAAC^{Cy} end group (H-$[Ph_2PBH_2]_n$-CAAC^{Cy})$^+$ and the species highlighted in yellow is a linear system with an unidentified end group (m/z of end group = 178.31).

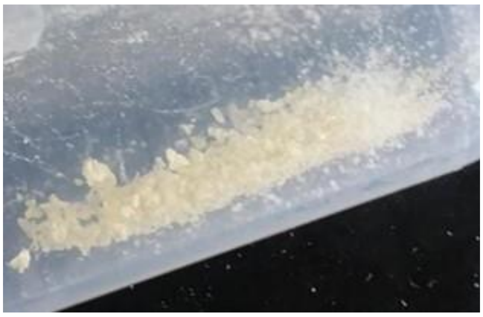

Supplementary Figure 45. Photograph of $[Ph_2PBH_2]_n$ (Fraction A). Photograph taken by N. L. Oldroyd.

<u>Synthesis of **3c**</u>

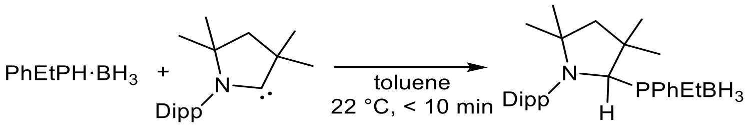

3c

rac-PhEtHP·BH_3 (50 mg, 0.33 mmol) and CAAC^{Me} (94 mg, 0.33 mmol) were dissolved in toluene (2 mL) in a J. Young Schlenk tube. Immediate conversion to the product (as two diastereomers **3c'** and **3c''**) was observed through ^{31}P NMR spectroscopy. The initial ratio of diastereomers observed after 10 minutes in solution was 1 : 1.14. Crystals of **3c** suitable for X-ray crystallography of one diastereomer were obtained from layering a THF solution with hexane at -40 °C. Yield = 54 mg (38 %).

^{1}H NMR (500 MHz, 22 °C, $CDCl_3$): δ = 8.07-7.98 (m, 2H, Ar^{p}), 7.50-7.38 (m, 3H, Ar^{o,m}),), 7.20-7.05 (m, 3H, Ar^{Dipp}), 4.51 (d, $^{2}J_{PH}$ = 6.9 Hz, 1H, NC*H*), 4.10 (sept, $^{3}J_{HH}$ = 6.8 Hz, 1H, C*H*(CH_3)$_2$), 3.07 (sept, $^{3}J_{HH}$ = 6.8 Hz, 1H, C*H*(CH_3)$_2$), 2.02, 1.99 (d, $^{2}J_{HH}$ = 213 Hz), 1.48 (d, $^{3}J_{HH}$ = 6.8 Hz, 6H, CH(CH_3)$_2$), 1.47 (s, 3H, C(CH_3)$_2$), 1.43 (s, 3H, NC(CH_3)$_2$), 1.36 (dm, 2H, PCH_2), 1.33 (d br, 3H, CH(CH_3)$_2$), 1.28 (d, $^{3}J_{HH}$ = 6.8 Hz, 6H, CH(CH_3)$_2$), 1.24 (d, $^{3}J_{HH}$ = 6.8 Hz, 6H, CH(CH_3)$_2$), 1.15 (s br, 3H, C(CH_3)$_2$), 0.91 (s, 3H, NC(CH_3)$_2$), 0.54 (m, 3H, , PCH_2CH_3). Unable to assign BH_3 as broad and overlapping other signals.

^{13}C (101 MHz, 25 °C, $CDCl_3$): δ = 149.0 (Ar^{Dipp-o}), 146.7 (Ar^{Dipp-o}), 145.4 (Ar^{Dipp-i}), 133.7 (Ar^{m}), 130.8, (Ar^{p}), 127.9 (Ar^{i}), 126.5 (Ar^{Dipp-p}), 126.0 (Ar^{Dipp-m}), 124.4 (Ar^{Dipp-m}), 77.0 (N*C*H), 64.4 (NC(CH_3)$_2$*C*H_2), 43.4 (NCHP*C*(CH_3)$_2$), 32.2 (C(*C*H_3)$_2$), 32.2 (NC(*C*H_3)$_2$), 29.5 (C(*C*H_3)$_2$), 29.0 (NC(*C*H_3)$_2$), 28.6 (*C*H(CH_3)$_2$), 27.6 (*C*H(CH_3)$_2$), 26.7 (CH(*C*H_3)$_2$), 25.4 (CH(*C*H_3)$_2$), 24.5 (CH(*C*H_3)$_2$), 24.4 (CH(*C*H_3)$_2$), 17.2(PCH_2), 6.3 (PCH_2*C*H_3). Data only given for crystallised diastereomer **3c'**.

^{11}B NMR (96 MHz, 22 °C, $CDCl_3$): δ = -41.0 (br), 42.5 (br) (two diastereomers).

^{31}P NMR (122 MHz, toluene): δ = 25.2 (br, **3c'**), 21.3 (br **3c''**) (two diastereomers).

Elemental analysis for $C_{28}H_{45}BNP$ (calcd/expt): C (76.88/76.38), H (10.37/10.60), N (3.20/3.05).

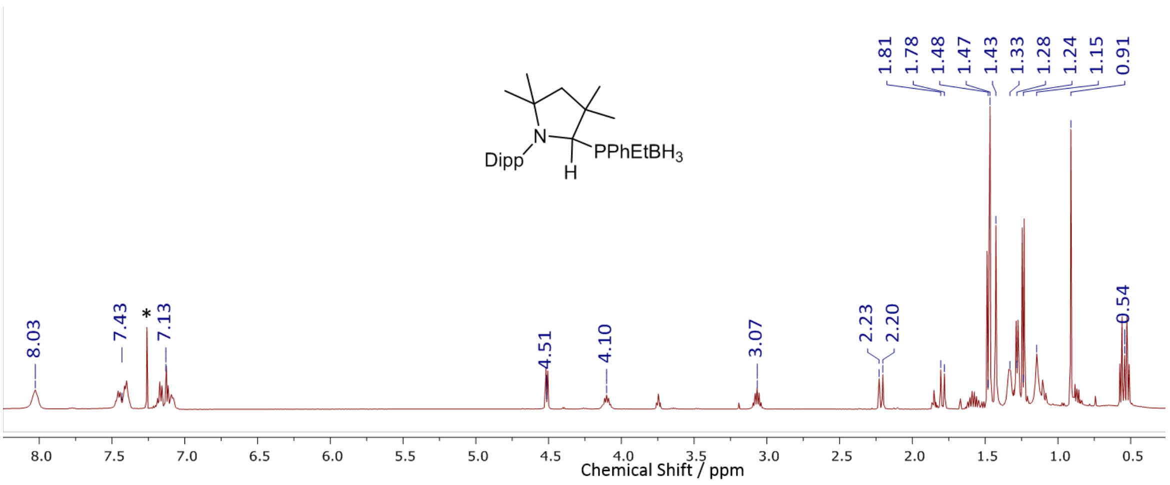

Supplementary Figure 46. ^{1}H NMR spectrum (400 MHz, 25 °C, $CDCl_3$) of **3c'** (*denotes residual partially protiated $CDCl_3$).

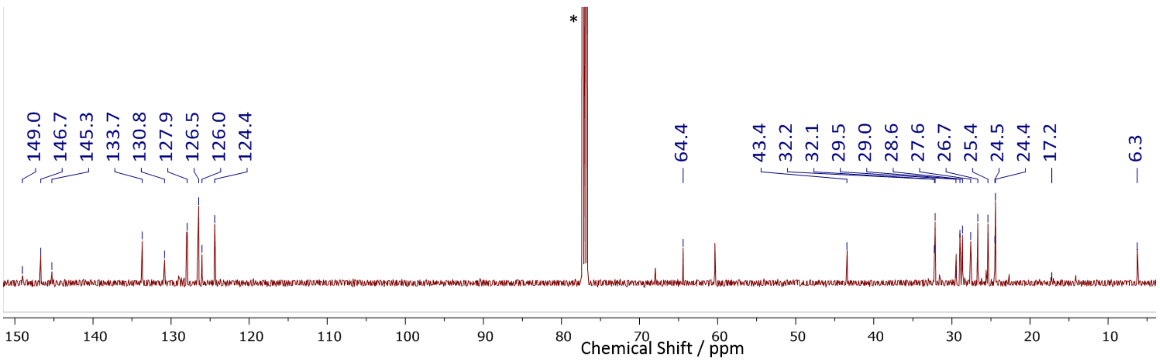

Supplementary Figure 47. ^{13}C NMR spectrum (101 MHz, 25 °C, $CDCl_3$) of **3c'** (*denotes $CDCl_3$).

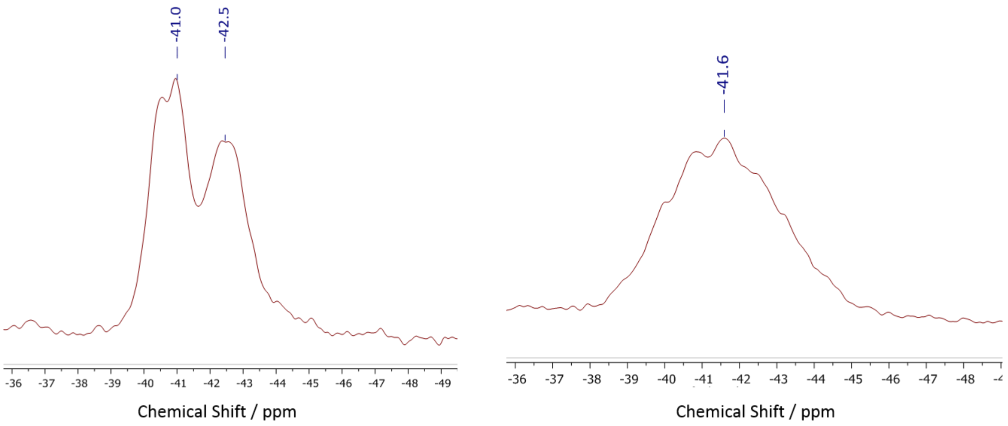

Supplementary Figure 48. $^{11}B\{^{1}H\}$ (left) and ^{11}B (right) NMR spectra (96 MHz, 22 °C, THF) of **3c**.

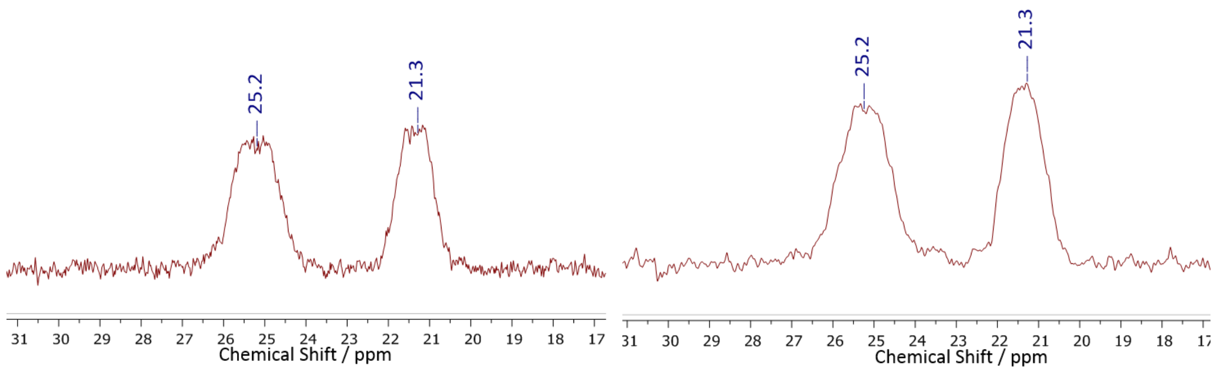

Supplementary Figure 49. $^{31}P\{^{1}H\}$ (left) and ^{31}P (right) NMR spectra (122 MHz, THF) of **3c**.

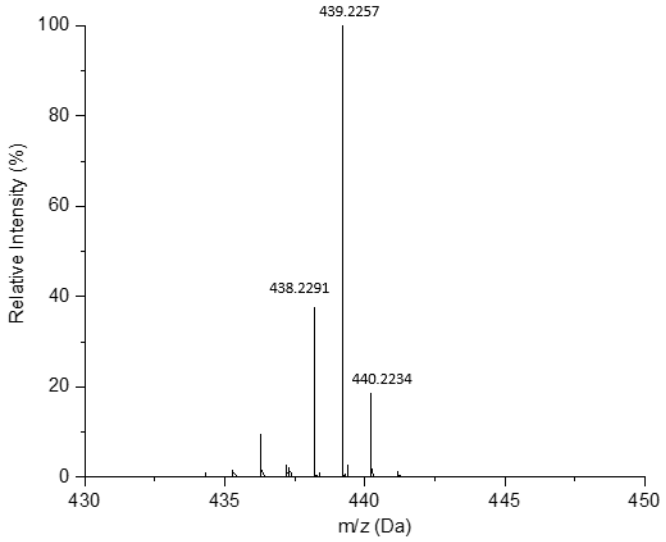

Supplementary Figure 50. ESI(+)-MS spectrum of **3c** in DCM showing the [**3c**+H]$^{+}$ peak (sample injection performed under ambient conditions in air).

Dehydropolymerisation of *rac*-PhEtPH·BH_3

Method A:

CAAC^{Me} mediated:

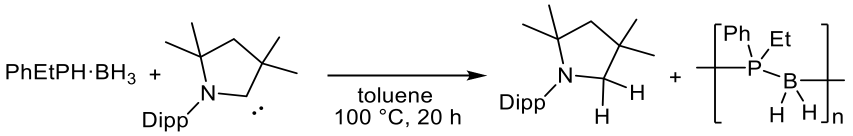

rac-PhEtPH·BH_3 (300 mg, 1.97 mmol) and CAAC^{Me} (564 mg, 1.97 mmol) were dissolved in toluene (1 mL) in a sealed J. Young Schlenk tube and heated at 100 °C for 24 hours (60°C requires ca. two weeks for full conversion). The reaction mixture was added dropwise into 20 mL of rapidly stirred cold hexanes at -40 °C yielding a precipitate and the supernatant was decanted. The precipitation was repeated twice more prior to drying in vacuo to leave a white powder. GPC analysis showed no convincing high molecular weight material. Yield (precipitated material) = 67 mg (23 %). The low yield is attributed to the loss of low molar mass material during purification.

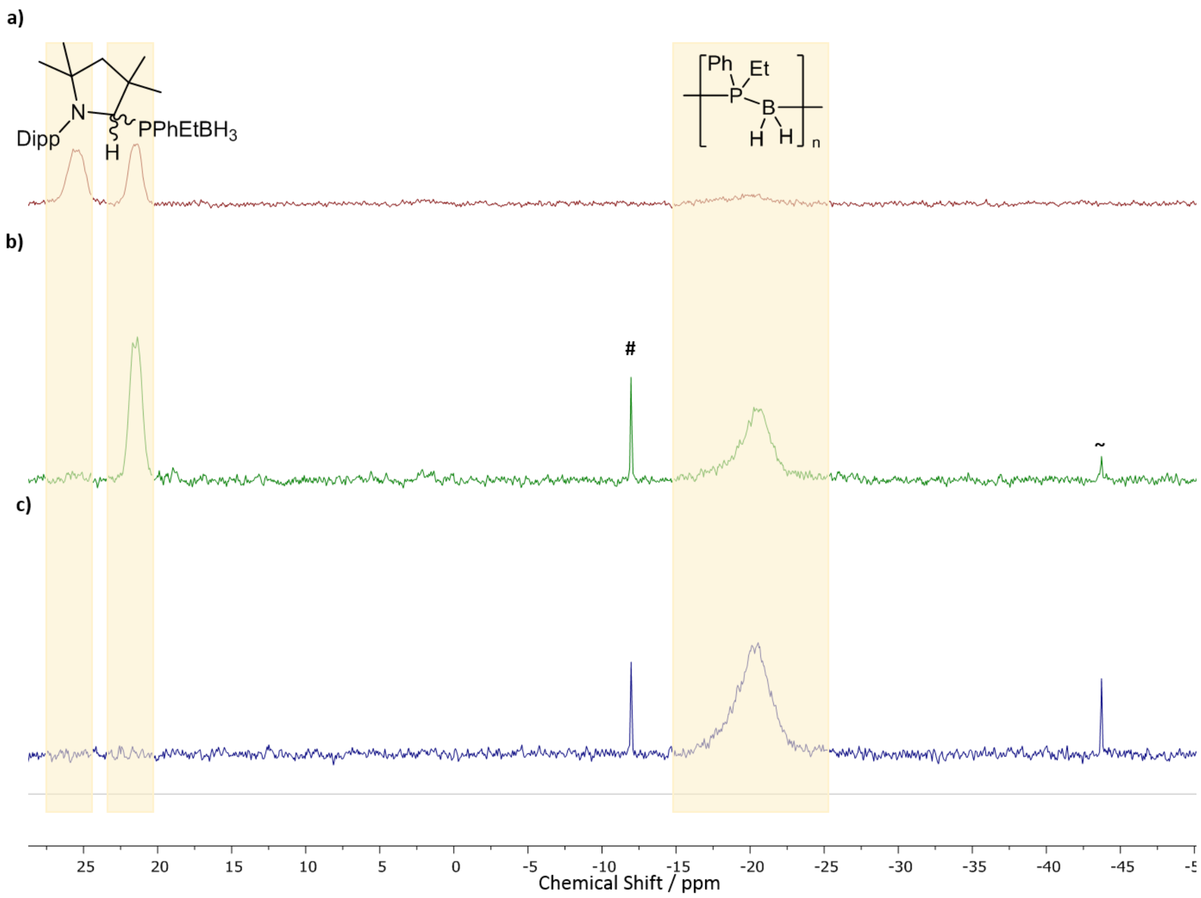

Supplementary Figure 51. $^{31}P\{^{1}H\}$ NMR spectra (122 MHz, toluene) of dehydropolymerisation of *rac*-PhEtPH·BH3 using CAAC^{Me} over time a) 10 minutes at 22 °C, b) 1 hour at 100 °C and c) 20 hours at 100 °C (# denotes trace CAAC^{Me}(H)(PhEtP), ~ denotes trace PhEtPH).

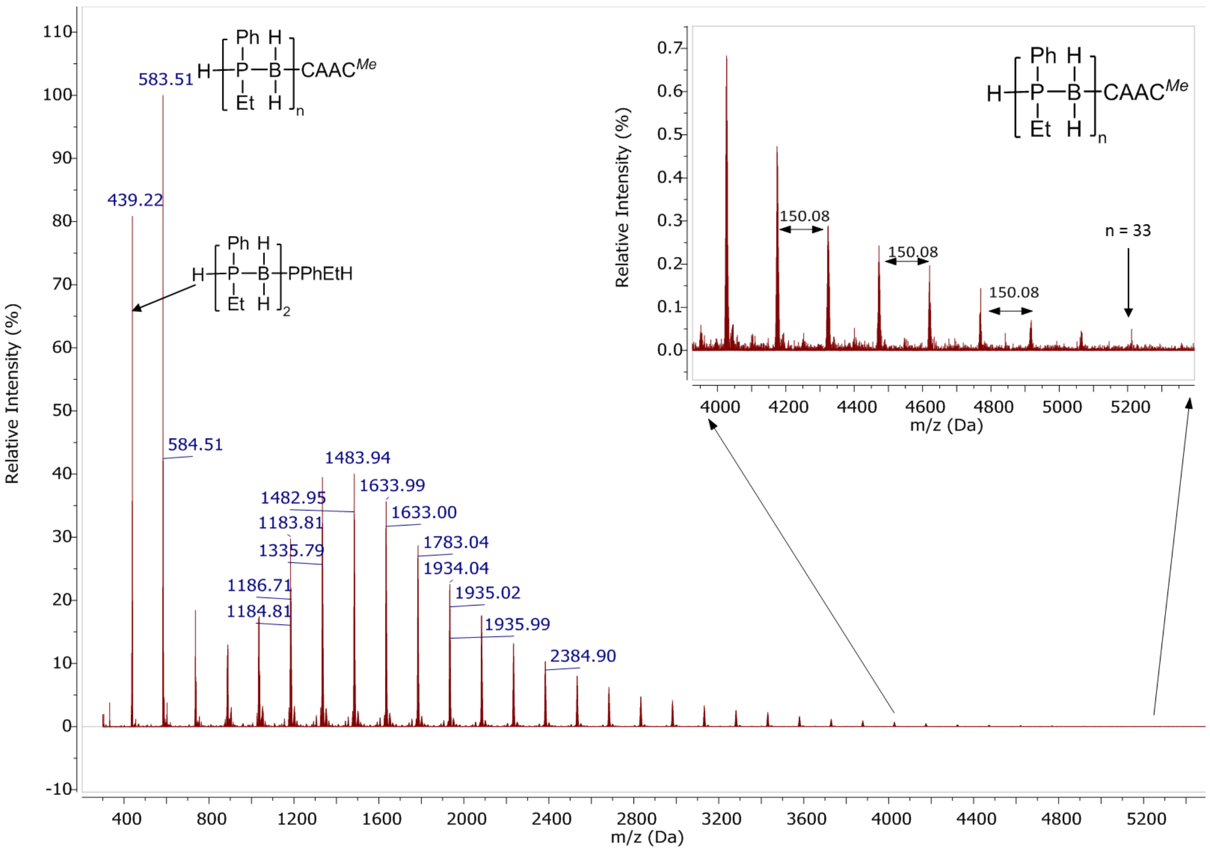

Supplementary Figure 52. ESI(+)-MS spectrum in positive mode in DCM of $[PhEtPBH_2]_n$ formed from *rac*-PhEtPH·BH_3 and CAAC^{Me} in toluene. The predominant species is a linear system with a CAAC^{Me} end group (H-$[PhEtPBH_2]_n$-CAAC^{Me})$^+$.

Method B:

CAAC^{Cy} mediated:

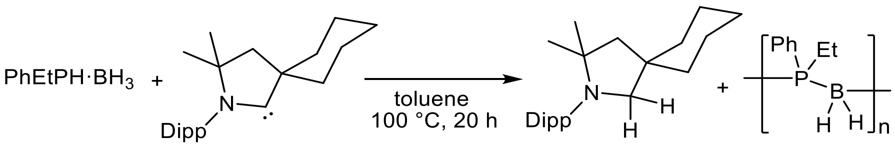

rac-PhEtPH·BH_3 (52 mg, 0.35 mmol) and CAAC^{Cy} (109 mg, 0.35 mmol) were dissolved in toluene (100 µL) in a J. Young NMR tube and heated at 100 °C for twenty hours. The reaction mixture was added dropwise into 20 mL of rapidly stirred cold hexanes at -40 °C yielding a precipitate and the supernatant was decanted. The precipitation was repeated twice more prior to drying in vacuo to leave a white powder. GPC analysis showed a bimodal distribution with 18 % corresponding to high molar mass material. Yield (precipitated material) = 10 mg (19 %).

^1H NMR (400 MHz, 25 °C, $CDCl_3$): δ = 7.75- 6.80 (m, br, Ar), 2.20 – 0.60 (m, br, BH_2, CH_2, CH_3)

^{11}B NMR (96 MHz, 22 °C, $CDCl_3$): δ = -32.8 (br)

^{31}P NMR (122 MHz, 22 °C, $CDCl_3$): δ = -21.5 (br)

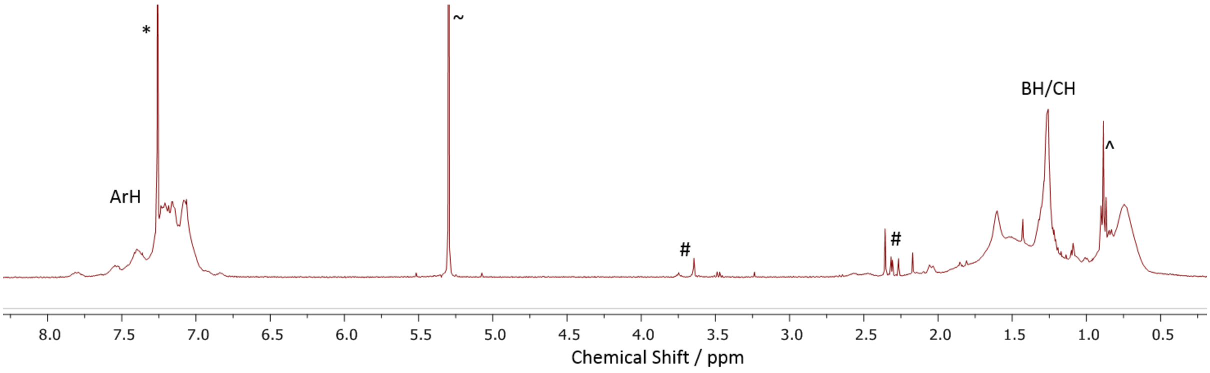

Supplementary Figure 53. ^1H NMR spectrum (400 MHz, 25 °C, $CDCl_3$) of $[PhEtPBH_2]_n$ formed from $PhEtPH{\cdot}BH_3$ and CAAC^{Cy} in toluene (* denotes residual partially protiated $CDCl_3$, ~ denotes trace DCM, # denotes trace (CAAC^{Me})H_2, ^ denotes trace hexanes).

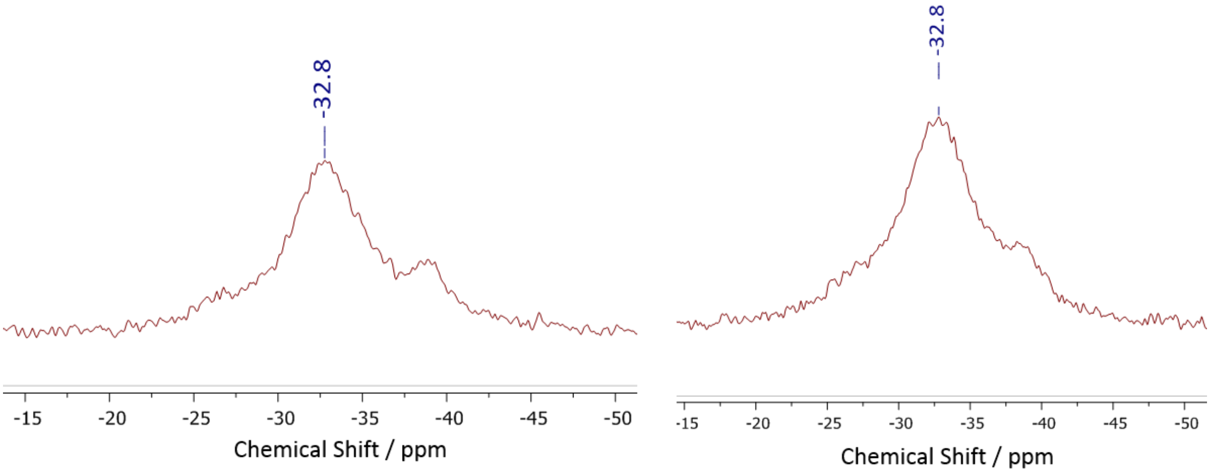

Supplementary Figure 54. $^{11}B\{^1H\}$ (left) and ^{11}B (right) NMR spectra (96 MHz, 22 °C, $CDCl_3$) of $[PhEtPBH_2]_n$ formed from $PhEtPH{\cdot}BH_3$ and CAAC^{Cy} in toluene.

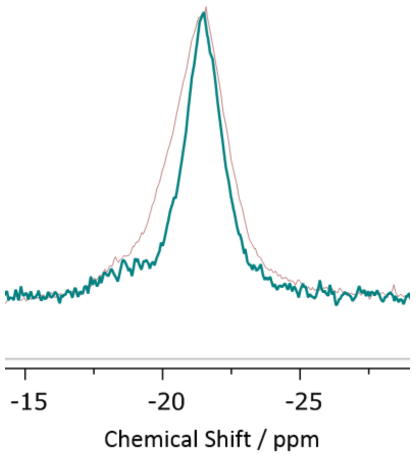

Supplementary Figure 55. The overlaid $^{31}P\{^{1}H\}$ NMR spectra before (red) and after (green) precipitation of $[PhEtPBH_2]_n$ formed from $PhEtPH{\cdot}BH_3$ and CAAC^{Cy} in toluene. Oligomeric and polymeric material formed have similar chemical shifts.

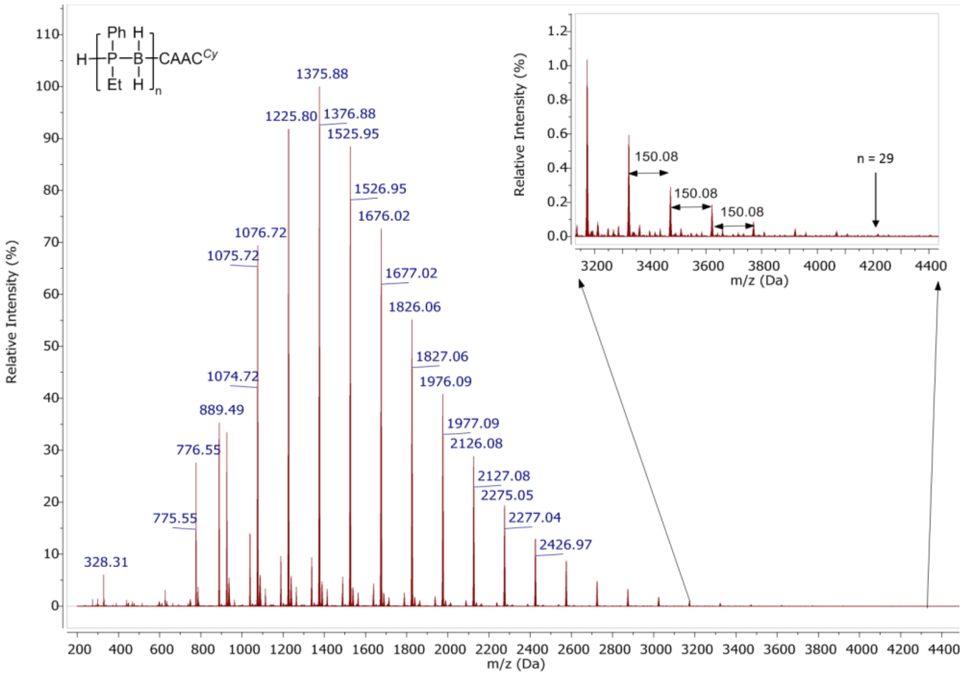

Supplementary Figure 56. ESI(+)-MS spectrum in positive mode in DCM of $[PhEtPBH_2]_n$ formed from $PhEtPH{\cdot}BH_3$ and CAAC^{Cy} in toluene The predominant species is a linear system with a CAAC^{Cy} end group (H-$[PhEtPBH_2]_n$-CAAC^{Cy})$^+$.

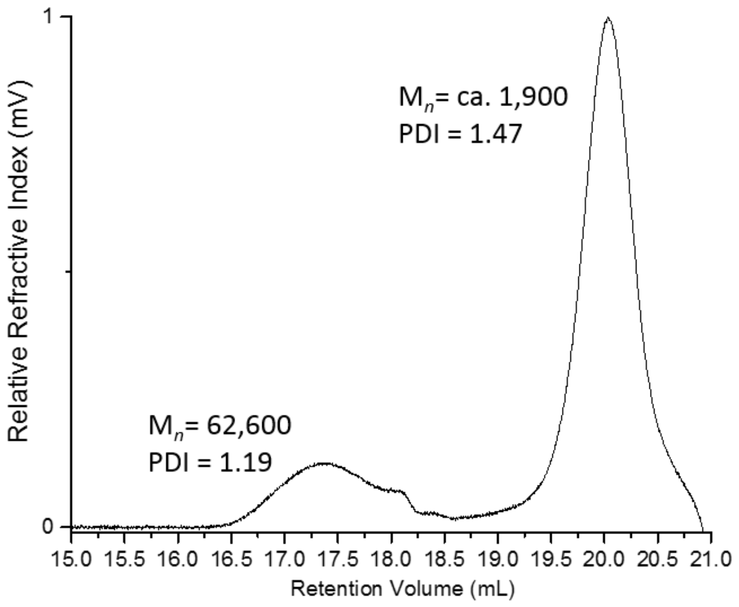

Supplementary Figure 57. GPC chromatogram of $[PhEtPBH_2]_n$ formed from $PhEtPH{\cdot}BH_3$ and CAAC^{Cy} in toluene (2 mg mL^{-1} in THF with 0.1 w/w % nBu$_4$NBr in the THF eluent). The highest molar mass peak accounts for ca. 18 % of the precipitated material. The bimodal distribution can be explained by the competition between chain termination and propagation. Most of the material undergoes early termination, potentially by a free CAAC^{Cy} unit as evidenced in the ESI-MS, whereas a small percentage of the material undergoes significant further polymerisation to give high molar mass material.

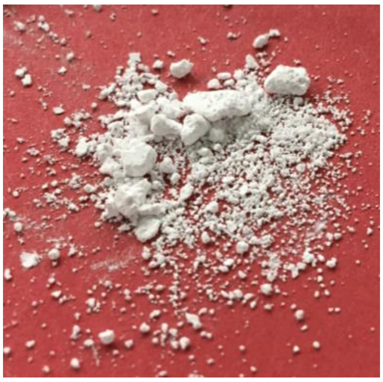

Supplementary Figure 58. Photograph of isolated $[PhEtPBH_2]_n$. Photograph taken by N. L. Oldroyd.

Synthesis of cyclic (alkyl)(amino)carbene-phosphinoborane adducts

Synthesis of **4a**

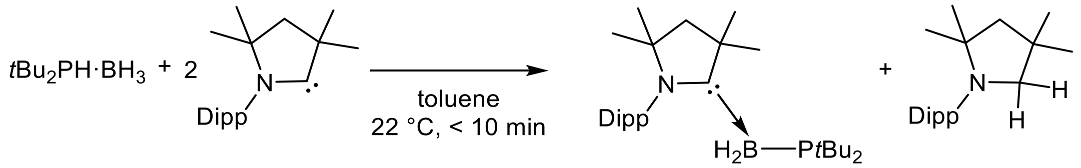

4a

5 mL of a colourless toluene solution of CAAC^{Me} (142 mg, 0.50 mmol) was added to a vial charged with colourless solid tBu$_2$PH·BH$_3$ (40 mg, 0.25 mmol) at 22 °C, instantly a bright yellow solution resulted. After 20 min at 22 °C the solvent and volatiles were removed *in vacuo* resulting in a bright yellow microcrystalline solid which was washed with 2 mL of cold *n*-pentane three times to remove the soluble (CAAC^{Me})H$_2$ byproduct. The bright yellow crystalline product **4a** was dried *in vacuo*. Yield = 57 mg (51 %). Crystals of **4a** suitable for X-ray crystallography were obtained from slow evaporation of an *n*-pentane solution at 22 °C.

^{1}H NMR (400 MHz, 25 °C, C$_6$D$_6$) δ = 7.11–7.07 (m, 1H, Dipp *p*-C*H*), 7.02 (s, 1H, Dipp *m*-C*H*), 7.00 (d, $^4J_{HH}$ = 1.2 Hz, 1H, Dipp *m*-C*H*), 2.64 (sept, $^3J_{HH}$ = 6.6 Hz, 2H, C*H*(CH$_3$)$_2$), 1.84 (d, *J* = 2.0 Hz, 6H, DippNC(C*H*$_3$)$_2$CH$_2$), 1.50 (s, 2H, Me$_2$CC*H*$_2$CMe$_2$), 1.47 (d, $^3J_{HP}$ = 10.0 Hz, 18H, P(C(C*H*$_3$)$_3$)$_2$), 1.41 (d, $^3J_{HH}$ = 6.5 Hz, 6H, -CH(C*H*$_3$)$_2$), 1.14 (d, $^3J_{HH}$ = 6.6 Hz, 6H, -CH(C*H*$_3$)$_2$), 0.83 (s, 6H, CC(C*H*$_3$)$_2$CH$_2$).

N.B. The B*H$_2$* resonances could not be observed in the ^{1}H NMR spectrum.

^{11}B NMR (128 MHz, 25 °C, C$_6$D$_6$) δ = -24.62 (td, $^1J_{BH}$ = 89.2, $^1J_{BP}$ = 42.1 Hz).

^{11}B{^{1}H} NMR (128 MHz, 25 °C, C$_6$D$_6$) δ = -24.62 (d, $^1J_{BP}$ = 42.1 Hz).

^{31}P NMR (162 MHz, C$_6$D$_6$) δ = 13.23 (br m).

^{31}P{^{1}H} NMR (162 MHz, 25 °C, C$_6$D$_6$) δ = 13.23 (m, $^1J_{BP}$ = 41.4 Hz).

^{13}C{^{1}H} NMR (101 MHz, 25 °C, C$_6$D$_6$) δ = 145.48 (Dipp *o-C*), 133.95 (Dipp *i-C*), 129.22 (Dipp *p-C*H), 125.13 (Dipp *m-C*H), 75.40 (*C(*carbene)), 53.63 (m, Me$_2$*C*CH$_2$CMe$_2$), 53.53 (Me$_2$*C*CH$_2$CMe$_2$), 32.45 (d, $^2J_{CP}$ = 12.1 Hz, P(C(*C*H$_3$)$_3$)$_2$), 31.64 (d, $^1J_{CP}$ = 21.7 Hz, P(*C*(CH$_3$)$_3$)$_2$),

30.47 (d, J = 16.7 Hz, DippNC(CH$_3$)$_2$CH$_2$), 29.15 (CH(CH$_3$)$_2$), 29.13 (CC(CH$_3$)$_2$CH$_2$), 27.60 (-CH(CH$_3$)$_2$), 24.40 (-CH(CH$_3$)$_2$).

Elemental analysis for $C_{28}H_{51}BNP$ (calcd/expt): C (75.83/75.89), H (11.59/11.65), N (3.16/3.32).

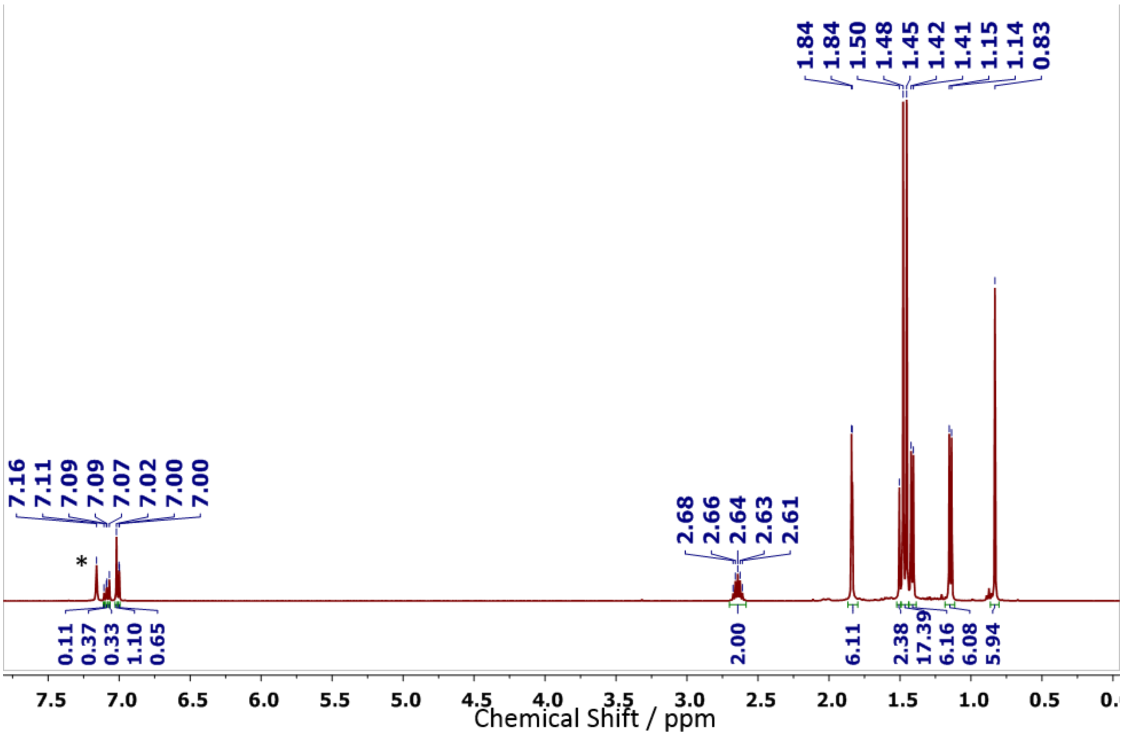

Supplementary Figure 59. ^{1}H NMR spectrum (400 MHz, 25 °C, C_6D_6) of **4a** (*denotes residual partially protiated benzene).

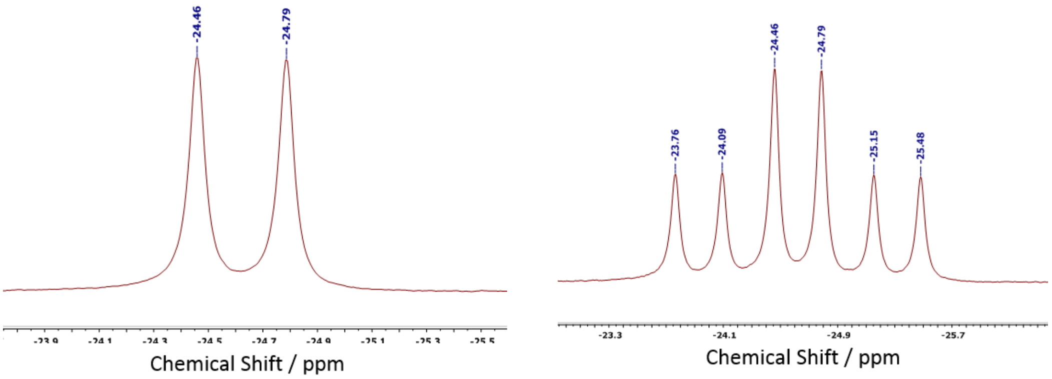

Supplementary Figure 60. ^{11}B{^{1}H} (left) and ^{11}B (right) NMR spectra (128 MHz, 25 °C, C_6D_6) of **4a**.

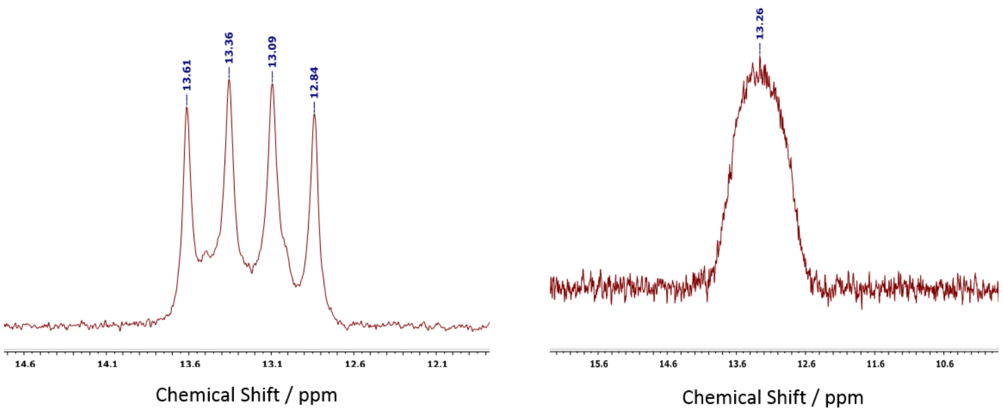

Supplementary Figure 61. $^{31}P\{^1H\}$ (left) and ^{31}P (right) NMR spectra (162 MHz, 25 °C, C_6D_6) of **4a**.

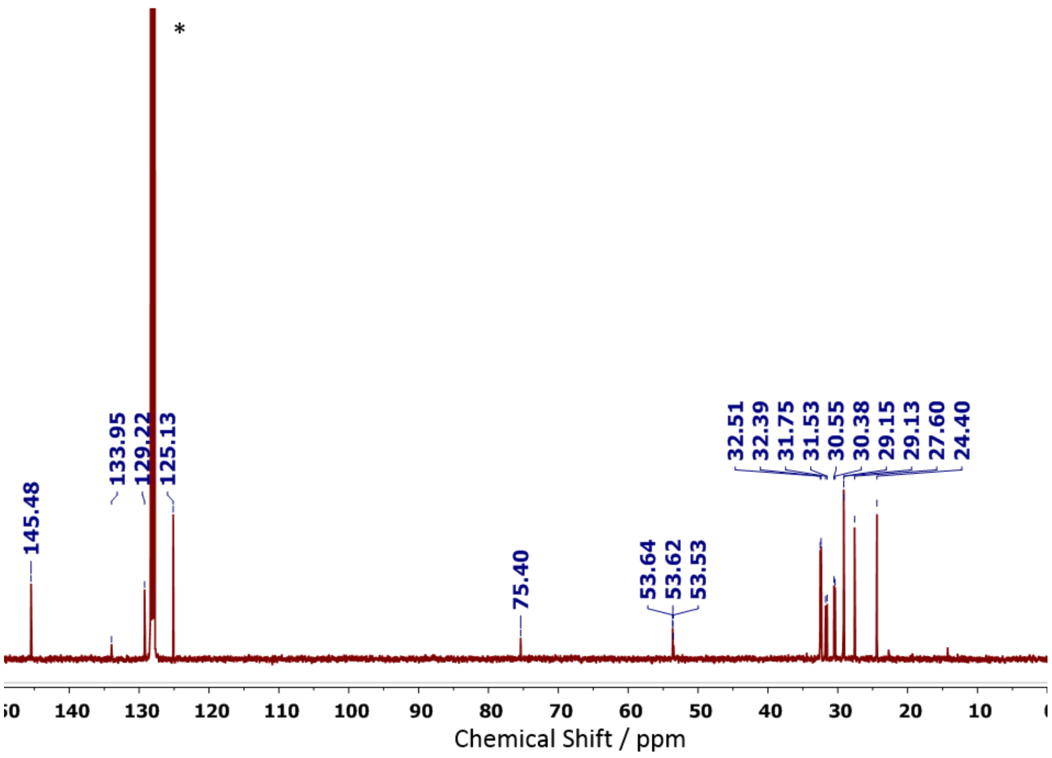

Supplementary Figure 62. $^{13}C\{^1H\}$ NMR spectrum (101 MHz, 25 °C, C_6D_6) of **4a** (*denotes benzene).

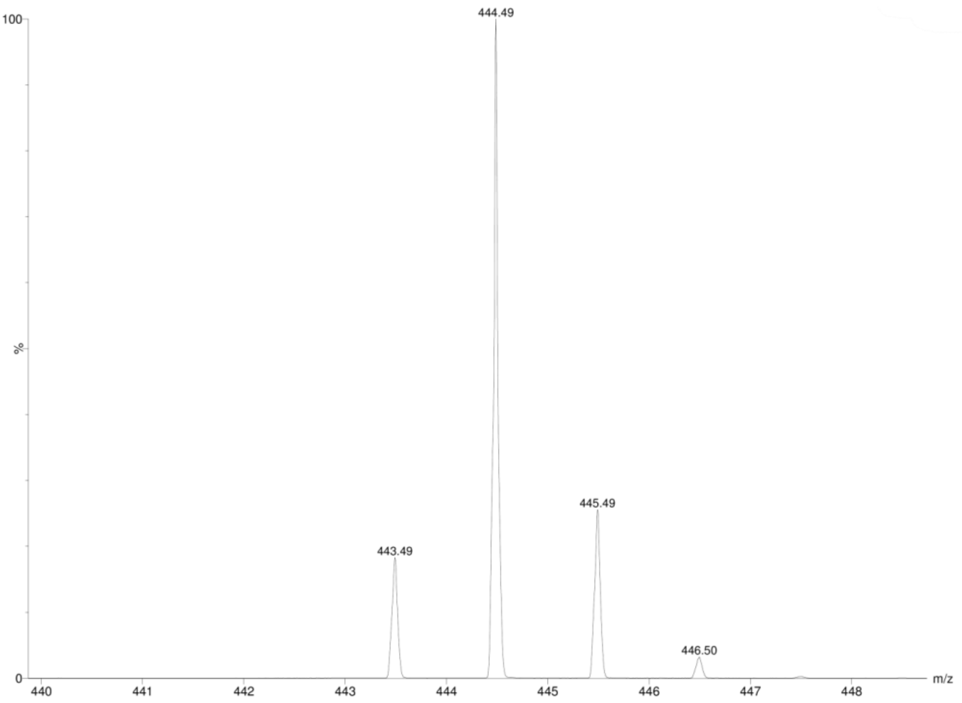

Supplementary Figure 63. ESI(+)-MS spectrum of H[**4a**]$^+$ cation in DCM (sample injection performed under ambient conditions in air).

<u>Synthesis of **4b**</u>

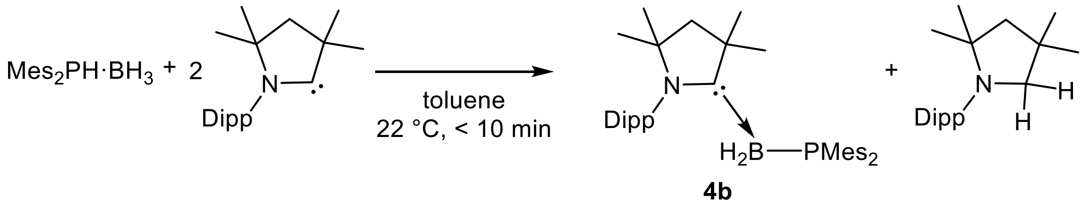

5 mL of a colourless toluene solution of CAAC^{Me} (142 mg, 0.50 mmol) was added to a vial charged with colourless solid $Mes_2PH{\cdot}BH_3$ (70 mg, 0.25 mmol) at 22 °C, instantly a yellow solution resulted. After 20 min at 22 °C the solvent and volatiles were removed *in vacuo* resulting in yellow microcrystalline solids which were washed with 2 mL of *n*-pentane three times to remove the soluble (CAAC^{Me})H_2 byproduct. The yellow crystalline product **4b** was dried *in vacuo*. Yield = 74 mg (52 %). Crystals of **4b** suitable for X-ray crystallography were obtained from diffusion of *n*-pentane into a toluene solution of the compound at -40 °C.

^{1}H NMR (400 MHz, 25 °C, C_6D_6) δ = 7.03 – 6.99 (m, 1H, Dipp *p*-C*H*), 6.92 (s, 1H, Dipp *m*-C*H*), 6.90 (m, 1H, Dipp *m*-C*H*), 6.82 (d, $^4J_{HH}$ = 1.8 Hz, 4H, Mes *m*-C*H*), 2.64 (s, 12H, Mes 2,6-(CH_3)), 2.63 (sept, $^3J_{HH}$ = 6.6 Hz, 2H, C*H*(CH_3)$_2$), 2.17 (s, 6H, Mes 4-(CH_3)), 1.79 (d, $^4J_{HH}$ = 1.8 Hz, 6H,

DippNC(CH_3)$_2$CH$_2$), 1.47 (s, 2H, Me$_2$CCH_2CMe$_2$), 1.26 (d, $^3J_{HH}$ = 6.5 Hz, 6H, -CH(CH_3)$_2$), 1.11 (d, $^3J_{HH}$ = 6.6 Hz, 6H, -CH(CH_3)$_2$), 0.82 (s, 6H, CC(CH_3)$_2$CH$_2$).

N.B. The BH_2 resonances could not be observed in the ^{1}H NMR spectrum.

^{11}B NMR (128 MHz, 25 °C, C_6D_6) δ = -21.60 (br m).

^{11}B{^{1}H} NMR (128 MHz, 25 °C, C_6D_6) δ = -21.60 (br m).

^{31}P NMR (162 MHz, 25 °C, C_6D_6) δ = -59.30 (br m).

^{31}P{^{1}H} NMR (162 MHz, 25 °C, C_6D_6) δ = -59.30 (br m).

^{13}C{^{1}H} NMR (101 MHz, 25 °C, C_6D_6) δ = 145.26 (Dipp *o-C*), 142.37 (d, J_{CP} = 11.8 Hz, Mes *i-C*), 140.45 (d, J_{CP} = 17.8 Hz, Mes *o-C*), 134.47 (Mes *p-C*), 133.31 (Dipp *i-C*), 129.45 (Dipp *p-C*H), 129.39 (d, *J* = 2.9 Hz, Mes *m-C*H), 125.30 (s, Dipp *m-C*H), 76.45 (s, *C(*carbene)), 54.09 (Me$_2$*C*CH$_2$CMe$_2$), 52.49 (m, Me$_2$C*C*H$_2$CMe$_2$), 29.53 (d, *J* = 17.0 Hz, DippNC(*C*H$_3$)$_2$CH$_2$), 29.27 (CH(*C*H$_3$)$_2$), 29.16 (CC(*C*H$_3$)$_2$CH$_2$), 27.05 (CH(*C*H$_3$)$_2$), 24.47 (CH(*C*H$_3$)$_2$), 23.97 (d, $^3J_{CP}$ = 12.2 Hz, Mes 2,6-(*C*H$_3$)), 21.05 (Mes 4-(*C*H$_3$)).

Elemental analysis for $C_{38}H_{55}BNP$ (calcd/expt): C (80.40/80.41), H (9.77/9.71), N (2.47/2.44).

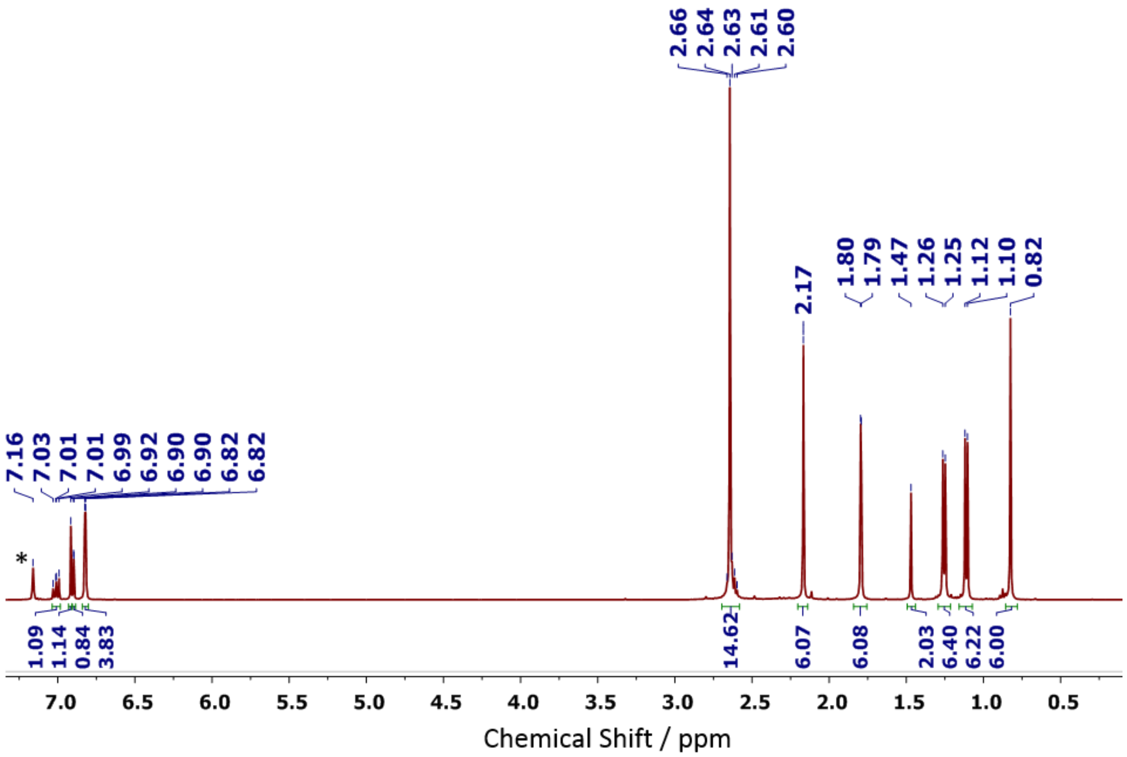

Supplementary Figure 64. ^{1}H NMR spectrum (400 MHz, 25 °C, C_6D_6) of **4b** (*denotes residual partially protiated benzene).

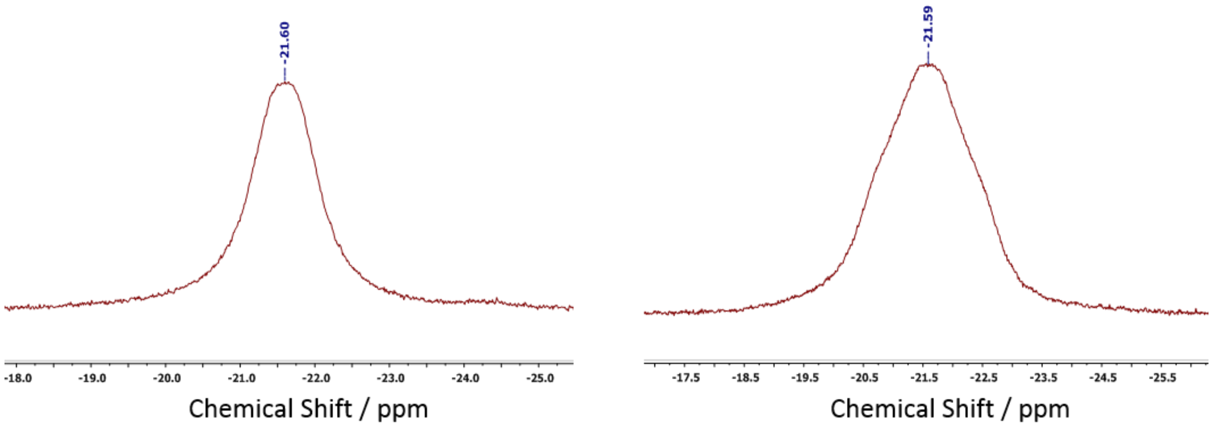

Supplementary Figure 65. $^{11}B\{^{1}H\}$ (left) and ^{11}B (right) NMR spectra (128 MHz, 25 °C, C_6D_6) of **4b**.

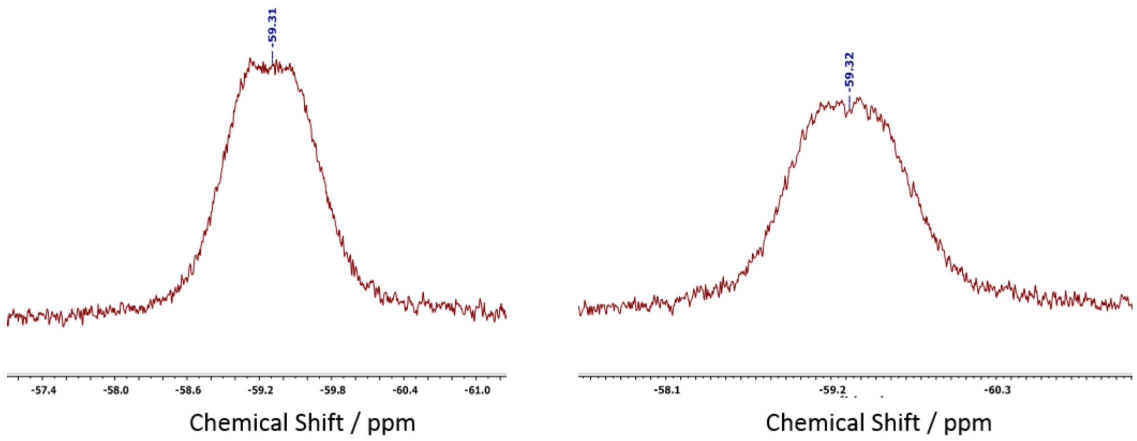

Supplementary Figure 66. $^{31}P\{^{1}H\}$ (left) and ^{31}P (right) NMR spectra (128 MHz, 25 °C, C_6D_6) of **4b**.

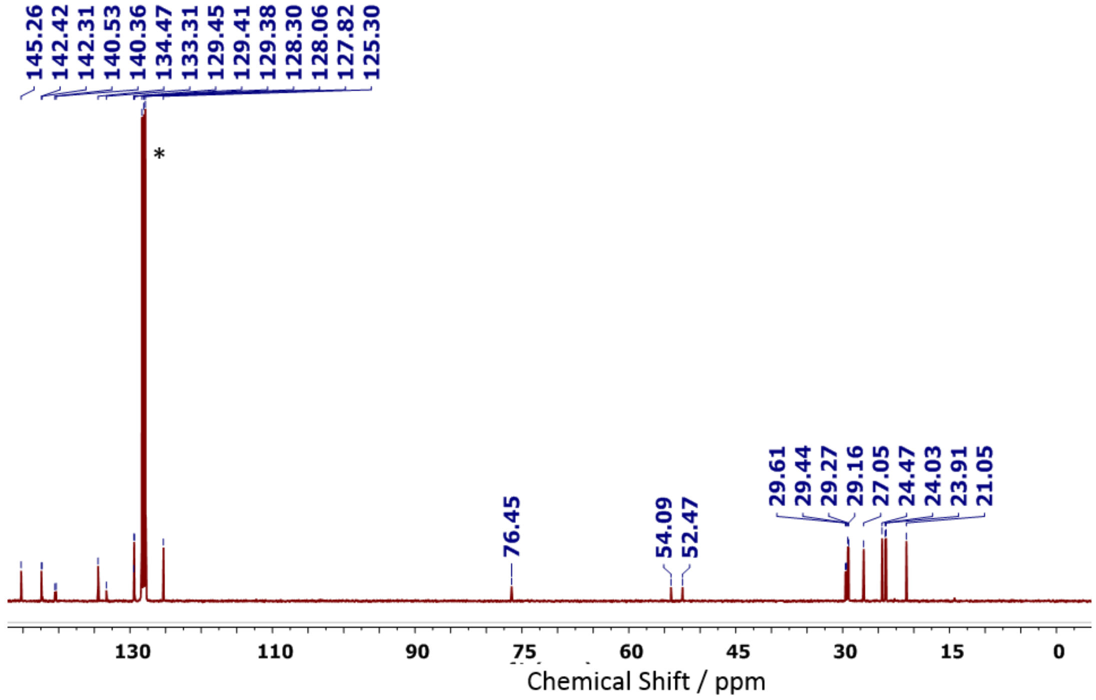

Supplementary Figure 67. $^{13}C\{^{1}H\}$ NMR spectrum (101 MHz, 25 °C, C_6D_6) of **4b** (*denotes benzene).

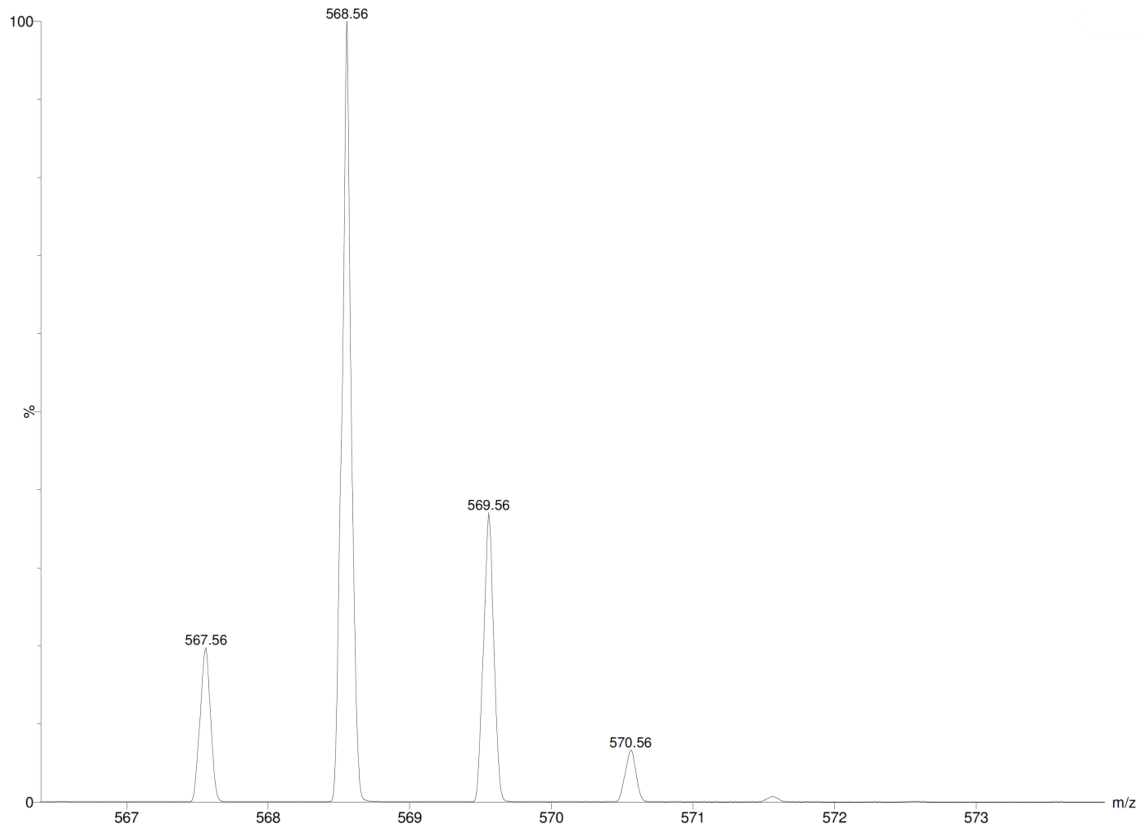

Supplementary Figure 68. ESI(+)-MS spectrum of H[**4b**]$^+$ cation in DCM (sample injection performed under ambient conditions in air).

Reaction of $Ph_2PH·BH_3$ with two equivalents of CAAC^{Me}

CAAC^{Me} (57 mg, 0.20 mmol) was added to solution of $Ph_2PH·BH_3$ (20 mg, 0.10 mmol) in thf-d_8 (0.5 mL) and heated to 60 °C for 2 hours. ^{31}P and ^{11}B NMR spectra showed a number of unidentified peaks, however ESI-MS showed the presence of short chain oligomers $CAAC(BH_2PPh_2)_x$ (x = 1 - 4).

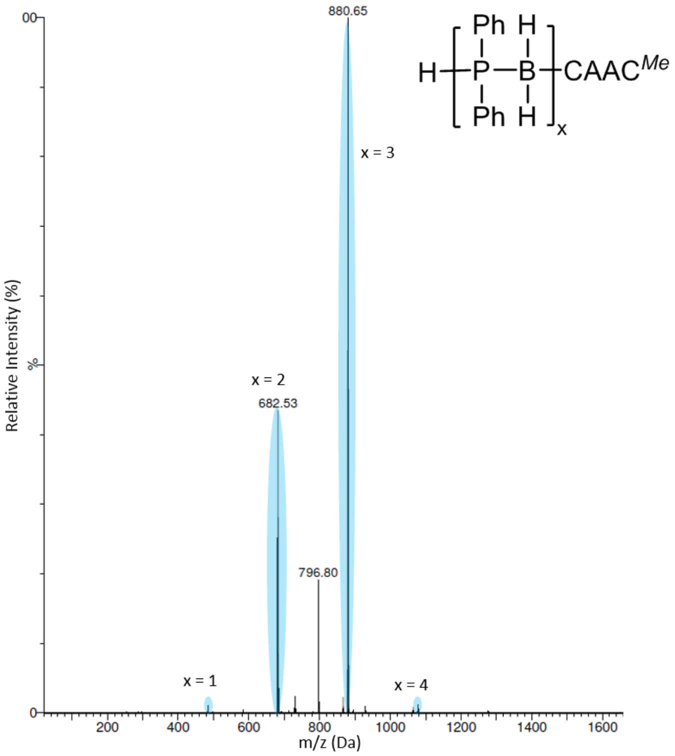

Supplementary Figure 69. ESI(+)-MS spectrum in DCM of the products from the reaction of $Ph_2PH·BH_3$ with two equivalents of CAAC^{Me} (sample injection performed under ambient conditions in air).

Supplementary Tables

X-ray crystallography

X-ray diffraction experiments on **1b**, **3c**, **4a**, **4b** and **CAAC^{Me}H$_2$** were carried out at 100(2) K on a Bruker APEX II CCD diffractometer using Mo-K$_\alpha$ radiation (λ = 0.71073 Å). Intensities were integrated in SAINT[28] and absorption corrections based on equivalent reflections were applied using SADABS[29]. The structures were solved using SHELXT[30] and refined against all F^2 in SHELXL[31] using Olex2.[32] All the non-hydrogen atoms were refined anisotropically. While all of the hydrogen atoms were located geometrically and refined using a riding model, apart from the B-H protons in **1b**, **3c**, **4a** and **4b** which were located in the difference map and refined freely. In **3c** H9 and H26 were also located in the difference map with isotropic displacement parameters Uiso(H) = 1.2Ueq(C). Disordered portions of a THF solvent molecule in the structure of **1b**·(THF) were successfully modelled over two positions and refined with the sum of the occupancies set to 1. Residual electron density from a disordered solvent molecule in the lattice of **4b** was removed using a solvent mask in Olex2, and the contributions were excluded from the formula. Crystal structure and refinement data are given in Supplementary Table 6 and Supplementary Table 7.

Crystallographic data for compounds **1b**, **3c**, **4a**, **4b** and **CAAC^{Me}H$_2$** have been deposited with the Cambridge Crystallographic Data Centre as supplementary publication CCDC 1867656-1867660. Copies of the data can be obtained free of charge on application to CCDC, 12 Union Road, Cambridge CB2 1EZ, UK [fax(+44) 1223 336033, e-mail: deposit@ccdc.cam.ac.uk].

Supplementary Table 6 Selected crystallographic data for **1b,** $(CAAC^{Me})H_2$ and **3c**.

Compound	**1b**·(THF)	$(CAAC^{Me})H_2$	**3c**
Empirical formula	$C_{43}H_{58}BN_2OP$	$C_{20}H_{33}N$	$C_{28}H_{45}BNP$
Formula weight	1321.38	287.47	437.43
Temperature/K	100(2)	100(2)	100(2)
Crystal system	triclinic	orthorhombic	monoclinic
Space group	*P*1̄	*Pbcn*	*C*c
a/Å	12.6945(5)	12.3826(4)	9.0629(4)
b/Å	16.3421(7)	12.3164(3)	19.2145(8)
c/Å	19.2329(8)	23.6194(6)	15.3373(7)
α/°	81.437(2)	90	90
β/°	87.371(2)	90	103.835(3)
γ/°	86.212(2)	90	90
Volume/$Å^3$	3934.2(3)	3602.17(17)	2593.3(2)
Z	4	8	4
ρ_{calc} g/cm^3	1.115	1.060	1.12
μ/mm^{-1}	0.104	0.060	0.121
F(000)	1432.0	1280.0	960
Crystal size/mm^3	0.48 × 0.35 × 0.3	0.35 × 0.18 × 0.10	0.60 × 0.39 × 0.21
2θ range for data collection/°	2.142 to 55.214	4.664 to 55.176	4.24 to 55.848
Reflections collected	70384	32539	23735
Independent reflections	18210	4173	5919
Goodness-of-fit on F^2	1.018	1.033	1.037
Final R indexes [I>=2σ (I)]	R_1 = 0.0431, wR_2 = 0.0959	R_1 = 0.0473, wR_2 = 0.1128	R_1 = 0.0347, wR_2 = 0.0835
Final R indexes [all data]	R_1 = 0.0626, wR_2 = 0.1045	R_1 = 0.0694, wR_2 = 0.1251	R_1 = 0.0392, wR_2 = 0.0856
Largest diff. peak/hole / e $Å^{-3}$	0.32/-0.27	0.34/-0.23	0.38/-0.19
Flack Parameter	-	-	-0.01(3)

Supplementary Table 7. Selected crystallographic data for **4a** and **4b**.

Compound	**4a**	**4b**
Empirical formula	$C_{28}H_{51}BNP$	$C_{38}H_{55}BNP$
Formula weight	443.47	567.61
Temperature/K	100(2)	100(2)
Crystal system	monoclinic	triclinic
Space group	$P2_1/n$	$P\bar{1}$
a/Å	9.3737(4)	11.4350(17)
b/Å	21.3623(8)	12.2983(18)
c/Å	14.3960(5)	14.056(2)
α/°	90	70.215(7)
β/°	97.875(2)	79.929(8)
γ/°	90	89.278(7)
Volume/Å^3	2855.52(19)	1829.1(5)
Z	4	2
ρ_{calc} g/cm^3	1.032	1.031
μ/mm^{-1}	0.111	0.099
F(000)	984.0	620.0
Crystal size/mm^3	0.48 × 0.45 × 0.4	0.5 × 0.2 × 0.15
2θ range for data collection/°	3.434 to 55.062	3.524 to 55.174
Reflections collected	27008	32259
Independent reflections	6569	8380
Goodness-of-fit on F^2	1.032	1.028
Final R indexes [I>=2σ (I)]	R_1 = 0.0388, wR_2 = 0.0969	R_1 = 0.0445, wR_2 = 0.1033
Final R indexes [all data]	R_1 = 0.0463, wR_2 = 0.1013	R_1 = 0.0672, wR_2 = 0.1121
Largest diff. peak/hole / e Å^{-3}	0.56/-0.24	0.31/-0.32

Supplementary Data

Cartesian coordinates and SCF energies of the calculated structures

A (THF); E_H = −598.95586866

C	0.97144300	-1.16398200	-0.30930900
C	2.39826300	-0.65581200	-0.18809700
C	2.29748500	0.88740000	-0.10623100
H	2.96533500	1.31146200	0.65033600
H	2.57391600	1.33033900	-1.06958900
C	0.82783600	1.20935800	0.20338800
C	2.99723300	-1.26869300	1.08739600
H	2.98693400	-2.36191200	1.03292100
H	2.44304700	-0.97107900	1.98351300
H	4.03571200	-0.93815500	1.20580500
C	3.22055000	-1.11021700	-1.39617800
H	4.24651100	-0.73025000	-1.32183600
H	2.78871400	-0.73947000	-2.33223000
H	3.26015800	-2.20289300	-1.45021300
C	0.58789500	1.57219900	1.66898200
H	1.08581700	2.52045100	1.89539200
H	0.98732100	0.80768100	2.34168600
H	-0.47923700	1.69633400	1.87855600
C	0.26573700	2.29997800	-0.70262000
H	-0.79361400	2.48917400	-0.50335700
H	0.38358300	2.03616600	-1.75825000
H	0.81211600	3.23156900	-0.52282200
C	-1.23743100	-0.26486700	-0.08382400
C	-3.27192600	-0.78786100	1.10169500
H	-3.77316100	-1.09252000	2.01591500
C	-4.01080100	-0.54767300	-0.05615200
H	-5.09098200	-0.66076800	-0.04640600
C	-3.35763100	-0.17146500	-1.22936800
H	-3.92610900	0.00569800	-2.13782500
N	0.19262100	-0.13181000	-0.09974700
C	-1.88544900	-0.64697700	1.09020600
H	-1.30046500	-0.84777200	1.98262300
C	-1.97159700	-0.02823000	-1.24538200
H	-1.45499600	0.25142100	-2.15837900

A (toluene); E_H = −598.95166567

C	0.97215500	-1.16692100	-0.30540100
C	2.39808900	-0.65565400	-0.18369900
C	2.29890800	0.88847500	-0.10510500
H	2.96303100	1.31347000	0.65464000
H	2.58186600	1.33015300	-1.06736300
C	0.82768500	1.21146400	0.19583200
C	2.99473000	-1.26623300	1.09367000
H	2.97641600	-2.35916900	1.04241300
H	2.44132800	-0.96335500	1.98867400
H	4.03510500	-0.94098500	1.21203400
C	3.22058000	-1.11491700	-1.38959000
H	4.24875900	-0.74037700	-1.31527100
H	2.79100900	-0.74474200	-2.32690700
H	3.25173400	-2.20758600	-1.44202700
C	0.58048700	1.57943500	1.65919200
H	1.07168400	2.53129400	1.88612000
H	0.98156100	0.81888300	2.33552900
H	-0.48837000	1.69709400	1.86410500
C	0.27110300	2.30106300	-0.71519800
H	-0.78992700	2.48934300	-0.52413100
H	0.39621100	2.03561000	-1.76968600
H	0.81509000	3.23407600	-0.53436600
C	-1.23534500	-0.26513100	-0.08565200
C	-3.26362900	-0.80922100	1.10113200
H	-3.75970100	-1.13206500	2.01197200
C	-4.00902200	-0.54711400	-0.04733000
H	-5.08917600	-0.66031200	-0.03368900
C	-3.36213900	-0.15131500	-1.21705500
H	-3.93545200	0.03997500	-2.11964300
N	0.19430000	-0.13097600	-0.10469500
C	-1.87762600	-0.66913300	1.08385800
H	-1.28598100	-0.89084800	1.96681000
C	-1.97641800	-0.00893200	-1.23829500
H	-1.46421600	0.28147600	-2.15027900

B (THF); E_H = −600.40015047

P	-1.82986200	-0.58525300	-0.01679800
H	-1.98888100	-1.52290400	-1.05598800
C	-0.04703000	-0.23223000	-0.01675900
C	0.89853400	-1.26507400	0.00901400
C	0.37661900	1.09956400	-0.04232400
C	2.25709300	-0.96300800	0.01016500
H	0.57748200	-2.30340900	0.02740500
C	1.73934400	1.39672100	-0.04111700
H	-0.35698900	1.90041200	-0.06367600
C	2.67792600	0.36784800	-0.01462000
H	2.98798500	-1.76608600	0.03009200
H	2.06447600	2.43270200	-0.06123500
H	3.73898400	0.60026400	-0.01381200
B	-3.02529200	0.92644500	-0.09935700
H	-4.13611100	0.43604000	-0.08992900
H	-2.76397200	1.49692700	-1.13874100
H	-2.01151400	-1.42225400	1.10192100
H	-2.78873100	1.58949600	0.88982600

B (toluene); E_H = −600.39658784

P	-1.83256300	-0.58057900	-0.01902000

H	-1.98521700	-1.52098300	-1.05855600
C	-0.04806100	-0.23309000	-0.01753500
C	0.89816700	-1.26487100	0.01081100
C	0.37487100	1.09862900	-0.04506000
C	2.25638100	-0.96286600	0.01252300
H	0.57752800	-2.30353600	0.03079400
C	1.73733000	1.39593600	-0.04326400
H	-0.36065800	1.89774100	-0.06844100
C	2.67627300	0.36796600	-0.01428200
H	2.98779700	-1.76548000	0.03444900
H	2.06202900	2.43202600	-0.06490000
H	3.73724600	0.60092000	-0.01302600
B	-3.02659000	0.93048400	-0.09704500
H	-4.13540300	0.43829300	-0.08845800
H	-2.75871900	1.49775500	-1.13566700
H	-2.00761100	-1.42691400	1.09542400
H	-2.78273800	1.58477000	0.89531600

TS1 (THF); E_H = −1199.35971457

P	-1.65668100	1.27758200	0.88668400
C	-3.13280200	0.88525100	-0.10955600
C	-3.14161500	1.07727700	-1.49877300
C	-4.25972800	0.32356800	0.50365100
C	-4.25532200	0.71966800	-2.25534600
H	-2.27651700	1.51434200	-1.99206600
C	-5.37133800	-0.03950800	-0.25638400
H	-4.26807900	0.18028500	1.58078800
C	-5.37286600	0.15803200	-1.63625700
H	-4.25109400	0.88101900	-3.32996000
H	-6.24018000	-0.47080300	0.23341600
H	-6.24122000	-0.12006100	-2.22695000
B	-2.02020800	1.54405000	2.78050100
H	-0.95154700	1.88224300	3.26124100
H	-2.37578400	0.45999100	3.21289600
H	-1.20693600	2.46109100	0.25768300
H	-2.87874800	2.39921000	2.90067800
C	0.55497700	-0.97472900	0.10898000
C	0.48248900	-2.47945000	0.18757500
C	1.91071300	-2.96633000	-0.16411100
H	2.25066200	-3.76564600	0.50113100
H	1.92741000	-3.36144100	-1.18548900
C	2.83251400	-1.73786700	-0.07288300
C	0.07149300	-2.84745300	1.62336700
H	-0.90473900	-2.41989600	1.87223300
H	0.79577600	-2.48862700	2.36116300
H	0.00377000	-3.93697100	1.71596400
C	-0.56820700	-3.00936300	-0.79247200
H	-0.60335300	-4.10372100	-0.74745800
H	-0.33409300	-2.71948200	-1.82221500
H	-1.56230700	-2.62313100	-0.54617400
C	3.65836400	-1.69515900	1.21186800
H	4.37827100	-2.51929800	1.20540400
H	3.02762900	-1.79922000	2.09939700
H	4.22041900	-0.75949100	1.29189000

C	3.74039000	-1.59493600	-1.28872500
H	4.35598100	-0.69204900	-1.23276500
H	3.16063900	-1.57167100	-2.21658200
H	4.41363200	-2.45683700	-1.33139800
C	2.19555800	0.75348100	-0.06989500
C	2.71646500	2.79575900	1.09572700
H	2.83476200	3.34059900	2.02757000
C	2.91147700	3.44095400	-0.12457900
H	3.18758100	4.49103100	-0.14629000
C	2.74182200	2.73903200	-1.31757200
H	2.88186500	3.24031200	-2.27074800
N	1.80059300	-0.62923000	-0.03628900
C	2.35885600	1.44922100	1.12685400
H	2.19035500	0.94125200	2.07160000
C	2.38311100	1.39278300	-1.29428900
H	2.23455200	0.84344300	-2.21870400
H	-0.59427400	0.19069600	0.47702100

TS1 (toluene); E_H = −1199.35332280

P	-1.56755600	1.28690000	0.72589600
C	-3.16378400	0.74906500	0.02598300
C	-3.40469900	0.80812700	-1.35443500
C	-4.14643400	0.20600500	0.86249500
C	-4.60532900	0.34187700	-1.88384900
H	-2.65147700	1.22610200	-2.01848500
C	-5.34531500	-0.26688000	0.32944000
H	-3.97166200	0.16719400	1.93424100
C	-5.57844700	-0.19952300	-1.04277400
H	-4.78212000	0.40114500	-2.95452000
H	-6.10152700	-0.68112500	0.99086700
H	-6.51508200	-0.56309700	-1.45636000
B	-1.56504700	1.45065600	2.66609200
H	-0.46546500	1.89824300	2.94291400
H	-1.70209700	0.31409400	3.08748600
H	-1.37597700	2.52415300	0.06687100
H	-2.47036500	2.18980600	3.00168100
C	0.56622500	-0.80075100	-0.36618200
C	0.37316200	-2.29198700	-0.24211000
C	1.80857400	-2.87154300	-0.16746800
H	1.90120700	-3.64566000	0.60028200
H	2.07554100	-3.32898300	-1.12644800
C	2.75013500	-1.68913600	0.11926600
C	-0.43775600	-2.55882500	1.03767100
H	-1.42436700	-2.08894800	0.98249000
H	0.06492600	-2.17826200	1.93200600
H	-0.58054900	-3.63816900	1.15988000
C	-0.40268900	-2.82216700	-1.45181200
H	-0.52203400	-3.90872800	-1.37297900
H	0.12215700	-2.60510500	-2.38825000
H	-1.39778000	-2.36985900	-1.50491100
C	3.19053700	-1.60983600	1.58038500
H	3.82763200	-2.46865100	1.81371400
H	2.33339200	-1.62543500	2.25955500
H	3.76943200	-0.70115600	1.77215600

C	3.96513500	-1.66795500	-0.80066800
H	4.59717800	-0.79365500	-0.61799800
H	3.66614300	-1.67294000	-1.85350800
H	4.56831800	-2.56245200	-0.61594100
C	2.30985800	0.82547400	-0.18372700
C	2.67802800	2.90534900	0.97058600
H	2.60089500	3.50250700	1.87417900
C	3.20833500	3.45682900	-0.19408300
H	3.55551900	4.48584900	-0.19909600
C	3.28049500	2.69049100	-1.35680200
H	3.68096500	3.12013000	-2.27033400
N	1.82627200	-0.52930800	-0.18354700
C	2.22909500	1.58614500	0.98129500
H	1.79295500	1.15919300	1.87916800
C	2.82961100	1.37270000	-1.35569600
H	2.86558900	0.77351000	-2.26028600
H	-0.50526600	0.29839700	0.03702300

TS1' (THF); E_H = −1199.35943684

C	-2.68196600	-1.73467600	-0.37096400
C	-1.43511800	-0.94720500	-0.05118900
C	-3.22138600	0.66795100	-0.10063100
C	-3.84826700	-0.73524400	-0.16924800
H	-4.59042400	-0.79812500	-0.97064000
H	-4.36412900	-0.95220000	0.77213400
C	-2.79194900	-2.94747300	0.55810400
H	-3.71364600	-3.49999500	0.34373800
H	-1.94547700	-3.62706100	0.41736500
H	-2.81515300	-2.64364600	1.60981900
C	-2.56554800	-2.21683700	-1.82644500
H	-1.68348100	-2.85162300	-1.95744500
H	-3.45271000	-2.80305000	-2.09022400
H	-2.48974000	-1.38108800	-2.52912600
C	-3.72839500	1.47959800	1.08575100
H	-3.23679200	2.45501500	1.15023700
H	-4.80242600	1.65229600	0.96472800
H	-3.57448700	0.94360100	2.02717400
C	-3.38349700	1.46414500	-1.39441100
H	-4.44397700	1.68461600	-1.55019800
H	-2.84727600	2.41680900	-1.34570300
H	-3.02048400	0.90292400	-2.26040900
N	-1.76241900	0.30379100	0.08730200
C	-0.77870100	1.32050300	0.34866200
C	-0.12093900	1.93327500	-0.71667500
C	-0.48087500	1.67175100	1.66399900
C	0.83485500	2.91419300	-0.46129700
H	-0.34843800	1.63221800	-1.73468400
C	0.47841300	2.65149000	1.91166000
H	-0.98585200	1.17107700	2.48417900
C	1.13261600	3.27713000	0.85144100
H	1.35273900	3.38736300	-1.29026500
H	0.71830600	2.91910400	2.93640700
H	1.88115200	4.03900200	1.04839100
H	2.99066500	-2.45903700	2.98456900

H	1.75051900	-0.86998100	3.20001100
H	1.00829100	-2.74041900	3.29714100
B	1.86712100	-2.02087300	2.81478700
C	2.70328800	-1.07566600	-0.09055700
C	2.78490500	-1.25341700	-1.47930800
C	3.53402000	-0.12994300	0.52184000
C	3.68214700	-0.50399500	-2.23666500
H	2.14873500	-1.98539600	-1.97157300
C	4.42801100	0.62321900	-0.23873200
H	3.48260600	0.01021100	1.59802500
C	4.50532400	0.43818700	-1.61807800
H	3.74022700	-0.65707200	-3.31097700
H	5.06947400	1.35152600	0.25018600
H	5.20591300	1.02155500	-2.20912800
P	1.48619300	-1.99866700	0.90557500
H	1.59145200	-3.29010400	0.33797900
H	0.07706700	-1.50089300	0.40472800

TS1' (toluene); E_H = −1199.35272843

C	-2.63351900	-1.72423300	-0.55007300
C	-1.39619800	-0.86556600	-0.44555800
C	-3.22243700	0.55676000	0.21702000
C	-3.75745700	-0.88110400	0.10280500
H	-4.68745500	-0.91352000	-0.47308200
H	-3.97781300	-1.26999300	1.10269800
C	-2.42045100	-3.05984700	0.16912700
H	-3.33689200	-3.65880700	0.12393300
H	-1.61617000	-3.63428600	-0.30125800
H	-2.16161100	-2.91273300	1.22274200
C	-2.88953700	-1.98639300	-2.04359300
H	-2.04057300	-2.50592300	-2.49856400
H	-3.77952800	-2.61505300	-2.15873200
H	-3.05519900	-1.05832400	-2.59971400
C	-3.44371200	1.15411300	1.60214500
H	-3.01768800	2.15852000	1.68420300
H	-4.51975800	1.22940400	1.78861400
H	-3.00821500	0.52135500	2.38132400
C	-3.77372400	1.49462000	-0.85596600
H	-4.84576400	1.64211900	-0.69259000
H	-3.28976600	2.47520400	-0.81170000
H	-3.63510900	1.08283200	-1.85995900
N	-1.74981000	0.31899400	-0.03609900
C	-0.79365400	1.38441700	0.10195100
C	-0.43540900	2.13165700	-1.01881100
C	-0.21781400	1.64446800	1.34389500
C	0.49819300	3.15712900	-0.89109800
H	-0.87671900	1.89792200	-1.98300500
C	0.71848200	2.66921500	1.46218400
H	-0.47628600	1.03135900	2.20120400
C	1.07267900	3.42951400	0.34942600
H	0.78329000	3.73598000	-1.76461100
H	1.17941200	2.86231000	2.42626100
H	1.80598500	4.22480500	0.44563100
H	2.38643500	-2.47756800	3.03629000

H	1.21166600	-0.82462400	3.01743700
H	0.36820500	-2.65325400	2.93620700
B	1.34524100	-1.98054200	2.65250700
C	2.76876000	-1.05854800	-0.03371200
C	3.04978200	-1.16059600	-1.40357000
C	3.56902100	-0.23161700	0.76295000
C	4.11323500	-0.45626300	-1.96172500
H	2.43705000	-1.79815600	-2.03678300
C	4.62985100	0.47761200	0.20101800
H	3.36251600	-0.15538600	1.82694300
C	4.90542700	0.36686100	-1.16038900
H	4.32474400	-0.55023500	-3.02357300
H	5.24694400	1.11188100	0.83188400
H	5.73603300	0.91543100	-1.59604600
P	1.34944600	-1.93170700	0.70627000
H	1.49876600	-3.22308400	0.14605700
H	0.03760200	-1.37826100	-0.04116000
C	3.72345300	-1.75490200	-1.35257400
H	4.51296800	-1.01828300	-1.18030200
H	3.22285600	-1.53309600	-2.29969700
H	4.19542700	-2.73767100	-1.44114900
C	2.59099400	0.77688400	0.03820400
C	3.27996700	2.58686600	1.45544200
H	3.34429000	3.03658400	2.44115900
C	3.78699600	3.25448800	0.34222600
H	4.25268700	4.22810400	0.46027200
C	3.68746100	2.68030600	-0.92446300
H	4.06907600	3.20554600	-1.79440100
N	1.95693000	-0.50800100	-0.11060900
C	2.67716000	1.34002900	1.30922900
H	2.27137800	0.81430100	2.16775300
C	3.08765900	1.43389000	-1.08514600
H	2.99058200	0.98826900	-2.06948900
H	0.00614400	0.19394400	0.03709900

C-1$_{pair}$ (THF); E_H = −1199.38697716

P	-1.90102800	1.79534200	0.59116600
C	-3.23497300	0.70380000	-0.04729500
C	-3.63919500	0.74232200	-1.39347700
C	-3.81546100	-0.27266800	0.77863000
C	-4.58427500	-0.15190100	-1.89132900
H	-3.20864100	1.48703200	-2.06035600
C	-4.75323900	-1.17738100	0.27908700
H	-3.53813900	-0.30889000	1.82938500
C	-5.14416400	-1.12323100	-1.05832400
H	-4.88587600	-0.09056900	-2.93436600
H	-5.18893700	-1.92032600	0.94313700
H	-5.88020000	-1.82219500	-1.44617300
B	-2.28201400	2.16529600	2.49544300
H	-1.50954800	3.06278100	2.81453200
H	-2.00583100	1.14470700	3.11954600
H	-2.29519400	2.97272900	-0.10506900
H	-3.45015300	2.48891200	2.67077800
C	0.68238100	-0.67047100	-0.07430400
C	0.25490300	-2.09216300	-0.15041400
C	1.59116400	-2.81674900	-0.44097300
H	1.71510000	-3.70087900	0.18905200
H	1.61162200	-3.14865600	-1.48311000
C	2.73296400	-1.80565400	-0.19774200
C	-0.36175100	-2.47839400	1.20767900
H	-1.25071400	-1.87763300	1.42045800
H	0.34743100	-2.35499600	2.03099700
H	-0.66017800	-3.53047100	1.16674900
C	-0.78574200	-2.27842700	-1.26228100
H	-1.05266900	-3.33794800	-1.32500600
H	-0.39210600	-1.96734200	-2.23467700
H	-1.69460600	-1.70615000	-1.05212100
C	3.45744500	-2.01456800	1.12930500
H	3.99778100	-2.96422900	1.08700500
H	2.75950700	-2.05445100	1.97029800
H	4.18579800	-1.21962800	1.31356200

C-1$_{pair}$ (toluene); E_H = −1199.37560183

P	-1.22332800	1.08417700	0.27320000
C	-2.86718300	0.28225900	0.05404900
C	-3.56967900	0.39800000	-1.15784400
C	-3.42238600	-0.52804000	1.05537500
C	-4.78233500	-0.25671800	-1.35623200
H	-3.15896400	1.01492900	-1.95505700
C	-4.62882900	-1.20062300	0.85214400
H	-2.91190900	-0.60655400	2.01182300
C	-5.31653800	-1.06786300	-0.35241900
H	-5.31162500	-0.13769000	-2.29877900
H	-5.03969500	-1.81841500	1.64745100
H	-6.26058300	-1.58344800	-0.50743800
B	-0.80592100	1.11416700	2.20520200
H	0.04791800	1.98276900	2.33983000
H	-0.32282700	0.01470400	2.48455100
H	-1.62655400	2.41080000	-0.04270800
H	-1.78879700	1.34920900	2.89038500
C	1.18689500	-1.40629100	-0.28620000
C	0.66626500	-2.79801800	-0.21383500
C	1.94400300	-3.59973200	0.12754100
H	1.76051300	-4.33976900	0.91060300
H	2.29176800	-4.13713800	-0.76051100
C	3.02039300	-2.58738600	0.57045300
C	-0.40990800	-2.87464600	0.88424800
H	-1.26312700	-2.23335600	0.64787400
H	-0.02476100	-2.57310400	1.86153000
H	-0.76241500	-3.90902500	0.95062400
C	0.05320900	-3.19720900	-1.56318200
H	-0.28321200	-4.23738600	-1.50953700
H	0.78033800	-3.11510700	-2.37730700
H	-0.81226400	-2.57210700	-1.80146100
C	3.16054800	-2.46980300	2.08699300
H	3.56493100	-3.40709900	2.47963200
H	2.19793600	-2.27959000	2.56910600
H	3.84811100	-1.66323000	2.35783100

C	4.37078300	-2.86642700	-0.07368300
H	5.13181700	-2.14706700	0.23949700
H	4.30363000	-2.87186300	-1.16586500
H	4.69991900	-3.85967600	0.24608400
C	3.08943200	-0.01921400	0.16764400
C	3.25621000	2.14305400	1.18854700
H	2.88632700	2.87231200	1.90248600
C	4.33790100	2.44281800	0.36392000
H	4.82645300	3.40971800	0.43891900
C	4.78421900	1.50947400	-0.57159500
H	5.61282300	1.74866800	-1.23104900
N	2.41875600	-1.29047400	0.06924000
C	2.62261400	0.90593800	1.09763900
H	1.76007100	0.68202800	1.72190600
C	4.16012000	0.26988500	-0.67782800
H	4.48512800	-0.44643300	-1.42433300
H	0.58701100	-0.52101500	-0.55094900

C-1’$_{\mathbf{pair}}$ (THF); E_H = −1199.38666950

C	-1.44521700	0.11390900	-1.63118400
C	-0.90034500	0.53345500	-0.31389600
C	-3.20040800	0.92176900	-0.05354500
C	-2.91993700	0.57140000	-1.53144100
H	-3.07268200	1.45860300	-2.15290400
H	-3.60736600	-0.20118500	-1.88435000
C	-1.28876400	-1.41558500	-1.74113200
H	-1.70482900	-1.73965600	-2.70009600
H	-0.23505100	-1.70858000	-1.70683000
H	-1.82050100	-1.94065100	-0.94231500
C	-0.67866500	0.79736900	-2.77038500
H	0.37765000	0.51235300	-2.76413900
H	-1.11404000	0.49112500	-3.72657600
H	-0.74250400	1.88714300	-2.69856400
C	-3.97674800	-0.16199800	0.68980400
H	-4.05815100	0.06672600	1.75634600
H	-4.98824000	-0.21350500	0.27748300
H	-3.51093400	-1.14461900	0.57457600
C	-3.87405700	2.27749200	0.10655900
H	-4.84798800	2.23616400	-0.38948100
H	-4.04423000	2.52730700	1.15737800
H	-3.28872800	3.07287500	-0.36392400
N	-1.79287100	0.96138100	0.50622300
C	-1.49686700	1.32253000	1.86848900
C	-1.44047200	2.66602200	2.23176600
C	-1.25374900	0.30776800	2.79137600
C	-1.13946600	2.99341200	3.55136700
H	-1.60725800	3.44247500	1.49307900
C	-0.95497400	0.64934200	4.10797000
H	-1.29246100	-0.73143700	2.47981300
C	-0.90096700	1.98896900	4.48845900
H	-1.08265000	4.03753700	3.84252000
H	-0.75931100	-0.13458600	4.83276500
H	-0.66438800	2.25143100	5.51505000
H	4.74220900	0.49817900	-1.61300800

H	2.92849400	0.94524800	-2.39105100
H	3.73455600	2.15338600	-1.01065000
B	3.64307200	0.99173600	-1.39403500
C	2.81841500	-1.76606000	-0.30597900
C	2.85448900	-2.71975400	0.72633200
C	2.80088100	-2.23875400	-1.62837500
C	2.87711000	-4.08504100	0.44983900
H	2.87105000	-2.38659700	1.76237100
C	2.81122900	-3.60598400	-1.90724100
H	2.79804400	-1.52259200	-2.44652900
C	2.85011000	-4.53816800	-0.87093100
H	2.91736300	-4.79912000	1.26907100
H	2.80188600	-3.94286000	-2.94127300
H	2.86690100	-5.60282800	-1.08758200
P	2.68225600	0.03304600	0.04292500
H	3.56194200	0.05815200	1.16152500
H	0.16076100	0.46931600	-0.01991900

D (THF); E_H = −599.45005436

C	1.20306100	-1.42254200	-0.33180200
C	0.66969600	-2.80629900	-0.24461700
C	1.92771900	-3.60053400	0.18205700
H	1.70581500	-4.29708600	0.99374600
H	2.29729300	-4.18609500	-0.66437600
C	3.00505900	-2.58120500	0.61402500
C	-0.44912700	-2.82773600	0.81446300
H	-1.28163500	-2.17984900	0.52498800
H	-0.08945300	-2.51525400	1.79867300
H	-0.82679200	-3.85117800	0.89756500
C	0.11023900	-3.25253300	-1.60156500
H	-0.23399200	-4.28767200	-1.51940100
H	0.87163400	-3.20715300	-2.38549600
H	-0.74071600	-2.63408700	-1.90189300
C	3.15016300	-2.45241600	2.12779400
H	3.55161800	-3.39072400	2.51979800
H	2.18874500	-2.26374200	2.61344700
H	3.84451400	-1.65086100	2.39499600
C	4.35297700	-2.84502400	-0.04148000
H	5.10147600	-2.10325100	0.24922000
H	4.27187300	-2.87136900	-1.13203200
H	4.70591700	-3.82469500	0.29327400
C	3.07722800	-0.01558500	0.17812700
C	3.37242400	2.08636100	1.29662800
H	3.11026000	2.77082700	2.09710200
C	4.34202800	2.43795500	0.35932000
H	4.83906000	3.40051100	0.42949100
C	4.67115300	1.55968300	-0.67241800
H	5.41783000	1.83731600	-1.40955600
N	2.40723800	-1.28792300	0.09176200
C	2.73168200	0.85271700	1.21130300
H	1.97602000	0.56596400	1.93612400
C	4.03996200	0.32234400	-0.77024700
H	4.27972900	-0.35947200	-1.57908600
H	0.65174200	-0.55766600	-0.69495400

D (toluene); E_H = −599.43047691

C	1.20458100	-1.42266800	-0.32825300
C	0.66911900	-2.80578100	-0.24246300
C	1.92929400	-3.60158900	0.17592200
H	1.70997100	-4.30377700	0.98361900
H	2.29661000	-4.18249900	-0.67493400
C	3.00744700	-2.58401300	0.61093700
C	-0.44526400	-2.83140000	0.82139200
H	-1.27940500	-2.18131500	0.54113000
H	-0.08145300	-2.52752800	1.80696200
H	-0.82611200	-3.85398600	0.90005200
C	0.10324700	-3.24579900	-1.59913000
H	-0.24174000	-4.28098600	-1.52180300
H	0.85985800	-3.19809200	-2.38782700
H	-0.74973400	-2.62716200	-1.89368500
C	3.14797100	-2.45263100	2.12507300
H	3.54727900	-3.38957800	2.52254100
H	2.18506200	-2.26206700	2.60737800
H	3.84163000	-1.65097600	2.39353700
C	4.35686500	-2.84956500	-0.04053100
H	5.10607500	-2.10915800	0.25140300
H	4.28009500	-2.87726200	-1.13152400
H	4.70885100	-3.82949500	0.29462100
C	3.08075600	-0.01579100	0.17583800
C	3.36253400	2.09078600	1.28888600
H	3.09359800	2.77794800	2.08469600
C	4.33842300	2.44048700	0.35774200
H	4.83271000	3.40429500	0.42795900
C	4.67795700	1.55920900	-0.66775600
H	5.42969900	1.83631000	-1.39983000
N	2.41129000	-1.28898200	0.08873000
C	2.72519700	0.85563000	1.20312600
H	1.96676700	0.56868100	1.92539900
C	4.05073100	0.32010900	-0.76591400
H	4.29917900	-0.36397300	-1.57041400
H	0.65335000	-0.55565400	-0.68790000

E (THF); E_H = −599.91947563

P	-1.39385600	0.91193500	0.75408000
C	-3.05943300	0.55339700	0.06430100
C	-3.39655800	0.84655500	-1.26892200
C	-4.02331800	-0.10746700	0.84456200
C	-4.63799600	0.49796000	-1.79740100
H	-2.67494100	1.35859500	-1.90258100
C	-5.26314100	-0.46531300	0.31593100
H	-3.79793500	-0.33280800	1.88402200
C	-5.58073600	-0.16466000	-1.00896600
H	-4.87096800	0.74624400	-2.83043200
H	-5.98840600	-0.97460800	0.94656800
H	-6.54864000	-0.43834100	-1.42032100
B	-1.67197300	1.59038800	2.59619600
H	-0.58985000	2.05787300	2.94105600
H	-1.94345400	0.62453700	3.30321300
H	-1.16884600	2.09622300	-0.00791800
H	-2.56080100	2.43573000	2.64179000

E (toluene); E_H = −599.89434458

P	-1.39572200	0.99952800	0.71798200
C	-3.05965600	0.60257000	0.04244100
C	-3.38820800	0.81714000	-1.30821200
C	-4.02988700	-0.01312900	0.85092500
C	-4.62539300	0.44087100	-1.82530100
H	-2.65840100	1.28904000	-1.96371400
C	-5.26450400	-0.40351300	0.33331700
H	-3.81058900	-0.16621200	1.90464800
C	-5.57371100	-0.17870100	-1.00819200
H	-4.85112400	0.63029700	-2.87281500
H	-5.99502300	-0.87520300	0.98745300
H	-6.53881200	-0.47618700	-1.41063200
B	-1.66316100	1.51780600	2.61381900
H	-0.60000600	2.01971400	2.96720600
H	-1.85712900	0.48318300	3.24680900
H	-1.26125700	2.24780000	0.03863400
H	-2.59826800	2.30123700	2.74081100

F (THF); E_H = −1199.42894789

C	0.11742900	-1.84851500	-0.99847200
C	-0.05712800	-0.43317100	-0.40967200
C	-2.10509900	-1.67414100	0.09544500
C	-0.92384600	-2.62563300	-0.18730100
H	-1.25849600	-3.53378000	-0.69870400
H	-0.47150600	-2.92975600	0.76412400
C	1.51202800	-2.45242200	-0.85287100
H	1.52127000	-3.45142700	-1.30249300
H	2.26616600	-1.85020300	-1.36979600
H	1.80871600	-2.56031400	0.19439400
C	-0.22086100	-1.77882600	-2.49566500
H	0.60262800	-1.30955900	-3.04527600
H	-0.36758400	-2.78513900	-2.90267800
H	-1.12375600	-1.19493900	-2.69168700
C	-2.61547700	-1.94331600	1.51903600
H	-3.55928300	-1.44386400	1.74820300
H	-2.78875000	-3.01958800	1.62858100
H	-1.87112200	-1.64301000	2.26021300
C	-3.24453100	-1.89899300	-0.90929500
H	-3.67117500	-2.89340200	-0.74227600
H	-4.05019600	-1.17061500	-0.79432300
H	-2.89384100	-1.85243900	-1.94180600
N	-1.46247500	-0.33686300	-0.07423400
P	1.00943800	0.02012200	1.09124600
H	0.40911900	1.25871600	1.39559900
B	1.12830400	-1.08315200	2.68176100
H	1.83902900	-0.41064100	3.40540100

H	0.00283700	-1.18355000	3.11701300
H	1.62810400	-2.14856100	2.38846300
C	2.62924000	0.53439000	0.43677200
C	2.74753800	1.62787800	-0.43111000
C	3.77730500	-0.17480700	0.80748600
C	3.99445900	1.99777200	-0.92932500
H	1.87164900	2.20737500	-0.71197300
C	5.02336600	0.19674900	0.30699700
H	3.69450300	-1.01406000	1.49222900
C	5.13320400	1.28041900	-0.56317700
H	4.07666500	2.84970500	-1.59800700
H	5.90911600	-0.35861600	0.60152400
H	6.10557900	1.57078900	-0.95063300
C	-2.11786200	0.88858600	-0.04670000
C	-1.49352300	2.06808900	-0.51239100
C	-3.43193100	1.02962600	0.45042200
C	-2.14755500	3.29681200	-0.49090500
H	-0.48788400	2.04395800	-0.91283600
C	-4.07620000	2.26137400	0.46149700
H	-3.96229100	0.17528200	0.84418600
C	-3.44741700	3.41382500	-0.00718100
H	-1.62198600	4.17207400	-0.86398700
H	-5.08832900	2.31402000	0.85438000
H	-3.95390000	4.37374400	0.00963800
H	0.22686000	0.30266500	-1.17092500

F (toluene); $E_H = -1199.42469202$

C	0.11604700	-1.85333900	-0.99712400
C	-0.05402900	-0.43819000	-0.40691200
C	-2.10535600	-1.67166300	0.09743400
C	-0.92620400	-2.62705100	-0.18355000
H	-1.26356700	-3.53549300	-0.69321600
H	-0.47365100	-2.92974200	0.76814900
C	1.50986200	-2.46000600	-0.85513500
H	1.51822400	-3.45725500	-1.30892800
H	2.26466000	-1.85670200	-1.37015500
H	1.80700400	-2.57202100	0.19134200
C	-0.22594800	-1.78143100	-2.49341600
H	0.59848300	-1.31652200	-3.04559900
H	-0.37834600	-2.78652900	-2.90174000
H	-1.12650400	-1.19288400	-2.68655800
C	-2.61394600	-1.93410700	1.52296600
H	-3.55640800	-1.43155600	1.75110700
H	-2.78911000	-3.00958100	1.63808100
H	-1.86852500	-1.63257800	2.26240900
C	-3.24670100	-1.89764100	-0.90515400
H	-3.68274800	-2.88637300	-0.72816500
H	-4.04532100	-1.16025100	-0.79893500
H	-2.89584900	-1.86464200	-1.93819500
N	-1.46052100	-0.33730500	-0.07903800
P	1.00689700	0.00440400	1.10136700
H	0.39881500	1.23753600	1.41406300
B	1.13994400	-1.11857800	2.67594500
H	1.84301300	-0.44417500	3.40364300

H	0.01638900	-1.23767400	3.10888200
H	1.65312500	-2.17167400	2.36328000
C	2.62087200	0.53509100	0.44337500
C	2.72942100	1.62691100	-0.42754100
C	3.77551600	-0.16177700	0.81585700
C	3.97209500	2.00685700	-0.92806200
H	1.84748500	2.19818300	-0.70716900
C	5.01748800	0.21936700	0.31279600
H	3.69907800	-0.99698800	1.50631100
C	5.11717100	1.30063000	-0.56099900
H	4.04661100	2.85818400	-1.59857600
H	5.90862400	-0.32623100	0.60948500
H	6.08671900	1.59866400	-0.94986900
C	-2.11144000	0.89083000	-0.05221700
C	-1.48282900	2.06690600	-0.51950600
C	-3.42451700	1.03753400	0.44425800
C	-2.13258900	3.29762700	-0.50131800
H	-0.47665700	2.03717400	-0.91851900
C	-4.06434200	2.27106800	0.45218700
H	-3.95609300	0.18568700	0.84168900
C	-3.43179000	3.41986100	-0.01895000
H	-1.60440400	4.17082400	-0.87557500
H	-5.07598300	2.32868100	0.84548500
H	-3.93489300	4.38146600	-0.00421400
H	0.23527200	0.29713900	-1.16686400

F’ (THF); $E_H = -1199.42684957$

C	0.24568100	2.37301400	-0.16531900
C	-0.23417100	1.08534900	0.57985900
C	-1.78724400	1.35891100	-1.19265300
C	-0.71630100	2.43875100	-1.37595400
H	-1.16861600	3.43021900	-1.48093000
H	-0.15910200	2.23460900	-2.29662100
C	1.68646400	2.31225700	-0.68002500
H	1.88761300	3.20576900	-1.28177600
H	2.41470700	2.29631100	0.13738200
H	1.85163700	1.43780500	-1.31599600
C	0.10359700	3.58857100	0.75576100
H	0.73442200	3.48075300	1.64445700
H	0.41219900	4.49834000	0.22933900
H	-0.93118000	3.72743800	1.08682200
C	-2.23282200	0.79632000	-2.53741500
H	-3.02389900	0.04879300	-2.42553500
H	-2.63065700	1.60937300	-3.15402000
H	-1.38952400	0.34327100	-3.06799100
C	-3.00953500	1.90233100	-0.43971300
H	-3.48606900	2.69257000	-1.02880200
H	-3.74957000	1.11379800	-0.27012600
H	-2.74084500	2.33008100	0.53147600
N	-1.03784300	0.34427800	-0.38438100
P	1.07343200	0.06979500	1.44290600
C	-1.79436900	-0.77076000	0.08248000
C	-2.34164800	-0.84514400	1.37144500
C	-1.99799800	-1.85106500	-0.78770700

C	-3.06677200	-1.96548200	1.77419700
H	-2.21039000	-0.02651500	2.07276000
C	-2.73807900	-2.96187800	-0.39001000
H	-1.55966100	-1.81507000	-1.78029200
C	-3.27357000	-3.02737100	0.89578600
H	-3.47623100	-2.00200400	2.78003500
H	-2.88124100	-3.78586300	-1.08368900
H	-3.83918300	-3.89898600	1.21188600
H	0.28544600	-0.99937100	1.91028500
H	2.56154500	0.12613800	3.49481300
H	2.61594400	1.87495100	2.47392300
H	0.99421200	1.40149800	3.58758800
B	1.92172500	0.98485700	2.92005700
C	2.19748400	-0.76711700	0.28368100
C	1.73034200	-1.69687300	-0.65348600
C	3.56916600	-0.50294000	0.37095300
C	2.62684300	-2.33963300	-1.50423700
H	0.67034900	-1.92092700	-0.71695600
C	4.46217600	-1.14930600	-0.48082700
H	3.93600800	0.20682600	1.10720100
C	3.99175100	-2.06477400	-1.42140600
H	2.25844100	-3.05975400	-2.22933700
H	5.52536400	-0.93980800	-0.40651700
H	4.68868400	-2.56945700	-2.08456100
H	-0.82573200	1.42051200	1.45236100

F’ (toluene); E_H = −1199.42294119

C	0.24731800	2.36638500	-0.16989200
C	-0.23299400	1.08030300	0.57765400
C	-1.78906700	1.35386000	-1.19227500
C	-0.71645300	2.43156600	-1.37914900
H	-1.16713900	3.42387900	-1.48536100
H	-0.16107700	2.22446900	-2.30033000
C	1.68754100	2.30360800	-0.68612400
H	1.88911900	3.19630300	-1.28918600
H	2.41594500	2.28852500	0.13094400
H	1.85176000	1.42793000	-1.32054700
C	0.10829500	3.58283000	0.75061800
H	0.73715900	3.47295600	1.64024500
H	0.42050900	4.49176800	0.22464700
H	-0.92661100	3.72558000	1.08016200
C	-2.23830900	0.79068700	-2.53574500
H	-3.03135400	0.04555700	-2.42182600
H	-2.63461400	1.60348300	-3.15381500
H	-1.39642000	0.33479500	-3.06618800
C	-3.00949800	1.90039400	-0.43817400
H	-3.48855700	2.68863000	-1.02808900
H	-3.74884300	1.11251300	-0.26278600
H	-2.73752300	2.33194700	0.53041900
N	-1.04056900	0.33973400	-0.38423100
P	1.07540900	0.06948500	1.44490300
C	-1.79739400	-0.77320400	0.08550000
C	-2.33733000	-0.84769200	1.37744800
C	-2.00883000	-1.85268700	-0.78368700

C	-3.06304700	-1.96606300	1.78337700
H	-2.19830100	-0.03022500	2.07869600
C	-2.74960900	-2.96144600	-0.38269400
H	-1.57525900	-1.81674300	-1.77838600
C	-3.27798300	-3.02637400	0.90563700
H	-3.46610500	-2.00264600	2.79177100
H	-2.89879800	-3.78484900	-1.07582600
H	-3.84383200	-3.89675500	1.22450900
H	0.28779000	-1.00237600	1.90810600
H	2.55661700	0.14021200	3.49773600
H	2.60643700	1.88323100	2.46404100
H	0.98280700	1.40970600	3.57680500
B	1.91668600	0.99409600	2.91828300
C	2.19949200	-0.76342600	0.28224400
C	1.73320200	-1.66398000	-0.68313200
C	3.57320000	-0.52328900	0.39785100
C	2.63338200	-2.30185200	-1.53316900
H	0.67069500	-1.86724600	-0.76871300
C	4.46966700	-1.16474900	-0.45382000
H	3.93699100	0.16295200	1.15764500
C	4.00071500	-2.05102600	-1.42204200
H	2.26614700	-2.99999700	-2.28018300
H	5.53472000	-0.97449000	-0.35718300
H	4.70052500	-2.55224300	-2.08493200
H	-0.82363500	1.41936000	1.44941100

TS4 (THF); E_H = −1199.39272398

C	0.19480000	2.22889700	-0.49459400
C	0.50814500	0.76616300	-0.62230500
C	2.27930100	1.57534600	0.70679800
C	1.15906100	2.63853200	0.63871200
H	1.57038100	3.63960300	0.48240500
H	0.61169600	2.64662700	1.58623900
C	-1.26088200	2.56797500	-0.19347700
H	-1.38590700	3.65594100	-0.19453200
H	-1.92925400	2.14971600	-0.95254900
H	-1.56523200	2.18277900	0.78261100
C	0.58289400	2.86199800	-1.84969100
H	-0.08021400	2.51175000	-2.64711900
H	0.48150500	3.94953100	-1.77635100
H	1.61319800	2.63429800	-2.13646400
C	2.49795600	1.08850700	2.13634900
H	3.28994300	0.33589800	2.19253400
H	2.80015700	1.93991500	2.75397500
H	1.57365700	0.67540300	2.55191200
C	3.59971100	2.03484200	0.09304700
H	4.01494100	2.84199400	0.70346400
H	4.32833700	1.21850800	0.07395500
H	3.46951500	2.40947800	-0.92569400
N	1.69476000	0.48309600	-0.13980700
P	-1.38800400	-0.60105200	1.08925000
H	-0.96106800	-1.95291100	1.00273600
B	-1.61621200	-0.16599700	2.99486200
H	-2.40069000	-0.94796900	3.51081300

H	-0.49669700	-0.24961600	3.48268500
H	-2.01699000	0.98849500	3.05020600
C	-3.00668800	-0.78765900	0.26397600
C	-3.19687700	-1.70834000	-0.78024800
C	-4.07954800	0.04804000	0.61216000
C	-4.41649100	-1.79388300	-1.44789300
H	-2.38356900	-2.37117200	-1.06848100
C	-5.29772200	-0.03269800	-0.06121600
H	-3.96087900	0.75685200	1.42782600
C	-5.47316400	-0.95310500	-1.09438400
H	-4.54301600	-2.52125500	-2.24589300
H	-6.11607600	0.62120800	0.22951900
H	-6.42450000	-1.01955000	-1.61490100
C	2.35063600	-0.76405300	-0.38461300
C	2.99322000	-0.96112500	-1.60709900
C	2.33036300	-1.77049300	0.58154900
C	3.62661400	-2.17510900	-1.86233200
H	2.99563400	-0.16525200	-2.34617800
C	2.96818600	-2.98081000	0.31756600
H	1.80982000	-1.61499400	1.52036400
C	3.61705000	-3.18369200	-0.89974600
H	4.12806300	-2.33022900	-2.81285200
H	2.95061100	-3.76820900	1.06495700
H	4.11174900	-4.12954500	-1.09961600
H	0.05100400	0.09986000	-1.34439600

TS4 (toluene); E_H = −1199.38447630

C	0.16860200	2.16500200	-0.55451800
C	0.52333700	0.70947200	-0.67117600
C	2.24114900	1.57263200	0.69798000
C	1.08499400	2.59465700	0.61097300
H	1.46310700	3.61280600	0.48032800
H	0.51000800	2.56611800	1.54158600
C	-1.30344100	2.46412900	-0.29376600
H	-1.46189500	3.54789100	-0.30982200
H	-1.94264600	2.01694100	-1.06141900
H	-1.61895500	2.08153500	0.67956300
C	0.58022200	2.81494600	-1.89391900
H	-0.05350900	2.45635600	-2.71162200
H	0.45502100	3.90021200	-1.82096500
H	1.62252100	2.60929100	-2.15373700
C	2.45494700	1.09353200	2.13114400
H	3.28959900	0.38922200	2.20203600
H	2.69192100	1.95761100	2.75981400
H	1.55019100	0.62135800	2.52584300
C	3.55552200	2.08382900	0.11055600
H	3.93501500	2.89768900	0.73565100
H	4.31156500	1.29254200	0.09482500
H	3.42996300	2.46541200	-0.90634300
N	1.71340900	0.46560500	-0.16312100
P	-1.37464200	-0.68368200	1.01727900
H	-1.00654700	-2.04972600	0.89681300
B	-1.47950400	-0.20350800	2.91852400
H	-2.30034900	-0.91305000	3.47377700

H	-0.34928500	-0.36997500	3.35453100
H	-1.78141700	0.98081800	2.95914000
C	-3.02613900	-0.77345600	0.24587200
C	-3.26981700	-1.58284800	-0.87573300
C	-4.07249800	0.02763900	0.72684000
C	-4.51773000	-1.59402300	-1.49324000
H	-2.47580300	-2.21800900	-1.26339200
C	-5.31898700	0.02253600	0.10271600
H	-3.90938900	0.64337600	1.60756900
C	-5.54826000	-0.78666000	-1.00918400
H	-4.68760600	-2.23672300	-2.35346600
H	-6.11828100	0.64587300	0.49554900
H	-6.52231700	-0.79474600	-1.49047300
C	2.39441200	-0.77448900	-0.36173000
C	3.09943400	-0.98096400	-1.54758500
C	2.33285400	-1.77070300	0.61359000
C	3.75645700	-2.19130600	-1.75576100
H	3.12905800	-0.19394100	-2.29587100
C	2.99488200	-2.97723000	0.39739600
H	1.76076900	-1.60808600	1.52106600
C	3.70733700	-3.18784800	-0.78222200
H	4.30698900	-2.35348600	-2.67760400
H	2.94561100	-3.75630100	1.15210500
H	4.22046900	-4.13105500	-0.94484000
H	0.11288300	0.03532200	-1.41351500

TS4’ (THF); E_H = −1199.39414559

C	0.37141700	2.05115200	-1.41569000
C	0.86782800	0.64306500	-1.20941200
C	1.09063300	1.62733200	0.93154600
C	0.18611600	2.51143300	0.04415300
H	0.41801300	3.57243700	0.17329000
H	-0.85909900	2.36161300	0.33365900
C	-0.89672100	2.19651400	-2.24694800
H	-1.11946300	3.25950500	-2.38860700
H	-0.78051600	1.73792300	-3.23330700
H	-1.75113800	1.72693800	-1.75506700
C	1.51298600	2.80901100	-2.12830300
H	1.64747300	2.43472400	-3.14826100
H	1.25675300	3.87180400	-2.18768900
H	2.46797700	2.71893800	-1.60399900
C	0.33654300	1.12907900	2.16236600
H	0.97573900	0.52657400	2.81442900
H	-0.00075100	1.99613300	2.73940900
H	-0.54395200	0.54490300	1.87887600
C	2.38352200	2.31911000	1.36417200
H	2.14084000	3.14501700	2.03938400
H	3.03665400	1.62489200	1.90229100
H	2.93660300	2.72457700	0.51326500
N	1.42226100	0.51319300	-0.01138200
P	-1.26378100	-1.04664400	-1.40219800
C	2.29414900	-0.56827900	0.31338000
C	3.55295900	-0.62790700	-0.28895600
C	1.89261800	-1.56672600	1.20283200

C	4.41121900	-1.68521400	0.00204300
H	3.85585500	0.15671000	-0.97643000
C	2.75986600	-2.61758000	1.49477700
H	0.90463000	-1.53213400	1.64775800
C	4.01778800	-2.67911400	0.89730600
H	5.38926600	-1.72795300	-0.46781700
H	2.44436800	-3.39568500	2.18343200
H	4.68850700	-3.50208900	1.12558600
H	-0.60490600	-2.23717000	-1.00158800
H	-2.83575700	-2.38864300	-3.02391900
H	-2.82835400	-0.38906400	-3.36463600
H	-1.25461700	-1.50451800	-3.93793900
B	-2.15661000	-1.37873200	-3.12221200
C	-2.46617600	-0.93163400	-0.03642700
C	-2.30745600	-1.65007400	1.15983700
C	-3.57056000	-0.07066900	-0.14916400
C	-3.21451700	-1.50704100	2.20844700
H	-1.47518900	-2.34185000	1.26686200
C	-4.47564700	0.07342000	0.89984300
H	-3.73260700	0.47765700	-1.07393600
C	-4.30074600	-0.64090700	2.08578900
H	-3.07490800	-2.08026600	3.12139700
H	-5.32576000	0.74125900	0.78728500
H	-5.00903500	-0.53060500	2.90204500
H	1.23320100	0.02192600	-2.01995000

TS4' (toluene); $E_H = -1199.38594867$

C	0.35589600	2.02885400	-1.41157400
C	0.85227000	0.62021700	-1.20192400
C	1.09495800	1.61850000	0.93308500
C	0.17993800	2.49473700	0.04801500
H	0.40734200	3.55781000	0.17123600
H	-0.86247200	2.34058000	0.34519500
C	-0.91920900	2.16662100	-2.23331700
H	-1.13621200	3.22744400	-2.39983400
H	-0.82246800	1.67876900	-3.20745100
H	-1.77194200	1.71637100	-1.72099400
C	1.49079100	2.78678600	-2.13362700
H	1.61374900	2.41471700	-3.15601200
H	1.23890400	3.85102400	-2.18999900
H	2.45106200	2.69229600	-1.61887100
C	0.34959800	1.12191200	2.17058200
H	0.99517400	0.52264100	2.81972700
H	0.01227900	1.98819100	2.74912500
H	-0.53102500	0.53448800	1.89431500
C	2.38449600	2.32243500	1.35770200
H	2.14168100	3.14674800	2.03519100
H	3.04841500	1.63387100	1.89009700
H	2.92750100	2.73242500	0.50220400
N	1.42877500	0.50582800	-0.00613700
P	-1.27992100	-1.06830500	-1.39862700
C	2.30292400	-0.57171700	0.31789900
C	3.56296000	-0.62796700	-0.28241000
C	1.90416900	-1.57517600	1.20316200

C	4.42428300	-1.68299400	0.00639300
H	3.86260100	0.15908200	-0.96879700
C	2.77421700	-2.62376000	1.49416000
H	0.91386800	-1.54459300	1.64351800
C	4.03299700	-2.67997900	0.89876100
H	5.40268000	-1.72247600	-0.46315100
H	2.46003300	-3.40564900	2.17922700
H	4.70555800	-3.50195700	1.12519600
H	-0.62897600	-2.26387800	-1.00219600
H	-2.80276400	-2.32238100	-3.11151900
H	-2.78160400	-0.30601900	-3.35957000
H	-1.19167200	-1.39822900	-3.93651700
B	-2.12446800	-1.31170000	-3.15276400
C	-2.47652200	-0.93523800	-0.03322900
C	-2.30526600	-1.62397100	1.17847800
C	-3.59193700	-0.09181700	-0.16366200
C	-3.20943300	-1.46669800	2.22678300
H	-1.46723000	-2.30752000	1.29475400
C	-4.49335000	0.06729400	0.88581300
H	-3.76668600	0.42286400	-1.10520700
C	-4.30489300	-0.61503800	2.08798500
H	-3.06246800	-2.01894700	3.15150300
H	-5.35404900	0.71890400	0.75957400
H	-5.01208600	-0.49442600	2.90374400
H	1.22505300	0.00537000	-2.01441200

C-3$_{pair}$ (THF); $E_H = -1199.38960487$

C	-3.06548000	-1.59079100	0.17899400
C	-1.82375300	-0.91275600	0.64620900
C	-3.29523900	0.88862600	0.33296600
C	-4.08758500	-0.42810400	0.18456800
H	-4.69237300	-0.41808500	-0.72537700
H	-4.76946300	-0.53963800	1.03305200
C	-3.43551500	-2.71077700	1.16201200
H	-4.37905300	-3.16544300	0.84463300
H	-2.66509600	-3.48714700	1.17511600
H	-3.56617500	-2.32857700	2.17912000
C	-2.83746000	-2.18932600	-1.21980100
H	-2.03736900	-2.93396500	-1.19824000
H	-3.76226800	-2.67749600	-1.54344600
H	-2.57294800	-1.42303500	-1.95300800
C	-3.86081500	1.79741400	1.41587500
H	-3.26572100	2.70766100	1.53157600
H	-4.87454700	2.09087700	1.12821400
H	-3.91642800	1.28357100	2.38007200
C	-3.13717600	1.64492400	-0.98353500
H	-4.12475300	1.97781500	-1.31511400
H	-2.50847000	2.53139500	-0.86197500
H	-2.70842100	1.01152100	-1.76515000
N	-1.94417000	0.36372800	0.76175800
C	-0.88346000	1.22010000	1.22342200
C	-0.03204600	1.82019800	0.29898400
C	-0.73355500	1.42448400	2.59314400
C	0.98707400	2.64835900	0.76373500

H	-0.14731300	1.62112100	-0.76087200
C	0.29000500	2.25418300	3.04387800
H	-1.40445600	0.93612600	3.29310800
C	1.14619700	2.86832500	2.13091200
H	1.66362400	3.11194900	0.05259600
H	0.41848500	2.41557200	4.10964000
H	1.94509700	3.51222300	2.48615800
H	1.98244900	-3.58635600	0.61142900
H	0.89974100	-2.12724600	1.48784100
H	-0.03649900	-3.55167400	0.43838200
B	0.98946600	-2.88124000	0.51890300
C	2.67765100	-0.99150800	-1.22250700
C	3.26770200	-0.70402900	-2.46533100
C	3.37190800	-0.60597100	-0.06491700
C	4.50326000	-0.06564300	-2.54782100
H	2.75325400	-0.98786700	-3.38141100
C	4.60402600	0.04316800	-0.14574700
H	2.94520400	-0.83089300	0.90922800
C	5.17859300	0.31621700	-1.38676800
H	4.94110700	0.13446100	-3.52290200
H	5.12180000	0.32702200	0.76747800
H	6.14183500	0.81511500	-1.45021100
P	1.00996500	-1.74995200	-1.10245700
H	1.15869600	-2.67977200	-2.16951300
H	-0.89031100	-1.41531700	0.90768500

C-3$_{pair}$ (toluene); E_H = −1199.37491371

C	-3.23224900	-1.54318100	-0.12793100
C	-1.94754400	-0.81890900	-0.35864800
C	-3.15180800	0.68319900	0.98936900
C	-3.89422300	-0.66924100	0.96185100
H	-4.96237800	-0.53044900	0.77492500
H	-3.78875500	-1.16278500	1.93273200
C	-3.05153900	-2.99534900	0.31560300
H	-4.03905300	-3.42400500	0.51679200
H	-2.56112500	-3.58922900	-0.45855800
H	-2.44049200	-3.06434300	1.21800400
C	-3.99912400	-1.50800700	-1.46725600
H	-3.45223900	-2.04603800	-2.24717300
H	-4.96649500	-2.00079100	-1.32835800
H	-4.18199900	-0.48697000	-1.81471700
C	-2.70916300	1.07976800	2.39275000
H	-2.14268400	2.01533300	2.38694400
H	-3.59844700	1.22465600	3.01325900
H	-2.09641400	0.29770800	2.85040600
C	-3.93958700	1.81514800	0.33495200
H	-4.84204000	1.99618400	0.92591800
H	-3.36370500	2.74425200	0.31263600
H	-4.24747700	1.56336100	-0.68413600
N	-1.93220000	0.36112900	0.15982100
C	-0.84779900	1.29433100	-0.01247400
C	-0.95134800	2.29060000	-0.98092900
C	0.28560200	1.16794700	0.78385900
C	0.10221500	3.18648900	-1.14055300

H	-1.82976900	2.35060100	-1.61514300
C	1.33519400	2.06607300	0.60878700
H	0.35920900	0.35450200	1.49851800
C	1.24142100	3.07697900	-0.34471700
H	0.03669800	3.95990400	-1.89968900
H	2.23775700	1.95065200	1.20047700
H	2.06689600	3.76885500	-0.48213800
H	1.37396600	-3.48273600	0.88262700
H	-0.06131200	-2.06884600	0.93199300
H	-0.18315400	-3.55695000	-0.40432700
B	0.59198900	-2.83242500	0.20679000
C	2.96986400	-0.98366000	-0.46470300
C	3.88066900	-0.28851600	-1.27662400
C	3.21615300	-1.01434400	0.91494200
C	4.99914800	0.34026600	-0.73479500
H	3.70481200	-0.23668700	-2.34936200
C	4.32709200	-0.37156400	1.46430500
H	2.53626000	-1.56810300	1.55728800
C	5.22525800	0.30800900	0.64303800
H	5.69433500	0.86252600	-1.38804900
H	4.49848000	-0.41616600	2.53763500
H	6.09521300	0.80167400	1.06835700
P	1.45476200	-1.74350200	-1.19286300
H	2.11039900	-2.71972400	-1.99434900
H	-1.10644700	-1.15538200	-0.97028200

TS5 (THF); E_H = −1199.38381031

C	-2.98581100	-1.45739900	-0.12614600
C	-1.86528700	-0.66074300	-0.73666300
C	-2.39260000	0.55930600	1.20997300
C	-3.05774300	-0.83247900	1.28254700
H	-4.08705200	-0.76253900	1.64458800
H	-2.50142400	-1.46484400	1.98158000
C	-2.78967200	-2.96888700	-0.09356900
H	-3.66833900	-3.43070800	0.36841800
H	-2.68049700	-3.37390700	-1.10336200
H	-1.90028300	-3.24308700	0.47695600
C	-4.23483600	-1.13679900	-0.97702400
H	-4.12598300	-1.53172100	-1.99189300
H	-5.10489400	-1.61614800	-0.51758300
H	-4.43382300	-0.06414200	-1.04644100
C	-1.39078100	0.76647400	2.34141400
H	-0.91520200	1.74987600	2.28765900
H	-1.92366000	0.70342300	3.29511700
H	-0.61713500	-0.00684200	2.33327900
C	-3.39162600	1.71383300	1.17423900
H	-3.91589200	1.75852000	2.13311600
H	-2.88260400	2.67051800	1.02359600
H	-4.13659400	1.58745100	0.38453200
N	-1.68686900	0.47513700	-0.11478700
C	-0.91982400	1.55718000	-0.65976400
C	-1.49307900	2.35306200	-1.65165000
C	0.37598500	1.79883400	-0.20721600
C	-0.76027600	3.40659800	-2.19241700

H	-2.50320500	2.14652900	-1.99289800
C	1.09947900	2.85673300	-0.75291200
H	0.82594000	1.15451300	0.54030400
C	0.53391300	3.66131800	-1.74059500
H	-1.20238300	4.02656500	-2.96638700
H	2.11378500	3.04036600	-0.41206900
H	1.10469300	4.48223400	-2.16432800
H	0.75357400	-3.25328900	0.11091100
H	-0.40617800	-1.68578400	-0.31330500
H	-0.08815000	-3.05043200	-1.73193000
B	0.44370800	-2.48893000	-0.78865400
C	3.01902400	-0.97712100	-0.11136400
C	4.33582600	-0.54494900	-0.34396900
C	2.55984700	-0.98366600	1.21373100
C	5.16367600	-0.15214900	0.70496200
H	4.71712100	-0.51633200	-1.36296200
C	3.38055500	-0.57250100	2.26604200
H	1.55388200	-1.33911300	1.42321500
C	4.68760300	-0.15739000	2.01815000
H	6.18230800	0.16629900	0.49702000
H	2.99890300	-0.58961700	3.28428700
H	5.33020100	0.15589700	2.83646700
P	1.91243200	-1.40615400	-1.52268400
H	2.73992200	-2.42174000	-2.08318300
H	-1.51568600	-0.77087900	-1.75778400

TS5 (toluene); E_H = −1199.37440703

C	-3.10682300	-1.47115900	-0.17364000
C	-1.87165600	-0.72492700	-0.58083600
C	-2.77319000	0.63457900	1.11492900
C	-3.49651400	-0.72886600	1.12309000
H	-4.57947800	-0.60434300	1.20887800
H	-3.15969800	-1.31450200	1.98397500
C	-2.93871100	-2.97255400	0.03948400
H	-3.89977500	-3.39052000	0.35784700
H	-2.63041700	-3.47250000	-0.88156800
H	-2.18357800	-3.18472700	0.79830500
C	-4.12290100	-1.23621800	-1.31441700
H	-3.77969200	-1.69863600	-2.24497800
H	-5.07506100	-1.69937100	-1.03713000
H	-4.30219500	-0.17399800	-1.50418400
C	-2.02704200	0.89543900	2.41908400
H	-1.48981200	1.84780200	2.39409600
H	-2.75299200	0.93799500	3.23670400
H	-1.31700200	0.09172100	2.63481300
C	-3.68924600	1.80919000	0.78073100
H	-4.41733600	1.93052900	1.58797900
H	-3.12365800	2.74152600	0.69521600
H	-4.23903900	1.64631700	-0.15035000
N	-1.78669900	0.43402500	-0.00308700
C	-0.82667000	1.43760400	-0.37169100
C	-1.15456400	2.35533500	-1.36767600
C	0.41092300	1.46962700	0.26294400
C	-0.22409500	3.32719300	-1.72604400

H	-2.12114300	2.29974600	-1.85963500
C	1.33631800	2.44182000	-0.10723900
H	0.66238100	0.72375900	1.00893200
C	1.01829800	3.37186000	-1.09470300
H	-0.46805000	4.04257000	-2.50551800
H	2.31388100	2.45117700	0.36486000
H	1.74551000	4.12470400	-1.38389200
H	1.00316200	-3.28479600	0.59609900
H	-0.33171800	-1.82176500	0.31062800
H	-0.18321400	-3.27943700	-1.04922200
B	0.46138500	-2.60514600	-0.26020300
C	3.03551700	-0.91058700	-0.30822200
C	4.16465100	-0.30174400	-0.88075200
C	2.95745700	-0.95266300	1.09094500
C	5.18002400	0.22972600	-0.08950200
H	4.24441300	-0.24160800	-1.96446600
C	3.96346400	-0.40328700	1.88820000
H	2.10790200	-1.44679200	1.55614200
C	5.08115000	0.18891300	1.30299500
H	6.04880000	0.68418300	-0.55996500
H	3.87951900	-0.45368400	2.97154400
H	5.86936900	0.60891800	1.92225400
P	1.66746600	-1.52770400	-1.38209200
H	2.41391900	-2.54108900	-2.04708500
H	-1.23855100	-0.95326100	-1.43401600

G (THF); E_H = −600.21989081

C	2.38354500	-0.70239900	0.06430300
C	2.27801300	0.72523200	-0.48911000
H	3.02925500	1.39881200	-0.06337100
H	2.44515700	0.69583900	-1.57280500
C	0.84085500	1.23072500	-0.21250600
C	2.72424200	-0.73061200	1.55813500
H	2.83244400	-1.76513000	1.90382700
H	1.94311000	-0.26045300	2.16206900
H	3.66865100	-0.21007800	1.75287600
C	3.40502600	-1.53752400	-0.70373200
H	4.41623100	-1.14041400	-0.55994700
H	3.19158100	-1.53890300	-1.77846200
H	3.40472600	-2.57693600	-0.35545400
C	0.84411800	2.19621800	0.98229600
H	1.46538100	3.06560700	0.74114200
H	1.25922800	1.72058400	1.87398700
H	-0.15150400	2.56726300	1.23413200
C	0.30834100	1.94049300	-1.46395800
H	-0.68578400	2.37060800	-1.31981100
H	0.25997100	1.24324600	-2.30692300
H	0.98799000	2.75563700	-1.73488000
C	-1.26266300	-0.18704700	0.05400800
C	-3.53374600	0.68270300	0.30398300
H	-4.18066000	1.53489700	0.49772100
C	-4.08837200	-0.57750900	0.07803900
H	-5.16405200	-0.72437500	0.08933400
C	-3.22127200	-1.64048800	-0.16287700

H	-3.61743200	-2.63582700	-0.34910300
N	0.11212900	-0.03518300	0.07474200
C	-2.15785100	0.87992000	0.29664000
H	-1.78289300	1.87458800	0.49523900
C	-1.84115700	-1.45651300	-0.18181000
H	-1.21082700	-2.31283000	-0.39036100
C	0.95481800	-1.19582800	-0.16504600
H	0.83992200	-1.57769300	-1.19564000
H	0.69373200	-2.01145800	0.51997300

G (toluene); E_H = −600.21792944

C	2.38387800	-0.70258100	0.06552500
C	2.27784000	0.72404700	-0.49059100
H	3.02991500	1.39836100	-0.06706200
H	2.44416700	0.69289200	-1.57448700
C	0.84062300	1.22940500	-0.21304800
C	2.72410700	-0.72751200	1.55946800
H	2.83578600	-1.76095000	1.90727500
H	1.94021100	-0.25983400	2.16153100
H	3.66683000	-0.20404300	1.75488800
C	3.40511800	-1.53923100	-0.70103300
H	4.41683000	-1.14290600	-0.55849100
H	3.19209000	-1.54310400	-1.77587200
H	3.40510800	-2.57817200	-0.35130000
C	0.84708100	2.19916500	0.97854100
H	1.46905700	3.06742800	0.73453700
H	1.26135900	1.72548500	1.87158500
H	-0.14779600	2.57172400	1.23051900
C	0.30415500	1.93518300	-1.46510800
H	-0.69241000	2.35914300	-1.32022900
H	0.25596000	1.23625900	-2.30663000
H	0.97907800	2.75333600	-1.73927100
C	-1.26295700	-0.18687600	0.05711800
C	-3.53227600	0.68176000	0.31181700
H	-4.17929700	1.53216400	0.51254000
C	-4.08659400	-0.57574000	0.07450500
H	-5.16225900	-0.72216400	0.08333700
C	-3.22048000	-1.63684000	-0.17391400
H	-3.61717500	-2.63024100	-0.36862500
N	0.11373600	-0.03491200	0.08021300
C	-2.15661700	0.87790200	0.30814900
H	-1.78040300	1.86988400	0.51752900
C	-1.84062800	-1.45323200	-0.18962400
H	-1.21019900	-2.30785800	-0.40453300
C	0.95448300	-1.19494100	-0.16220900
H	0.83887500	-1.57709000	-1.19314700
H	0.69305900	-2.01073900	0.52274600

H (THF); E_H = −599.18608125

B	-3.01669900	0.68511100	0.65148500
H	-4.13489600	0.31547000	0.85675300
H	-2.66212000	1.79014200	0.93738500
P	-1.93934400	-0.37924800	-0.39003100
H	-2.16345300	-1.73771600	-0.09828200
C	-0.15302400	-0.14761100	-0.14545700
C	0.40552100	1.13375600	-0.24440600
C	0.68853400	-1.24256700	0.08742000
C	1.77739100	1.31624400	-0.08960300
H	-0.23127000	1.99270100	-0.44030900
C	2.06230600	-1.05535800	0.23226500
H	0.27377300	-2.24436000	0.16350000
C	2.61119300	0.22315400	0.14770400
H	2.19621700	2.31613200	-0.16134400
H	2.70282900	-1.91345200	0.41614800
H	3.68152700	0.36722100	0.26266100

H (toluene); E_H = −599.18466517

B	-3.01476400	0.68120900	0.65602500
H	-4.13158400	0.30917000	0.86402200
H	-2.66077100	1.78521200	0.94641900
P	-1.93943900	-0.37422500	-0.39731100
H	-2.16203900	-1.73413100	-0.10862500
C	-0.15348000	-0.14575200	-0.14867400
C	0.40677000	1.13431500	-0.24930800
C	0.68664200	-1.24033300	0.08858600
C	1.77804500	1.31580600	-0.09200100
H	-0.22922600	1.99302200	-0.44870900
C	2.05994900	-1.05450800	0.23564100
H	0.27047600	-2.24147200	0.16657000
C	2.61016100	0.22285300	0.14937000
H	2.19803300	2.31512100	-0.16511200
H	2.69931400	-1.91277100	0.42284100
H	3.68039800	0.36610400	0.26615800

TS2 (THF); E_H = −1199.32957625

P	-2.91289300	-0.76091200	-1.65460400
H	-3.54138400	-1.79524400	-2.39142000
C	-4.29765800	-0.29213600	-0.55703600
C	-5.36984200	-1.15976000	-0.31434200
C	-4.23889500	0.92522700	0.13586800
C	-6.36907300	-0.81135000	0.59352000
H	-5.43138100	-2.10988100	-0.83871800
C	-5.24333900	1.27456700	1.03546000
H	-3.40548400	1.60451300	-0.02589800
C	-6.31106000	0.40732100	1.26760800
H	-7.19668300	-1.49313300	0.76891900
H	-5.18896200	2.22495500	1.55913700
H	-7.09211400	0.67947800	1.97176500
B	-0.79560400	-0.94801500	-0.66487500
H	-0.90261000	0.16770000	-0.21893200
H	-0.23843400	-1.12021400	-1.72392600
H	-3.01944900	0.24591400	-2.64658500
H	-1.10593500	-1.89868800	0.00828500
C	1.55090700	-1.06537800	0.17873500

C	2.36518400	-2.34139400	0.28763000
C	3.82905100	-1.93901100	-0.01987000
H	4.54399000	-2.40450600	0.66613600
H	4.09626600	-2.25650800	-1.03398000
C	3.88684700	-0.40700900	0.07060900
C	2.19536000	-2.86066500	1.72481300
H	1.14246900	-3.07086800	1.93708000
H	2.55043500	-2.13732200	2.46605100
H	2.76701700	-3.78708900	1.85404300
C	1.84823500	-3.39943800	-0.68938600
H	2.44514800	-4.31532200	-0.60270500
H	1.91180700	-3.04744800	-1.72439600
H	0.80251200	-3.64094600	-0.47957500
C	4.50397600	0.09630700	1.37553600
H	5.56596300	-0.16782700	1.40292800
H	4.01788300	-0.35134000	2.24725900
H	4.42671300	1.18524300	1.45686300
C	4.60570400	0.21814800	-1.12046900
H	4.61035100	1.31116000	-1.06685800
H	4.14541900	-0.08732200	-2.06530300
H	5.64695300	-0.12054900	-1.12612800
C	1.97479100	1.28679200	0.02503500
C	1.20612800	3.25397800	1.19091600
H	0.93865800	3.74933500	2.11992800
C	1.11322000	3.93819500	-0.02069800
H	0.77542800	4.97038900	-0.03929000
C	1.44721000	3.29008800	-1.20927200
H	1.36528600	3.81322000	-2.15776400
N	2.40688700	-0.08205300	0.05743900
C	1.63723500	1.92925500	1.21543400
H	1.69970900	1.38220600	2.15135500
C	1.87946600	1.96521700	-1.18920800
H	2.12273600	1.45109800	-2.11353000

TS2 (toluene); E_H = −1199.32446882

P	-2.90425000	-0.80812600	-1.60385900
H	-3.52823500	-1.85335800	-2.33129800
C	-4.29979400	-0.33974700	-0.51518200
C	-5.55395300	-0.95803300	-0.59003400
C	-4.08065400	0.63504800	0.46733400
C	-6.57289600	-0.59827600	0.29012000
H	-5.73936800	-1.72168000	-1.34096000
C	-5.10449200	1.00154500	1.33816200
H	-3.10307100	1.10174500	0.55674500
C	-6.35230500	0.38570800	1.25249200
H	-7.54114100	-1.08648100	0.22084000
H	-4.92262600	1.76272300	2.09179600
H	-7.14791200	0.66735600	1.93652900
B	-0.79005900	-0.95844700	-0.62997900
H	-0.87731300	0.17600800	-0.22939600
H	-0.24422700	-1.17337500	-1.68769000
H	-3.02124700	0.17957200	-2.61636800
H	-1.09230000	-1.87815300	0.08718900
C	1.56283000	-1.05017800	0.19515700

C	2.38119200	-2.32081800	0.32472700
C	3.84368600	-1.92183400	0.00323200
H	4.56198500	-2.36858300	0.69854600
H	4.10965500	-2.26369700	-1.00340500
C	3.89673100	-0.38756300	0.05669000
C	2.21653300	-2.81085500	1.77247000
H	1.16278100	-3.00629300	1.99285000
H	2.58049300	-2.07518300	2.49733400
H	2.78179500	-3.73901800	1.91781100
C	1.85917100	-3.39754000	-0.62885100
H	2.45504200	-4.31280300	-0.52744300
H	1.91626700	-3.06546000	-1.67073500
H	0.81381100	-3.63125400	-0.40982400
C	4.52361600	0.14853800	1.34394500
H	5.58786700	-0.10739900	1.36872600
H	4.04802800	-0.28328500	2.22944300
H	4.43884200	1.23844300	1.40225300
C	4.60383900	0.21191400	-1.15475300
H	4.60186800	1.30596800	-1.12834800
H	4.13708300	-0.11892900	-2.08789600
H	5.64717300	-0.12077900	-1.16203700
C	1.97799200	1.29808100	-0.00910700
C	1.20630200	3.28358800	1.12225900
H	0.93983500	3.79524300	2.04276000
C	1.10506700	3.94415500	-0.10135300
H	0.76249900	4.97431200	-0.13807700
C	1.43391100	3.27424900	-1.27869600
H	1.34182900	3.77774900	-2.23685500
N	2.41627700	-0.06775900	0.04791600
C	1.64230500	1.96156900	1.16987300
H	1.70654600	1.43001300	2.11443900
C	1.87126900	1.95204600	-1.23531300
H	2.10587000	1.41831400	-2.15059500

I (THF); E_H = −573.78289764

P	2.32671600	-0.06427900	-0.09942100
H	2.56089700	1.31153600	-0.35758700
C	0.48715200	0.01188400	-0.03488200
C	-0.22797100	1.21660700	-0.04194800
C	-0.22604500	-1.19393600	-0.01278000
C	-1.62184700	1.21431800	-0.01797100
H	0.30482200	2.16362700	-0.06400900
C	-1.61975800	-1.19631200	0.02681200
H	0.31041400	-2.13989700	-0.02978700
C	-2.32116500	0.00838200	0.02206700
H	-2.16150200	2.15740400	-0.02638100
H	-2.15667500	-2.14056100	0.05054400
H	-3.40727400	0.00794100	0.04371800
H	2.58760400	0.05317200	1.29475000

I (toluene); E_H = −573.78144763

P	2.32797800	-0.09764700	-0.04140400

H	2.57458200	1.22383400	-0.50089200
C	0.48713300	-0.00323600	-0.01183700
C	-0.22128500	1.20551900	-0.00308300
C	-0.23271000	-1.20418900	-0.01933300
C	-1.61436300	1.21117100	0.00626000
H	0.31784700	2.14929000	-0.00244400
C	-1.62711700	-1.19958800	0.00293500
H	0.30067700	-2.15143100	-0.04766100
C	-2.32067200	0.00840900	0.01413400
H	-2.14918500	2.15705000	0.01009300
H	-2.16976300	-2.14083400	0.00159200
H	-3.40697500	0.01424000	0.02324400
H	2.56921900	0.23729800	1.32152100

J (THF); E_H = −625.62147152

C	0.98789900	-0.95459200	-0.15953200
C	2.40309300	-0.41719500	-0.10215700
C	2.22921100	1.11556000	-0.16455800
H	2.87113100	1.63133000	0.55519100
H	2.49952400	1.47792400	-1.16184300
C	0.74530500	1.41216300	0.10273500
C	3.04270900	-0.88760000	1.21468200
H	3.07724600	-1.97973100	1.25597300
H	2.48971000	-0.52967400	2.08861800
H	4.06621800	-0.50265000	1.27711300
C	3.23892500	-0.92731200	-1.28081600
H	4.22703900	-0.45489700	-1.25225900
H	2.77073900	-0.67690700	-2.23855600
H	3.36747200	-2.01095300	-1.23343100
C	0.48412100	1.89727400	1.52879900
H	0.96535000	2.86998300	1.66828500
H	0.89274700	1.20482800	2.27048800
H	-0.58617400	2.02139800	1.71858400
C	0.16311300	2.39955200	-0.90259900
H	-0.90063000	2.58266200	-0.72482800
H	0.29491000	2.04444500	-1.92915600
H	0.68982600	3.35360300	-0.80329700
C	-1.27924500	-0.13038000	-0.04264600
C	-3.29022600	-0.57486400	1.20318900
H	-3.78014500	-0.80354500	2.14500300
C	-4.04047000	-0.44575400	0.03532600
H	-5.11886700	-0.57093800	0.06539700
C	-3.40410800	-0.16364300	-1.17293000
H	-3.98310900	-0.07137100	-2.08701400
N	0.14478700	0.04584300	-0.07908600
C	-1.90633100	-0.41762800	1.16774300
H	-1.31240400	-0.53271500	2.06877700
C	-2.02105800	-0.00428500	-1.21543900
H	-1.51588100	0.20154600	-2.15375000
H	-0.41719600	-2.66507200	-0.87277000
H	1.52362300	-3.17485100	-0.56705200
H	0.31510000	-2.76418200	0.96934300
B	0.58481700	-2.49028800	-0.19967700

J (toluene); E_H = −625.61588641

C	0.98606600	-0.95713100	-0.15812600
C	2.40166500	-0.41828000	-0.10160600
C	2.22829500	1.11478300	-0.16673300
H	2.86878000	1.63216900	0.55363500
H	2.50130200	1.47614100	-1.16397500
C	0.74356800	1.41118100	0.09665100
C	3.04568400	-0.88677400	1.21325600
H	3.07648300	-1.97864700	1.25532500
H	2.49445400	-0.52886900	2.08849400
H	4.06995500	-0.50271200	1.27325800
C	3.23389100	-0.93151200	-1.28160500
H	4.22430200	-0.46314100	-1.25679600
H	2.76369600	-0.68221500	-2.23874600
H	3.35543400	-2.01569100	-1.23377500
C	0.47969200	1.89207400	1.52413200
H	0.95654600	2.86630100	1.66965000
H	0.88996300	1.19766800	2.26321900
H	-0.59148600	2.00993500	1.71336300
C	0.16678700	2.40576000	-0.90515500
H	-0.89779700	2.58811400	-0.73160600
H	0.30198800	2.05590200	-1.93322500
H	0.69338500	3.35946400	-0.79994000
C	-1.28092000	-0.12758600	-0.05429700
C	-3.28846700	-0.59567000	1.18826600
H	-3.77520800	-0.84682300	2.12600400
C	-4.04236700	-0.44181500	0.02618600
H	-5.12062300	-0.56820200	0.05688200
C	-3.40955800	-0.13771300	-1.17802200
H	-3.99101900	-0.03068100	-2.08896400
N	0.14306500	0.04784000	-0.09091200
C	-1.90507700	-0.43950100	1.15126300
H	-1.30675900	-0.58091100	2.04553500
C	-2.02671000	0.02050300	-1.22158100
H	-1.52347000	0.23797200	-2.15824600
H	-0.46141300	-2.67894800	-0.75568900
H	1.50677800	-3.17173700	-0.58736700
H	0.41900500	-2.73492100	1.02431700
B	0.58886300	-2.48924000	-0.17122300

TS3 (THF); E_H = −1199.32545457

P	-1.33936500	-0.45712100	0.00666400
H	-0.96476800	0.84545500	0.38645400
C	-3.19645300	-0.08669000	-0.00400800
C	-4.13017900	-1.07923000	0.31022800
C	-3.66598200	1.17492000	-0.38428800
C	-5.50011800	-0.82250600	0.24652600
H	-3.78681600	-2.06838300	0.61466200
C	-5.03379400	1.44127700	-0.45124400
H	-2.95559100	1.96526700	-0.62824100
C	-5.95361600	0.44047200	-0.13581600
H	-6.21343400	-1.60352100	0.49760800

H	-5.38312600	2.42794800	-0.74498300
H	-7.01972100	0.64493100	-0.18482700
B	-0.68074300	-1.03796000	-1.67442000
H	-0.43621200	-0.13215100	-2.42368100
H	-1.08940800	-2.08082700	-2.11149300
H	-1.44168900	-1.44111700	1.01137000
C	0.71225300	-0.97827500	-0.12658900
C	1.29105700	-2.26560300	0.48981600
C	2.71557500	-2.27067700	-0.10909000
H	3.44731100	-2.72637100	0.56752300
H	2.71154200	-2.86655200	-1.02988600
C	3.09480500	-0.80294300	-0.44382200
C	1.33458200	-2.08927800	2.01336500
H	0.32479100	-2.05447000	2.43701000
H	1.85186600	-1.16742000	2.29499600
H	1.86537400	-2.92917900	2.47622900
C	0.53948600	-3.54829300	0.14575300
H	1.06575700	-4.41199400	0.56705900
H	0.46738500	-3.69563100	-0.93672000
H	-0.47575600	-3.54961100	0.55681100
C	4.16249900	-0.25468900	0.50307200
H	5.09782400	-0.80655400	0.36445400
H	3.85717000	-0.35424000	1.54903900
H	4.36448300	0.80274500	0.30412200
C	3.59217000	-0.71014400	-1.89196000
H	3.95575500	0.29330600	-2.13299800
H	2.79349000	-0.97179000	-2.59450400
H	4.42263900	-1.40815500	-2.04881900
C	1.69790500	1.28550400	-0.00738500
C	1.07268900	3.12898600	1.45197000
H	0.66070300	3.47347100	2.39680900
C	1.51686500	4.05102800	0.50388400
H	1.44739700	5.11707000	0.70013000
C	2.04916000	3.58877100	-0.69881800
H	2.39023000	4.29574400	-1.45043100
N	1.79742600	-0.10118800	-0.24165700
C	1.17327500	1.76227700	1.20417700
H	0.85779300	1.04536300	1.95711300
C	2.12796400	2.22139800	-0.95874600
H	2.50919800	1.87549600	-1.91347800
H	0.58743000	-1.43146000	-1.31623700

TS3 (toluene); $E_H = -1199.32265473$

P	-1.32865200	-0.46467200	0.00803200
H	-0.94705000	0.83130400	0.40596900
C	-3.18184300	-0.07961300	0.00219200
C	-4.12435000	-1.07072000	0.29110500
C	-3.63920200	1.19424800	-0.34886500
C	-5.49139000	-0.80091100	0.23103000
H	-3.78980000	-2.07002800	0.57152400
C	-5.00406500	1.47388400	-0.41198900
H	-2.92086200	1.98317300	-0.57345700
C	-5.93284900	0.47433200	-0.12199400
H	-6.21203700	-1.58151300	0.46164200

H	-5.34426400	2.47006600	-0.68347100
H	-6.99704000	0.68908200	-0.16844100
B	-0.67356500	-1.03308500	-1.68016900
H	-0.42275000	-0.12385300	-2.42224200
H	-1.08362300	-2.07311500	-2.12103100
H	-1.44324200	-1.46388100	0.99817800
C	0.71548300	-0.99227700	-0.12062000
C	1.29428100	-2.28492300	0.48395700
C	2.72457100	-2.27660100	-0.10239400
H	3.45236700	-2.73918800	0.57409500
H	2.73060200	-2.85968600	-1.03140700
C	3.10026500	-0.80293300	-0.41291400
C	1.32356000	-2.12937300	2.01004000
H	0.30991200	-2.10748400	2.42525500
H	1.83136000	-1.20732200	2.30768900
H	1.85584600	-2.97138200	2.46752300
C	0.55023100	-3.56524700	0.11585300
H	1.07443100	-4.43368900	0.53016000
H	0.48695900	-3.69777600	-0.96898800
H	-0.46888000	-3.57443400	0.51707000
C	4.15434700	-0.25939500	0.55205000
H	5.09941200	-0.79391000	0.41040400
H	3.84290600	-0.38259900	1.59364900
H	4.34074700	0.80488300	0.37524900
C	3.61254700	-0.68499400	-1.85413300
H	3.97030000	0.32541600	-2.07445200
H	2.82344500	-0.94140400	-2.56936400
H	4.45070700	-1.37331500	-2.01347800
C	1.68656900	1.27982000	-0.00820400
C	1.09320800	3.14559300	1.43408600
H	0.71787800	3.50664100	2.38790700
C	1.47435100	4.05065600	0.44374900
H	1.39298200	5.11979500	0.61728900
C	1.95706400	3.56959500	-0.77189400
H	2.24588300	4.26447800	-1.55590700
N	1.79917600	-0.11237000	-0.21176100
C	1.20900900	1.77556600	1.21420200
H	0.93902200	1.06982300	1.99505500
C	2.05127200	2.19799600	-1.00194300
H	2.38976100	1.83314000	-1.96572000
H	0.59408500	-1.43038900	-1.31741000

K (THF); $E_H = -1199.42547708$

B	-0.44116600	-0.99160100	-1.06790700
H	-0.91711200	-2.09431500	-1.27026200
P	-1.39719700	-0.32610300	0.49437400
H	-1.22481300	-1.01821400	1.70959900
H	-1.05553900	0.97888600	0.89592500
C	-3.20564600	-0.24911800	0.30461000
C	-3.99544900	0.41455000	1.25219000
C	-3.81216100	-0.86414300	-0.79483300
C	-5.37836000	0.45845200	1.09995800
H	-3.53294000	0.89982400	2.10805700
C	-5.19815400	-0.81765100	-0.94295700

H	-3.20129200	-1.37363000	-1.53399500	H	-3.18380700	-1.32418100	-1.56504700
C	-5.98077900	-0.15861100	0.00250400	C	-5.97547400	-0.15444700	-0.01475900
H	-5.98576400	0.97582300	1.83687000	H	-5.99272700	0.94229600	1.84208200
H	-5.66379200	-1.29606600	-1.79963000	H	-5.64677500	-1.25247000	-1.83859000
H	-7.06004900	-0.12221200	-0.11508900	H	-7.05448800	-0.12038500	-0.13574300
C	1.16975300	-1.02086500	-0.82508000	C	1.17211300	-1.02697500	-0.81930600
C	1.76365700	-2.33313700	-0.26858800	C	1.76362300	-2.33811000	-0.25820300
C	3.08635500	-0.39311400	0.54366300	C	3.09434400	-0.39480200	0.53390000
C	3.17944500	-1.87283400	0.10439700	C	3.18164900	-1.87973400	0.10969800
H	3.82127000	-1.94542800	-0.78237500	H	3.82376500	-1.96470300	-0.77571100
H	3.63315600	-2.49755300	0.88242000	H	3.63237500	-2.49860700	0.89427200
N	1.69733900	-0.00679200	0.11366900	N	1.69999700	-0.01328500	0.11934800
C	4.16178600	0.42768700	-0.17686300	C	4.15810000	0.41997700	-0.21053900
H	4.14880600	1.47781700	0.13241600	H	4.14535400	1.47389300	0.08531200
H	5.15266500	0.01932300	0.05115700	H	5.15353600	0.01748200	0.00822800
H	4.02005400	0.38725500	-1.26248400	H	4.00152400	0.36700400	-1.29351700
C	3.27957600	-0.25618500	2.05727400	C	3.30823700	-0.24203700	2.04305400
H	4.24596000	-0.68565200	2.34468500	H	4.27769400	-0.66985100	2.32258800
H	3.28406500	0.79003600	2.37538900	H	3.31893400	0.80758700	2.34957100
H	2.49431400	-0.78300900	2.60700500	H	2.52926800	-0.76211600	2.60822100
C	1.79772900	-3.43134100	-1.32853500	C	1.79055400	-3.44243900	-1.31164300
H	2.30678500	-4.32375600	-0.94516700	H	2.29711200	-4.33489300	-0.92503600
H	0.78394700	-3.72347700	-1.62332900	H	0.77502600	-3.73148100	-1.60283200
H	2.33033700	-3.09893300	-2.22737900	H	2.32176500	-3.11713500	-2.21381700
C	1.01933600	-2.85790900	0.96267600	C	1.02026900	-2.85099900	0.97863600
H	0.94529000	-2.09873300	1.74676400	H	0.95286700	-2.08470100	1.75665900
H	0.00689200	-3.18250200	0.69919300	H	0.00579000	-3.17274600	0.71958900
H	1.54777000	-3.72173100	1.38349700	H	1.54496600	-3.71448500	1.40479500
H	-0.82936500	-0.21627900	-1.92343300	H	-0.82566500	-0.23066400	-1.92718900
C	1.46103500	1.35329100	-0.16941600	C	1.45748200	1.34743900	-0.15932700
C	1.14844700	1.81177700	-1.46015100	C	1.14023600	1.80834000	-1.44740500
C	1.50610300	2.30810800	0.86488700	C	1.49791500	2.29767300	0.87891800
C	0.90392200	3.16272700	-1.70474300	C	0.88639500	3.15829800	-1.68565300
H	1.09371700	1.10647800	-2.28127300	H	1.08710400	1.10532800	-2.27054900
C	1.28040800	3.65776200	0.61324800	C	1.26357200	3.64671600	0.63313700
H	1.69476800	1.98024100	1.88135000	H	1.68958200	1.96541900	1.89346800
C	0.97494500	4.09893100	-0.67544900	C	0.95316600	4.09041500	-0.65297900
H	0.66318700	3.48298200	-2.71554300	H	0.64117300	3.48099200	-2.69448000
H	1.32363800	4.36682200	1.43623900	H	1.30418400	4.35315300	1.45851400
H	0.78620400	5.15083900	-0.86971500	H	0.75765400	5.14181400	-0.84290900
H	1.64586600	-0.89553300	-1.82104700	H	1.64896700	-0.90405500	-1.81518000

K (toluene); E_H = −1199.42247017

B	-0.43782800	-0.99766200	-1.06521600
H	-0.91925000	-2.10041700	-1.25112600
P	-1.39283400	-0.31133800	0.48739300
H	-1.22650500	-0.98738800	1.71355800
H	-1.05416600	0.99865800	0.87687500
C	-3.20160200	-0.23923700	0.29576100
C	-3.99802100	0.40137800	1.25321700
C	-3.80105000	-0.83385400	-0.81826800
C	-5.38019600	0.44257500	1.09721300
H	-3.54048200	0.87203300	2.12007500
C	-5.18647300	-0.79008900	-0.97028700

L (THF); E_H = −1797.73253917

H	1.82976100	-0.58294700	1.02075700
H	2.86996200	-0.93159200	-0.68551600
H	-0.40730000	1.87969000	0.99785200
H	-0.62690900	2.93954800	-0.71797100
H	-1.42946300	-1.28175400	1.01276800
H	-2.22984800	-2.02515800	-0.69670100
P	0.38817300	-1.79623600	-0.72142700
H	0.43388000	-1.99459000	-2.11848200
P	1.36241400	1.22136000	-0.74001700
H	1.50806700	1.34072500	-2.13918000
P	-1.73534200	0.55617200	-0.75195300

H	-1.90233600	0.60432500	-2.15303000
B	-1.41665300	-1.28395500	-0.19885900
B	1.82242600	-0.59016300	-0.19090500
B	-0.39837700	1.86622400	-0.21370800
C	0.74429000	-3.47810200	-0.12142600
C	0.93120900	-4.53923000	-1.01370300
C	0.82737900	-3.71384300	1.25627300
C	1.19836700	-5.82055800	-0.53317500
H	0.86898900	-4.36975900	-2.08542500
C	1.09407400	-4.99511000	1.73219500
H	0.68464400	-2.89722400	1.95910300
C	1.28001200	-6.05052300	0.83892200
H	1.34244800	-6.63817900	-1.23374200
H	1.15692500	-5.16905600	2.80259600
H	1.48819600	-7.04910900	1.21227400
C	2.64175500	2.37934900	-0.15861000
C	3.46603800	3.05866800	-1.06201700
C	2.80584300	2.58968300	1.21594000
C	4.44211900	3.93829200	-0.59548300
H	3.34951100	2.90385200	-2.13143700
C	3.78209500	3.46878600	1.67784400
H	2.17102100	2.06805200	1.92736400
C	4.60165800	4.14471300	0.77350700
H	5.07724600	4.46161900	-1.30455600
H	3.90222700	3.62620900	2.74590900
H	5.36226600	4.83034400	1.13584000
C	-3.38208500	1.09219800	-0.18934200
C	-4.37700000	1.45007400	-1.10546300
C	-3.65592100	1.15036100	1.18257200
C	-5.63061000	1.86099200	-0.65418400
H	-4.17733400	1.40938100	-2.17302500
C	-4.90925300	1.56140300	1.62924700
H	-2.89128400	0.87409100	1.90381800
C	-5.89864000	1.91729100	0.71226600
H	-6.39689200	2.13662100	-1.37313700
H	-5.11319400	1.60322300	2.69537300
H	-6.87568000	2.23731800	1.06282800

L (toluene); E_H = −1797.72709301

H	1.83491100	-0.58351600	1.02244800
H	2.87193000	-0.92990000	-0.68619400
H	-0.41004100	1.88414700	0.99861400
H	-0.62760500	2.93939800	-0.72034800
H	-1.43277200	-1.28789900	1.01095700
H	-2.22766500	-2.02657600	-0.70312400
P	0.39023400	-1.79486700	-0.71668500
H	0.43761000	-1.99470200	-2.11475300
P	1.36178400	1.22146200	-0.73315200
H	1.50943800	1.34180800	-2.13331800
P	-1.73398200	0.55505600	-0.74712000
H	-1.90291600	0.60500600	-2.14919300
B	-1.41738700	-1.28699900	-0.20025900
B	1.82619500	-0.59006900	-0.18883900
B	-0.39946800	1.86866600	-0.21253800
C	0.74640700	-3.47794700	-0.12104200
C	0.91345500	-4.54166700	-1.01338700
C	0.84760400	-3.71187500	1.25520400
C	1.17887200	-5.82366000	-0.53491200
H	0.83683300	-4.37333300	-2.08442100
C	1.11233600	-4.99372200	1.72934500
H	0.72084500	-2.89236700	1.95752200
C	1.27849500	-6.05172500	0.83590200
H	1.30757300	-6.64349000	-1.23596000
H	1.18948900	-5.16616400	2.79907800
H	1.48540600	-7.05103900	1.20810900
C	2.64090100	2.38122200	-0.15577800
C	3.47214300	3.05098100	-1.05917700
C	2.79789300	2.60173400	1.21744900
C	4.44814500	3.93098100	-0.59447900
H	3.36087000	2.88804800	-2.12800800
C	3.77407700	3.48102100	1.67779500
H	2.15662300	2.08749700	1.92825500
C	4.60066800	4.14731800	0.77336000
H	5.08884500	4.44698800	-1.30396600
H	3.88862900	3.64641400	2.74528800
H	5.36138400	4.83340500	1.13471100
C	-3.38194500	1.09043000	-0.18860000
C	-4.37370500	1.45359400	-1.10526600
C	-3.65926400	1.14330400	1.18226900
C	-5.62760400	1.86453800	-0.65614300
H	-4.17083700	1.41695600	-2.17243000
C	-4.91271900	1.55448800	1.62706400
H	-2.89637700	0.86234100	1.90336100
C	-5.89898500	1.91570100	0.70943000
H	-6.39163100	2.14433600	-1.37595100
H	-5.11939500	1.59224700	2.69283300
H	-6.87641900	2.23590600	1.05882100

$(CAAC\text{-}H)^+$; E_H = −599.30723087

C	1.20024600	-1.42407400	-0.33522300
C	0.65313900	-2.80840400	-0.23603500
C	1.92812500	-3.62055400	0.15308900
H	1.71983600	-4.33846400	0.96092900
H	2.27801700	-4.19710000	-0.71862800
C	3.02887800	-2.60860900	0.58475200
C	-0.44377900	-2.82267300	0.86585300
H	-1.28488400	-2.15940800	0.60698200
H	-0.05058600	-2.52237700	1.84984300
H	-0.83774200	-3.84837200	0.95318900
C	0.04029700	-3.25186900	-1.58511800
H	-0.30660300	-4.29415100	-1.49672000
H	0.77616500	-3.20583400	-2.40385700
H	-0.82824400	-2.63066400	-1.85762300
C	3.18430000	-2.47532500	2.10834000
H	3.59509300	-3.41759600	2.50404300
H	2.22011300	-2.28860300	2.60725800
H	3.88301500	-1.66613000	2.37313400
C	4.38037600	-2.88808100	-0.07991900

H	5.14821000	-2.15770600	0.21813500
H	4.30100800	-2.90836500	-1.17872200
H	4.72349200	-3.88240200	0.24850900
C	3.09469400	-0.00568400	0.16777700
C	3.27417300	2.15331900	1.24361600
H	2.93506000	2.86587400	2.00029800
C	4.32942700	2.48477400	0.37991800
H	4.81466400	3.46111600	0.46159100
C	4.75962400	1.56885800	-0.59348000
H	5.57077100	1.83313900	-1.27720600
N	2.42897600	-1.28978800	0.06532100
C	2.64705600	0.90352700	1.14186900
H	1.83092500	0.62986400	1.81726100
C	4.14738200	0.31250700	-0.70608700
H	4.46454800	-0.39258200	-1.47776500
H	0.64666800	-0.54643400	-0.69034500

NHC-H$_2$; E_H = −688.72842455

C	2.43891100	0.11483900	0.04631300
C	4.02530000	-1.72473700	0.07770700
H	4.20670400	-2.79614900	0.08195200
C	5.09605600	-0.83584300	0.09731000
H	6.11834500	-1.19959100	0.11692200
C	4.82809800	0.53365200	0.09118400
H	5.64748300	1.24730800	0.10611300
N	1.13553400	0.56277000	0.02139800
C	2.71163200	-1.26596600	0.05249000
H	1.90124100	-1.98725700	0.03771000
C	3.52492800	1.01097600	0.06615100
H	3.35465300	2.08202300	0.06205500
N	-1.13551700	0.56278400	-0.02193200
C	-2.43890700	0.11487100	-0.04645000
C	-2.71166800	-1.26593100	-0.05142300
C	-3.52489800	1.01102200	-0.06706300
C	-4.02535000	-1.72468500	-0.07623200
H	-1.90129900	-1.98723300	-0.03601800
C	-4.82808100	0.53371500	-0.09167100
H	-3.35459100	2.08206700	-0.06390300
C	-5.09608000	-0.83577700	-0.09660200
H	-4.20678600	-2.79609500	-0.07954300
H	-5.64744600	1.24738300	-0.10721600
H	-6.11838100	-1.19951100	-0.11589100
C	-0.67109600	1.87856100	-0.01362100
H	-1.33869200	2.72362600	-0.02682900
C	0.67115200	1.87855300	0.01197000
H	1.33877200	2.72361000	0.02445800
C	-0.00000400	-0.34188600	0.00012800
H	-0.01713000	-0.99223500	0.89449200
H	0.01710200	-0.99301700	-0.89366700

CAAC-H$_2$; E_H = −600.21572526

C	2.38461200	-0.70290600	0.06790800
C	2.27777800	0.72256300	-0.49131000
H	3.03070300	1.39779900	-0.07031900
H	2.44323100	0.68952200	-1.57538500
C	0.84024900	1.22749900	-0.21268300
C	2.72553300	-0.72364000	1.56164300
H	2.83838900	-1.75578900	1.91296000
H	1.94055400	-0.25538700	2.16164000
H	3.66796400	-0.19927500	1.75643800
C	3.40478200	-1.54168600	-0.69751500
H	4.41714300	-1.14585700	-0.55803400
H	3.19071000	-1.54924500	-1.77215400
H	3.40561700	-2.57981400	-0.34533500
C	0.85109900	2.20595100	0.97197800
H	1.47556400	3.07097700	0.72232900
H	1.26342700	1.73681600	1.86831800
H	-0.14239900	2.58317400	1.22188000
C	0.29667400	1.92408500	-1.46678100
H	-0.70353200	2.33884200	-1.32071100
H	0.25001800	1.22077900	-2.30474600
H	0.96377800	2.74652100	-1.74764300
C	-1.26309900	-0.18675200	0.06363100
C	-3.53039400	0.68022700	0.32479700
H	-4.17753400	1.52768300	0.53661400
C	-4.08422800	-0.57270000	0.06748600
H	-5.15989700	-0.71826700	0.07111500
C	-3.21928200	-1.63075400	-0.19304200
H	-3.61663500	-2.62069200	-0.40263500
N	0.11632500	-0.03464300	0.09253300
C	-2.15488300	0.87452100	0.32837600
H	-1.77720900	1.86200000	0.55581300
C	-1.83958300	-1.44803800	-0.20232400
H	-1.20917000	-2.29998100	-0.42752500
C	0.95424600	-1.19356600	-0.15707300
H	0.83682500	-1.57334500	-1.18930900
H	0.69288100	-2.01145300	0.52571600

(NHC-H)$^+$; E_H = −687.94069071

C	-0.00000200	-0.23870400	0.00007700
C	2.44236300	0.07274200	-0.02728600
C	4.14104100	-1.31237200	-0.99389200
H	4.45472000	-2.03600500	-1.73939500
C	5.05373800	-0.82563200	-0.06007600
H	6.08031600	-1.17783100	-0.07321300
C	4.65450700	0.11437000	0.88877900
H	5.36450800	0.48921800	1.61898600
N	1.08549900	0.54030600	-0.00651500
C	2.82481300	-0.85885600	-0.98871900
H	2.11663400	-1.20483400	-1.73632300
C	3.33902100	0.56807000	0.91560800
H	3.01218500	1.27927900	1.66865800
H	-0.00001300	-1.31811600	0.00053000
N	-1.08548700	0.54033400	0.00602200
C	-2.44236000	0.07281700	0.02719200
C	-2.82482500	-0.85796800	0.98940600

C	-3.33901200	0.56737300	-0.91611300
C	-4.14106200	-1.31145300	0.99496500
H	-2.11665100	-1.20333300	1.73729800
C	-4.65450700	0.11372200	-0.88889800
H	-3.01216600	1.27794500	-1.66975900
C	-5.05375300	-0.82547600	0.06074500
H	-4.45475300	-2.03445500	1.74107500
H	-5.36450400	0.48797300	-1.61941600
H	-6.08033900	-1.17764400	0.07418100
C	-0.68040700	1.85839300	0.00375100
H	-1.39029700	2.67057900	0.02411700
C	0.68044700	1.85837500	-0.00535600
H	1.39035500	2.67052800	-0.02640600

(CAAC-H)$^{\bullet}$; E_H = −599.57034013

C	1.10628600	-1.35718400	-0.14143100
C	0.62864600	-2.77929000	-0.09873700
C	1.96204500	-3.55077400	0.02671900
H	1.90502700	-4.37888700	0.74066500
H	2.22754500	-3.97663400	-0.94785000
C	3.05435000	-2.54417800	0.43830100
C	-0.30876700	-3.03605100	1.09408800
H	-1.20443800	-2.40920900	1.02028400
H	0.17913700	-2.81170300	2.04692900
H	-0.63023800	-4.08505900	1.11243300
C	-0.11353400	-3.16147300	-1.38873200
H	-0.39584300	-4.22147700	-1.37681300
H	0.51296500	-2.98323600	-2.26907100
H	-1.03125600	-2.57231600	-1.49848600
C	3.20582600	-2.43513900	1.95924900
H	3.54087600	-3.39068800	2.37608600
H	2.25852900	-2.16296900	2.43321900
H	3.94791100	-1.67265200	2.21871800
C	4.39123700	-2.94638100	-0.17979300
H	5.22151000	-2.34010800	0.19191700
H	4.35775700	-2.88186300	-1.27196900
H	4.60151400	-3.98642600	0.09224600
C	3.11155700	-0.02217400	0.08458500
C	2.99925100	2.28569300	0.84883400
H	2.43785800	3.08071900	1.33301300
C	4.29693800	2.52128000	0.39715400
H	4.75411400	3.49933200	0.51475900
C	4.99500900	1.48217300	-0.22108900
H	5.99964800	1.65183900	-0.59949600
N	2.49172600	-1.27083800	-0.10303600
C	2.41269300	1.03179200	0.69982800
H	1.41095200	0.84845700	1.07665800
C	4.41147400	0.23039700	-0.38867900
H	4.95369600	-0.54124100	-0.92106900
H	0.56043700	-0.55200700	-0.62445500

[PhPH(BH_3)]$^{\bullet}$; E_H = −599.76342720

P	-1.66089000	1.17791100	0.83803600
C	-3.18492400	0.64963600	0.08717100
C	-3.45619600	0.90006900	-1.27235100
C	-4.11894000	-0.07154800	0.85653100
C	-4.64047300	0.44850400	-1.84093500
H	-2.74175500	1.45148300	-1.87754000
C	-5.30074800	-0.51771000	0.27815700
H	-3.91679300	-0.27027200	1.90520300
C	-5.56413000	-0.26017100	-1.06899900
H	-4.84644500	0.65047200	-2.88781800
H	-6.01996600	-1.06686200	0.87836600
H	-6.48861300	-0.61160200	-1.51756000
B	-1.53560100	1.60218800	2.72111000
H	-0.35848600	1.77998100	2.94165800
H	-2.02327300	0.67585000	3.32940300
H	-1.11820200	2.07004500	-0.10833900
H	-2.19541700	2.62826500	2.79308500

M; E_H = −1198.63269258

C	-0.12190200	-2.38136000	-0.78789900
C	-0.08043100	-0.86195300	-0.53893900
C	-2.29512600	-1.61041700	0.13589200
C	-1.31843100	-2.80470300	0.07280600
H	-1.80632500	-3.70110700	-0.31920400
H	-0.98016800	-3.04555300	1.08760700
C	1.15444800	-3.10237000	-0.38540800
H	1.10859900	-4.17267700	-0.61887400
H	2.06080000	-2.70788800	-0.84612700
H	1.23077700	-3.23147700	0.75262600
C	-0.33757500	-2.62965500	-2.28851000
H	0.56275200	-2.37706700	-2.85763300
H	-0.57510700	-3.68169200	-2.47038800
H	-1.15777000	-2.02016200	-2.67392600
C	-2.81018800	-1.47448000	1.57509100
H	-3.56422200	-0.69574000	1.69754700
H	-3.26784500	-2.42268800	1.87500400
H	-1.98975700	-1.26520700	2.26897300
C	-3.46105800	-1.82863400	-0.83841700
H	-4.03624200	-2.70085800	-0.51279100
H	-4.14519600	-0.98022900	-0.88125700
H	-3.10254200	-2.01909900	-1.85229600
N	-1.42498100	-0.47351200	-0.28604100
P	1.12957400	-0.45888900	0.91263300
H	0.34344700	0.16175100	1.89546200
B	1.86835100	-2.13487300	1.53680900
H	1.29302200	-2.62374700	2.46348300
H	3.03687200	-2.29538400	1.38817100
C	2.41539300	0.73390000	0.49401400
C	2.31394800	2.05768400	0.94159800
C	3.51043700	0.33462500	-0.28310400
C	3.30614200	2.97458800	0.60795600
H	1.46841200	2.37406600	1.54588500
C	4.49156500	1.26237700	-0.62015100
H	3.61203400	-0.69339900	-0.61955400
C	4.39061100	2.57978400	-0.17477500

H	3.23068400	3.99826400	0.96130800
H	5.33882100	0.95215900	-1.22369200
H	5.16150600	3.29910400	-0.43398500
C	-1.79503600	0.87169200	-0.34652000
C	-0.85460900	1.85316700	-0.72102500
C	-3.09712100	1.31718100	-0.04318100
C	-1.20235700	3.19987000	-0.79383700
H	0.16454000	1.58781300	-0.97466200
C	-3.43128700	2.66438600	-0.11999600
H	-3.86769900	0.62117500	0.25278500
C	-2.49250200	3.62415400	-0.49448100
H	-0.44336100	3.91845400	-1.09079500
H	-4.44833300	2.96139000	0.12050400
H	-2.76133500	4.67415300	-0.54995800
H	0.35729000	-0.33560400	-1.39521200

M’; $E_H = -1198.62953343$

C	0.03944000	2.40111900	0.42591000
C	-0.19275100	0.95976200	0.93890500
C	-1.19094800	1.06103600	-1.27443000
C	-0.20440700	2.23192900	-1.08390200
H	-0.58061300	3.15004000	-1.54403100
H	0.74018200	1.98431900	-1.58182400
C	1.44903400	2.92081600	0.70082000
H	1.51605200	4.01292500	0.64645400
H	1.72270300	2.78418800	1.79759800
H	2.20280900	2.52718200	0.01816100
C	-0.97586400	3.33249300	1.10027900
H	-0.72452600	3.47832800	2.15627500
H	-0.97724100	4.31133700	0.61148300
H	-1.98987500	2.92942300	1.05181000
C	-0.78801200	0.24791800	-2.50779100
H	-1.51953900	-0.53271400	-2.73394600
H	-0.74025700	0.91083900	-3.37843500
H	0.19409100	-0.21418100	-2.37106200
C	-2.63995300	1.52823800	-1.44442300
H	-2.73647300	2.09175300	-2.37768000
H	-3.31719300	0.67045100	-1.50013100
H	-2.97013300	2.16830200	-0.62307300
N	-1.02599100	0.30569600	0.00183800
P	1.48218200	0.08776600	1.29566700
C	-1.95013700	-0.68230800	0.42755000
C	-2.73801200	-0.49382900	1.57231700
C	-2.09397000	-1.87322400	-0.29613000
C	-3.63953900	-1.47217800	1.98245800
H	-2.66477600	0.43009400	2.13937900
C	-3.01557100	-2.83919900	0.10379500
H	-1.47344300	-2.04819200	-1.16794100
C	-3.78820400	-2.64782200	1.24732500
H	-4.24123400	-1.30466700	2.87129400
H	-3.11476600	-3.75331000	-0.47477000
H	-4.49789900	-3.40621800	1.56387200
H	1.15195700	-0.92133000	2.21425200
H	3.64577500	1.67434000	1.77412600
H	2.19280500	1.70970700	3.26417800
B	2.54078000	1.45380100	2.15066200
C	2.21277400	-0.74268700	-0.12157800
C	1.68350900	-1.97557400	-0.52779600
C	3.27221900	-0.16398700	-0.83155000
C	2.21214100	-2.61886200	-1.64300400
H	0.86913800	-2.43731000	0.02360300
C	3.79093200	-0.81356300	-1.94792400
H	3.71006500	0.77690100	-0.51169000
C	3.26085100	-2.03741000	-2.35458600
H	1.80517800	-3.57624500	-1.95353100
H	4.61550800	-0.36605100	-2.49393500
H	3.67138900	-2.54292900	-3.22346300
H	-0.60874900	0.97033900	1.95497300

Supplementary References

1. Jafarpour, L., Stevens, E. D. & Nolan, S. P. A sterically demanding nucleophilic carbene: 1,3-bis(2,6-diisopropylphenyl)imidazol-2-ylidene). Thermochemistry and catalytic application in olefin metathesis. *J. Organomet. Chem.* **606,** 49–54 (2000).

2. Jazzar, R., Dewhurst, R. D., Bourg, J-B., Donnadieu, B., Canac, Y. & Bertrand, G. Intramolecular 'Hydroiminiumation' of Alkenes: Application to the Synthesis of Conjugate Acids of Cyclic Alkyl Amino Carbenes (CAACs). *Angew. Chem. Int. Ed.* **46,** 2899–2902 (2007).

3. Hurtado, M. Yánez, M., Herrero, R., Guerrero, A., Dávalos, J., Abboud, J-L. M., Khater, B. & Guillemin, J-C. The Ever-Surprising Chemistry of Boron: Enhanced Acidity of Phosphine·Boranes. *Chem. Eur. J.* **15,** 4622–4629 (2009).

4. Pelczar, E. M., Nytko, E. A., Zhuravel, M. A., Smith, J. M., Glueck, D. S., Sommer, R., Incarvito, C. D. & Rheingold, A. L. Synthesis and structure of platinum and palladium complexes of dimesitylphosphine. *Polyhedron* **21,** 2409–2419 (2002).

5. Lebel, H., Morin, S. & Paquet, V. Alkylation of Phosphine Boranes by Phase-Transfer Catalysis. *Org. Lett.* **5,** 2347–2349 (2003).

6. Nguyen, D. H., Lauréano, H., Jugé, S., Kalck, P., Daran, J-C., Coppel, Y., Urrutigoity, M. & Gouygou, M. First Dibenzophospholyl(diphenylphosphino)methane - Borane Hybrid P-(η_2-BH_3) Ligand: Synthesis and Rhodium(I) Complex. *Organometallics* **28,** 6288–6292 (2009).

7. Frey, G. D., Masuda, J. D., Donnadieu, B. & Bertrand, G. Activation of Si-H, B-H, and P-H bonds at a Single Nonmetal Center. *Angew. Chem. Int. Ed.* **49,** 9444–9447 (2010).

8. Dorn, H., Singh, R. A., Massey, J. A., Nelson, J. M., Jaska, C. A., Lough, A. J. Manners, I. Transition Metal-Catalyzed Formation of Phosphorus-Boron Bonds: A New Route to Phosphinoborane Rings, Chains, and Macromolecules. *J. Am. Chem. Soc.* **122,** 6669–6678 (2000).

9. Burck, S., Gudat, D., Nieger, M. & Vinduš, D. Increasing the Lability of Polarised Phosphorus-Phosphorus Bonds. *Eur. J. Inorg. Chem.* **2,** 704–707 (2008).

10. Marquardt, C., Jurca, T., Schwan, K-C., Stauber, A., Virovets, A. V., Whittell, G. R., Manners, I. & Scheer, M. Metal-Free Addition/Head-to-Tail Polymerization of Transient Phosphinoboranes, RPH-BH_2: A Route to Poly(alkylphosphinoboranes). *Angew. Chem. Int. Ed.* **54,** 13782–13786 (2015).

11. Hooper, T. N., Weller, A. S., Beattie, N. A. & Macgregor, S. A. Dehydrocoupling of phosphine-boranes using the [RhCp*Me(PMe_3)(CH_2Cl_2)][BAr^F_4] precatalyst: stoichiometric and catalytic studies. *Chem. Sci.* **7,** 2414–2426 (2016).

12. Frisch, M. J., Trucks, G. W., Schlegel, H. B., Scuseria, G. E., Robb, M. A., Cheeseman, J. R., Scalmani, G., Barone, V., Mennucci, B., Petersson, G. A., Nakatsuji, H., Caricato, M., Li, X., Hratchian, H. P., Izmaylov, A. F., Bloino, J., Zheng, G., Sonnenberg, J. L., Hada, M., Ehara, M., Toyota, K., Fukuda, R., Hasegawa, J., Ishida, M., Nakajima, T., Honda, Y., Kitao, O., Nakai, H., Vreven, T., Montgomery Jr., J. A., Peralta, J. E., Ogliaro, F., Bearpark, M., Heyd, J. J,, Brothers, E., Kudin, K. N., Staroverov, V. N., Kobayashi, R., Normand, J., Raghavachari, K., Rendell, A., Burant, J. C., Iyengar, S. S., Tomasi, J., Cossi, M., Rega, N., Millam, M. J., Klene, M., Knox, J. E., Cross, J. B., Bakken, V., Adamo, C., Jaramillo, J., Gomperts, R., Stratmann, R. E., Yazyev, O., Austin, A. J., Cammi, R., Pomelli, C., Ochterski, J. W., Martin, R. L., Morokuma, K., Zakrzewski, V. G., Voth, G.A., Salvador, P., Dannenberg, J. J., Dapprich, S., Daniels, A. D., Farkas, Ö., Foresman, J. B., Ortiz, J. V., Cioslowski, J. & Fox, D. J. *Gaussian 09, Revision D.01*. (Gaussian, 2009).

13. Adamo, C. & Barone, B. Toward reliable density functional methods without adjustable parameters: The PBE0 model. *J. Chem. Phys.* **110,** 6158–6170 (1999).

14. Perdew, J. P., Ernzerhof, M. & Burke, K. Rationale for mixing exact exchange with density functional approximations. *J. Chem. Phys.* **105,** 9982–9985 (1996).

15. Ditchfield, R., Hehre, W. J. & Pople, J. A. Self-Consistent Molecular-Orbital Methods. IX. An Extended Gaussian-Type Basis for Molecular-Orbital Studies of Organic Molecules. *J. Chem. Phys.* **54,** 724–728 (1971).

16. Hehre, W. J., Ditchfield, R. & Pople, J. A. Self-Consistent Molecular Orbital Methods. XII. Further Extensions of Gaussian- Type Basis Sets for Use in Molecular Orbital Studies of Organic Molecules. *J. Chem. Phys.* **56,** 2257–2261 (1972).

17. Hariharan, P. C. & Pople, J. A. Accuracy of AH_n equilibrium geometries by single determinant molecular orbital theory. *Mol. Phys.* **27,** 209–214 (1974).

18. Hariharan, P. C. & Pople, J. A. The influence of polarization functions on molecular orbital hydrogenation energies. *Theor. Chem. Acc.* **28,** 213–222 (1973).

19. Gordon, M. S. The isomers of silacyclopropane. *Chem. Phys. Lett.* **76,** 163–168 (1980).

20. Francl, M. M., Pietro, W. J., Hehre, W. J., Binkley, J. S., Gordon, M. S., DeFrees, J. & Pople, J. A. Self-consistent molecular orbital methods. XXIII. A polarization-type basis set for second-row elements. *J. Chem. Phys.* **77,** 3654–3665 (1982).

21. Lebedev, V. I. & Skorokhodov, A. L. Quadrature Formulas of Orders 41, 47 and 53 for the Sphere. *Russ. Acad. Sci. Dokl. Math* **45,** 587 (1992).

22. Miertuš, S., Scrocco, E. & Tomasi, J. Electrostatic interaction of a solute with a continuum. A direct utilizaion of AB initio molecular potentials for the prevision of solvent effects. *Chem. Phys.* **55,** 117–129 (1981).

23. Pascual-ahuir, J. L.; Silla, E. & Tunon, I. GEPOL: An improved description of molecular surfaces. III. A new algorithm for the computation of a solvent-excluding surface. *J.*

Comp. Chem. **15,** 1127–1138 (1994).

24. Cancès, E., Mennucci, B. & Tomasi, J. A new integral equation formalism for the polarizable continuum model: Theoretical background and applications to Isotropic and anisotropic dielectrics. *J. Chem. Phys.* **107,** 3032–3041 (1997).

25. Mennucci, B., Cammi, R. & Tomasi, J. Excited states and solvatochromic shifts within a nonequilibrium solvation approach: A new formulation of the integral equation formalism method at the self-consistent field, configuration interaction, and multiconfiguration self-consistent field level. *J. Chem. Phys.* **109,** 2798–2807 (1998).

26. Fukui, K. The Path of Chemical Reactions - the IRC Approach. *Acc. Chem. Res.* **14,** 363–368 (1981).

27. Dykstra, C. E., Frenking, G., Sim, K. S. & Scuseria, G. *Theory and Applications of Computational Chemistry: The First 40 Years*. (Elsevier, Amsterdam, 2005).

28. Bruker-AXS Apex II software, Madison, WI, 2008.

29. G. M. Sheldrick, SADABS V2012/1, University of Göttingen, Germany.

30. Sheldrick, G. M. SHELXT - Integrated space-group and crystal-structure determination. *Acta Crystallogr. Sect. A Found. Crystallogr.* **71,** 3–8 (2015).

31. Sheldrick, G. M. Crystal structure refinement with SHELXL. *Acta Crystallogr. Sect. C Struct. Chem.* **71,** 3–8 (2015).

32. Dolomanov, O. V., Bourhis, L. J., Gildea, R. J., Howard, J. A. K. & Puschmann, H. OLEX2: A complete structure solution, refinement and analysis program. *J. Appl. Crystallogr.* **42,** 339–341 (2009).
